# Advancing human-centric AI for robust X-ray analysis through holistic self-supervised learning

**DOI:** 10.1038/s41467-026-76076-4

**Published:** 2026-08-20

**Authors:** Théo Moutakanni, Piotr Bojanowski, Guillaume Chassagnon, Céline Hudelot, Armand Joulin, Yann LeCun, Matthew Muckley, Maxime Oquab, Marie-Pierre Revel, Maria Vakalopoulou

**Affiliations:** 1 FAIR at Meta, Paris, France; 2 MICS, CentraleSupélec, Université Paris-Saclay, Gif-sur-Yvette, France; 3 Department of Radiology, Hôpital Cochin, AP-HP. Centre Université Paris Cité, Paris, France; 4 Université Paris Cité, Paris, France; 5 Cancer Data Science Unit, CentraleSupélec, Gustave Roussy, INSERM, IHU PRISM, Université Paris-Saclay, Gif-sur-Yvette, France

**Keywords:** Medical research, Diagnosis

## Abstract

Foundational artificial intelligence models are gaining traction in various applications, including medical fields like radiology. However, medical foundation models are often tested on limited tasks, leaving their generalisability and biases unexplored. Here we introduce RayDINO, a large-scale self-supervised visual encoder for chest X-rays trained on 840,000 images and evaluated on 82,000 images sourced from 12 publicly available datasets. We compare RayDINO to previous state-of-the-art models across nine radiology tasks, from classification and dense segmentation to text generation, and provide an in-depth analysis of population, age and sex biases of our model. Our findings suggest that self-supervision enables patient-centric analysis useful in clinical workflows, allowing for a more holistic interpretation of X-rays. With RayDINO and small task-specific adapters, we reach state-of-the-art results and improve generalization to unseen populations while mitigating bias, illustrating the versatility and robustness of foundation models.

## Abstract (web summary)

In this study, Moutakanni and colleagues introduce RayDINO, a self-supervised chest X-ray foundation model trained on 840,000 images, that outperforms supervised methods across nine radiology tasks and shows improved generalization and reduced bias across demographics.

## Introduction

Clinical workflows require a holistic analysis and interpretation of patient data^1^. While humans excel at contextualizing information, current machine learning models often fall short, extracting only the minimal information necessary to solve a training task defined by supervision, without considering the broader context. This is particularly evident in radiology, where chest X-rays, one of the most common and easily accessible types of exams, are used to diagnose a variety of conditions affecting the lung and adjacent organs^2^. However, providing separate artificial intelligence (AI) models for each organ and anomaly visible in chest X-rays can be time consuming or even unfeasible since it requires a lot more manual work in scenarios where access to annotated data is difficult.

Foundation models offer a solution to these challenges. They are large-scale, pre-trained models that produce generalizable and powerful representations of data, useful for a wide range of more specific downstream tasks. In contrast to supervised learning, they are trained in a more general manner using large amounts of unlabeled data, thus capturing task-agnostic characteristics and leading to more holistic^3^, patient-centered, and unbiased representations^4,5^. These models are becoming increasingly popular in medical tasks, and many recent articles report good performances of such models^6–8^.

Many foundation models often rely heavily on natural language for their training^9^. These algorithms learn image features by embedding paired images and textual descriptions in a shared feature space. However, the structure and complexity of language used in medical reports differ significantly from image captions derived from web data, and training such models for medical tasks can be complex due to their heterogeneous nature. This is highlighted in recent studies, which have raised concerns about the quality of automated text-based data labeling^10^ and the reliability of AI systems trained on reports from medical images^11^.

Moreover, country-specific or brittle guidelines^12–14^ shaping text reports can hinder the robustness of foundation models when applied to new populations. In this study, we propose an approach that circumvents these annotation-related challenges by using self-supervised learning, based only on image-based radiology data.

In this study, we introduce RayDINO, the largest vision transformer (ViT)^15^ model of 307M parameters trained on a large dataset of 840k images compiled from four datasets of public X-ray images. More specifically, RayDINO is trained on four datasets originating from the USA (MIMIC^16^, CheXpert^17^, NIH^18^) and Spain (PadChest^19^). Our model generates holistic representations of a compact size of 1024 embedding dimensions for each chest X-rays using the self-supervised DINOv2 pretraining method^3^.

## Results

**Fig. 1:**
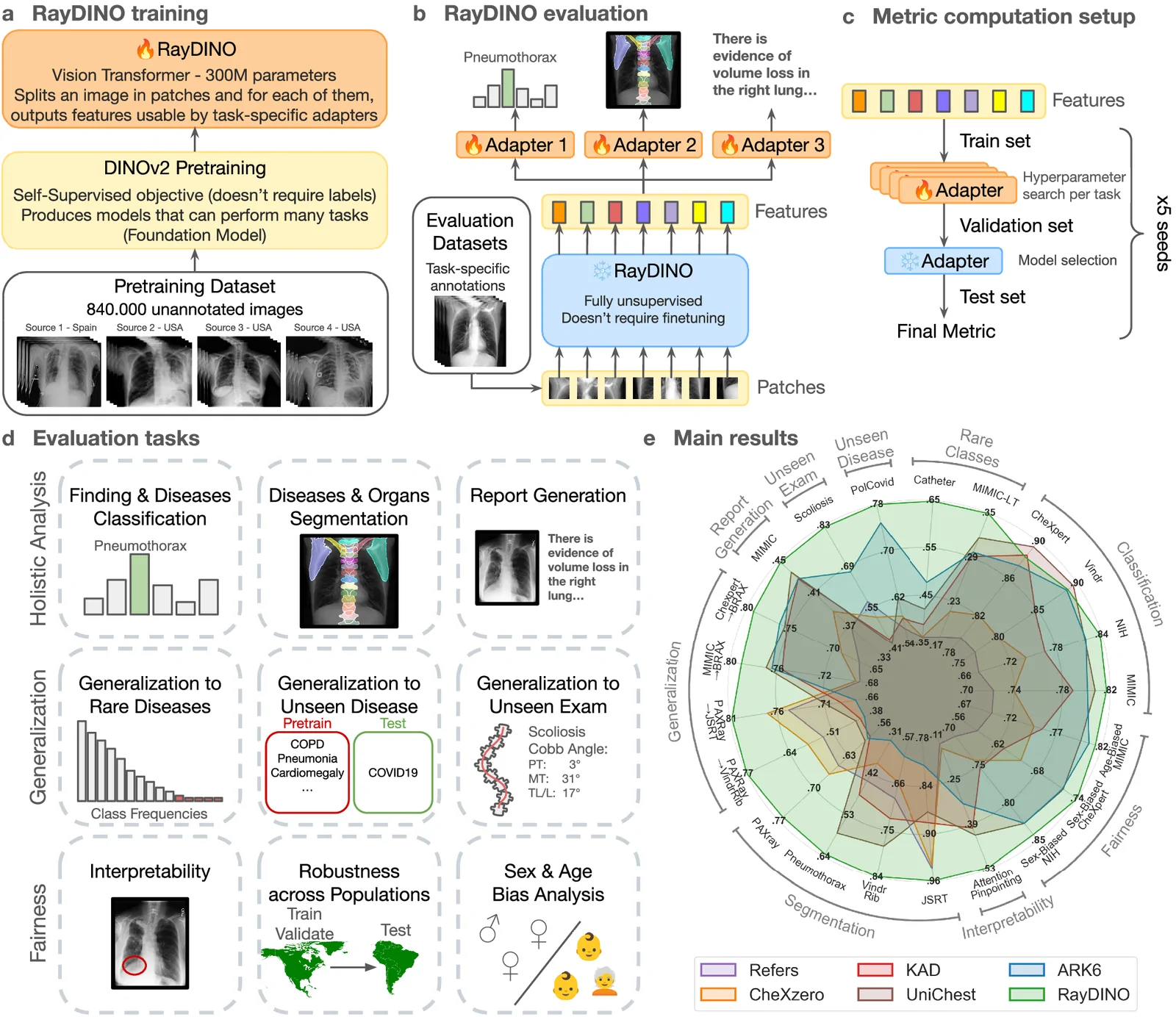
We introduce RayDINO, a foundation model for holistic and fairer analysis of chest X-rays. **a** RayDINO is a vision transformer trained using DINOv2 objectives on four different datasets from the USA and Europe. **b** We evaluate RayDINO and other models as-is on all tasks without any modification or specialization of their parameters by using very small task-specific adapters. **c** Our supervised adapters are trained and validated for each task using a hyperparameters search. We repeated this setup 5 times to produce confidence intervals and compare models. **d** RayDINO is evaluated on tasks divided into three categories. **e** RayDINO consistently delivers excellent performance on 21 benchmarks (AUROC for Classification and Fairness, macro Accuracy for interpretability, mDice for Segmentation, AUROC for BRAX Generalization, and mDice for PAXRay Generalization, CheXbert vector similarity for Report Generation, Pearson’s correlation for Unseen Exam, macro Accuracy for Unseen Disease, AUPRC/Area Under Precision Recall Curve for Rare Classes).

We evaluate RayDINO’s performance (Fig. 1a) by using task-specific adapters on top of the obtained frozen features (Fig. 1b,c) using eleven publicly available datasets from seven countries across four continents, both in internal and external settings. We report an extensive evaluation on nine medical-imaging related tasks (Fig. 1d) divided into 21 benchmarks comprising more than 200 classes (Fig. 1e). We compare our model with five state-of-the-art foundation models trained using supervised annotations: Refers^20^, CheXzero^21^, KAD-512^22^, UniChest^23^, and ARK6^24^.

We emphasize that these models are fully fine-tuned end-to-end using in-domain labels or reports, while our approach uses a frozen backbone pre-trained without any human supervision. This establishes a challenging benchmark for RayDINO, which has significantly fewer supervised parameters than the baselines (Table 1).

**Table 1:** Parameter size of the different backbones we use in this study along with the task-specific adapters

Model	Architecture	Num. Params	Pre-Train Datasets
*Benchmarked Backbones*			
UniChest^23^	ResNet50	55 M	CheXpert, MIMIC, NIH, VinDr
KAD^22^	ResNet50	55 M	MIMIC
ARK6^24^	Swin-B	91 M	CheXpert, MIMIC, NIH, VinDr, Pneumonia, Shenzhen
CheXzero^21^	ViT-B	86 M	MIMIC
Refers^20^	ViT-B	86 M	MIMIC
RayDINO (ours)	ViT-L	307 M	CheXpert, MIMIC, NIH, PadChest
*Task-Specific Adapters*			
Classification / Regression Adapter	2 layers MLP + ReLU	0.1–1 M	-
Segmentation Adapter	UperNet^59^	49 M	-
Text Adapter	LLM (Llama^53^)	7 B	-

### Unified approach for comprehensive radiology interpretation: classification, segmentation, and report generation

**Fig. 2:**
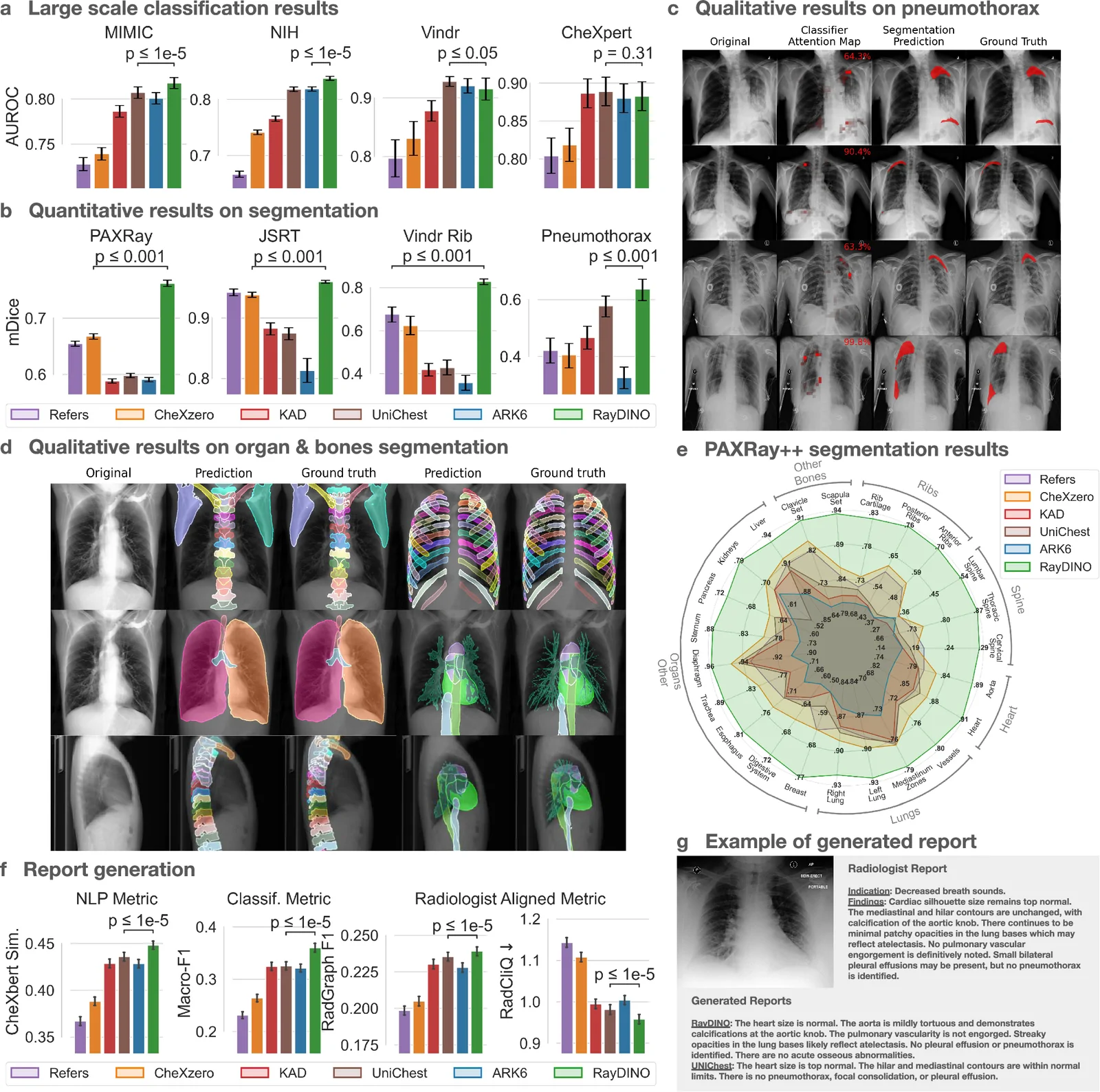
RayDINO’s holistic analysis of chest X-rays. **a** AUROC comparisons on 4 classification datasets from the USA and Vietnam including 38 different findings (MIMIC *n* = 5159 test images, NIH *n* = 25, 596, CheXpert *n* = 668, VinDr *n* = 3000). **b** mDice comparisons on 4 segmentation datasets from Japan, Vietnam, the USA, China and multiple other countries, including 157 categories (JSRT *n* = 50, VinDr Rib *n* = 49, PAXRay++ *n* = 1, 476, Pneumothorax *n* = 3, 205). **c** Qualitative visualisation of RayDINO’s prediction on four pneumothorax cases. 1^st^column: original image. 2^nd^column: interpretable attention maps showing where the classifier trained without pixel supervision is looking at. 3^rd^column segmentation prediction trained using pixel supervision. 4^th^column: radiologist ground-truth. **d** Qualitative results for organ and bone segmentation on the PAXRay++ dataset. **e** mDice segmentation comparisons macro-averaged per organs and bones (*n* = 1, 476 test images). **f** Report generation comparison including natural language processing metric, classification-based metric and radiologist-aligned metrics (*n* = 3, 822 test reports). **g** A chest X-ray along with a radiologist report and two generated reports by RayDINO and UNIChest. In **a**, **b**, **e** and **f**, data are presented as mean values ±95% confidence intervals obtained via bootstrapping (1000 resamples, 2.5th–97.5th percentiles). Statistical significance was assessed using a two-sided DeLong test for AUROC comparisons and a two-sided paired permutation test (1000 bootstraps) for all other metrics; no adjustments were made for multiple comparisons. Exact p-values are reported in the main text.

To demonstrate the comprehensive analysis of X-ray images that our model, RayDINO, can perform, we tested it against a broad range of established benchmarks. For each task, we trained and validated multiple task-specific adapters on top of the frozen-weights models using the exact same data and exhaustive hyperparameter grid search (as explained in the Experimental Protocols section of Methods) and then took the best performing model on the validation set. We replicated this five times for all models using different seeds and reported the average and 95% confidence interval (CI) of the performance of the test set. To compare RayDINO against the best performing baseline, we reported p-values of a DeLong test^25^ for AUROC and a paired permutation test for other metrics. More details about our metrics are available in the Evaluation Metrics section of Methods. The results of these evaluations are detailed in the following section and summarized in Fig. 2, providing both qualitative and quantitative insights.

In our first set of experiments, we performed multilabel classification on four datasets, including a total of 38 different findings (more details in Methods). Fig. 2a indicates that RayDINO significantly outperforms the best alternatives on the MIMIC dataset with 0.818 AUROC (95%CI [0.812–0.824],  + 0.011 above 0.807 of UniChest, *p*≤1e − 4) and the NIH dataset with 0.837 AUROC (95%CI [0.834–0.841],  + 0.018 above 0.819 of ARK6, *p*≤1e − 4). Furthermore, despite other compared models being supervised on this specific task and RayDINO having 100x less supervised parameters (Table 1), it remains comparable on CheXpert with 0.883 AUROC (95%CI [0.864–0.902],  − . 006 below 0.889 of UniChest, *p* = 0.312) and on Vindr which is out-of-domain for RayDINO, contrary to ARK6 and UniChest, with 0.915 AUROC (95%CI [0.896–0.934],  −0.013 below 0.928 of UniChest, *p*≤0.05).

Then, we evaluated RayDINO on segmentation tasks on four datasets, three of which are on soft organs and bone segmentation to evaluate anatomical understanding while the other is on pneumothorax segmentation to show the precise localization capabilities of RayDINO’s features. We report results in Fig. 2b and show that it exhibits superior performance compared to all alternatives, on all benchmarks. For bone and organ segmentation, our model outperforms all competitors with . 959 mDice on JSRT (95%CI [0.957–0.962],  +0.021 above 0.938 of CheXzero, *p*≤1e − 3), 0.828 mDice on Vindr Rib (95%CI [0.814–0.840],  +0.152 above 0.676 of Refers, *p*≤1e − 3), and 0.762 mDice on the more challenging PAXRay++ dataset (95%CI [0.756–0.767],  +0.094 above 0.668 of CheXzero, *p*≤1e − 3). We present PAXRay results macro-averaged per organ in Fig. 2e and demonstrate that RayDINO surpasses other models on all organs (*p*≤1e − 3). RayDINO also performs significantly better at segmenting pneumothorax with 0.637 mDice (95%CI [0.596–0.671],  + 0.060 above 0.577 of UniChest, *p*≤1e − 3). We demonstrate the quality of RayDINO predictions with ground-truth segmentations on pneumothorax localization in Fig. 2c and on organ segmentation in Fig. 2d.

Finally, we challenged RayDINO for radiology report generation on MIMIC^16^ reports. This task requires a comprehensive understanding of images and is crucial with the advent of advanced systems able to interact with patients and radiologists through text. We want to emphasize that RayDINO is an image-based foundation model contrary to KAD, UniChest, and CheXzero compared methods. As shown in Fig. 2f, RayDINO outperforms UniChest and all other models on Natural Language Processing metrics (0.448 CheXbert similarity score, 95%CI [0.440–0.456],  +0.012 above 0.436, *p*≤1e − 5), on classification metrics (0.361 macro F1, 95%CI [0.344–0.376],  + 0.034 above . 327 of UniChest, *p*≤1e − 5) and on radiologist aligned metrics (0.239 RadGraph-F1, 95%CI [0.233–0.246],  + 0.004 above 0.235, *p*≤1e − 5 and 3.07 RadCliQ, lower is better, 95%CI [3.04–3.11],  − 0.03, *p*≤1e − 5). We show in Supplementary Table 1 that RayDINO even outperformed the specialized model MAIRA-1^26^ in radiologist aligned metrics (CheXbert similarity, RadCliQ and RadGraph-F1). An example of a generated report is presented in Fig. 2g.

### Harnessing Self-Supervised Learning for the Identification of Rare or Under-Represented Classes

We tested RayDINO more thoroughly by evaluating its performance in real-life medical and clinical situations. We focused on its ability to deal with rare health conditions that cannot be annotated on thousands of images. This assessment is crucial to determine the practical utility of our system in routine clinical practice and its potential for large-scale deployment.

**Fig. 3:**
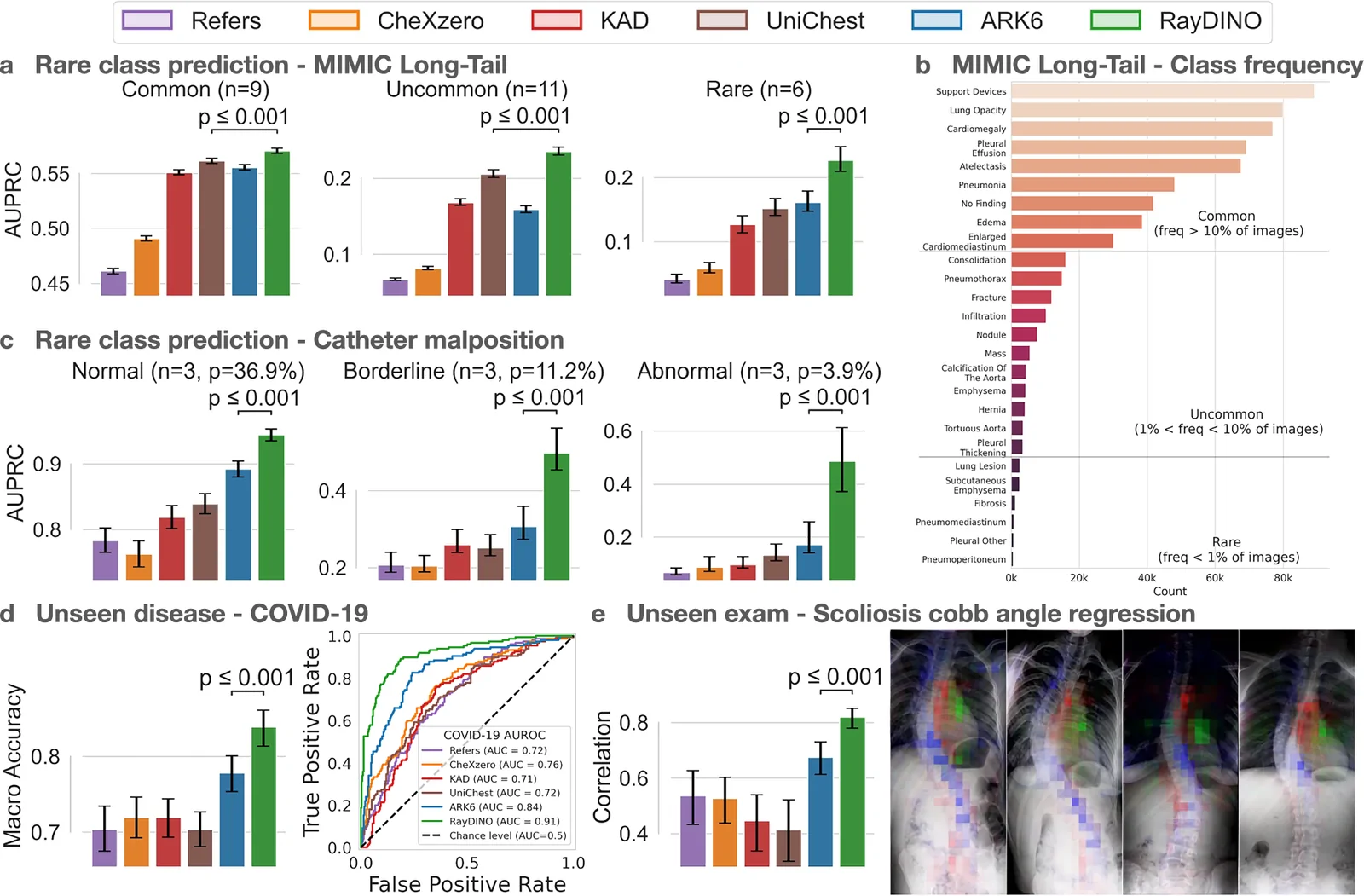
Generalization evaluation on rare or unseen diseases and on unseen exam. **a** AUPRC comparisons of RayDINO against other models on long-tailed findings grouped by frequency: Common (*freq* > 10%, *n* = 9 classes), Uncommon (1% < *freq* < 10%, *n* = 11 classes) and Rare (*freq* < 1%, *n* = 6 classes). Test set: *n* = 75, 492 images from MIMIC Long-Tail. **b** Class distribution on the long-tail evaluations sorted by frequency. **c** AUPRC comparisons on catheter malposition prediction (*n* = 3009 test images, 9 categories grouped into Normal, Borderline and Abnormal). **d** Macro Accuracy comparison on Normal vs Pneumothorax vs COVID-19 classification (*n* = 1315 test images) and ROC curves for the COVID-19 disease. **e** Pearson’s correlation coefficient comparison on Cobb angle regression using spinal exams on *n* = 128 patients with scoliosis and the regression model’s multi-head attention maps for interpretability. In **a**, **c**, **d** and **e**, data are presented as mean values  ± 95% confidence intervals obtained via bootstrapping (1000 resamples, 2.5th–97.5th percentiles). Statistical significance was assessed using a two-sided paired permutation test (1000 bootstraps); no adjustments were made for multiple comparisons. Exact p-values are reported in the main text.

First, for radiology interpretation, we tested our method on labels with different frequencies and focus on the rare classes called the tail (MIMIC Long-Tail dataset^27^). We show results in Fig. 3a. This dataset with 26 classes contains very rare labels with less than 1% of positive images. Details about classes’ frequency are available in Fig. 3b. We split them based on their prevalence in the training data and group them into three bins: Common (*freq* > 10%, *n* = 9 classes), Uncommon (1% < *freq* < 10%, *n* = 11 classes), and Rare (*freq* < 1%, *n* = 6 classes). We report macro AUPRC on each bin. RayDINO improves over the other state-of-the-art methods for common classes (0.570 AUPRC, 95%CI [0.568–0.573],  + 0.009 above 0.561 of UNIChest, *p*≤1e − 3), but more interestingly our model significantly outperforms all competitors on uncommon diseases (0.235 AUPRC, 95%CI [0.231–0.241],  + 0.030 above 0.205 of UNIChest, *p*≤1e − 3) and is way ahead on rare classes (0.227 AUPRC, 95%CI [0.210–0.248], +0.066 above 0.161 of ARK6, *p*≤1e − 3). This showcases RayDINO’s ability to capture rare clinical characteristics.

In addition, we tested our method on catheter position prediction^28^. We split the catheter categories (endotracheal tube, nasogastric tube and central venous catheter) into three groups based on the correctness of their position according to medical doctors: Normal (*freq* = 36.9%), Borderline (*freq* = 11.2%) and Abnormal (*freq* = 3.9%). Catheter malpositions occur rarely in clinical settings, but are a serious source of complications for patients^29^. This is reflected in the distribution of our dataset, where the Abnormal class is rare but is also the most important one. We present the results in Fig. 3c. RayDINO outperforms alternatives by a significant margin on Normal (0.944 AUPRC, 95%CI [0.934–0.952],  + 0.052 above 0.892 of ARK6, *p*≤1e − 3), Borderline (0.498 AUPRC, 95%CI [0.453–0.561],  + 0.191 above 0.307 of ARK6, *p*≤1e − 3) and Abnormal catheters (0.486 AUPRC, 95%CI [0.372–0.612],  + 0.315 above 0.171 of ARK6, *p*≤1e − 3). Notably, RayDINO achieves nearly triple the performances of the best alternative on abnormal positions, highlighting its effectiveness in identifying critical clinical scenarios.

### Out-of-domain task performance: exploring new diseases and exams

All evaluations described above are related to diseases and exams that potentially occur in the training data of the foundation model. In order to evaluate our model on a real out-of-distribution problem, we included a recent disease that the model had never seen during the pretraining phase (using the POLCOVID dataset^30^). We classify images between categories Normal, Pneumonia and COVID-19 and show results in Fig. 3d and Supplementary Fig. 2. We observe that RayDINO reaches 0.839 macro accuracy (95%CI [0.813–0.861]). Our model significantly outperforms all other models ( + 0.061 above 0.778 of ARK6, *p*≤1e − 3) and is even better for the COVID-19 disease reaching 0.910 AUROC (95%CI [0.900–0.920],  + 0.070 above 0.840 of ARK6, *p*≤1e − 5).

We considered another out-of-distribution task by regressing spine curvature of 609 patients with scoliosis^31,32^ using the Scoliosis dataset. Radiography for spine curvature estimation differ from classical chest X-rays in term of field-of-view and aspect ratio and we used spinal X-rays that are a new exam not present in the training datasets. We observe in Fig. 3e that RayDINO achieves a macro Pearson’s correlation coefficient of 0.820 (95%CI [0.780–0.851]) across the 3 Cobb angles, clearly outperforming alternatives with  + 0.145 points over 0.675 of the best competitor ARK6 (*p*≤1e − 3). We show in Supplementary Fig. 3 that RayDINO’s predictions are better for the main thoracic (MT) angle with a *r*^2^ of 0.90 while being less precise for the proximal thoracic (PT) and thoraco-lumbar (TL/L) angles. RayDINO achieves strong performances despite the fact that the field-of-view differs greatly from classical chest X-rays and that the regression adapter is trained on few hundreds of patient.

### Robustness to patient demographics and superior generalization in clinical settings

**Fig. 4:**
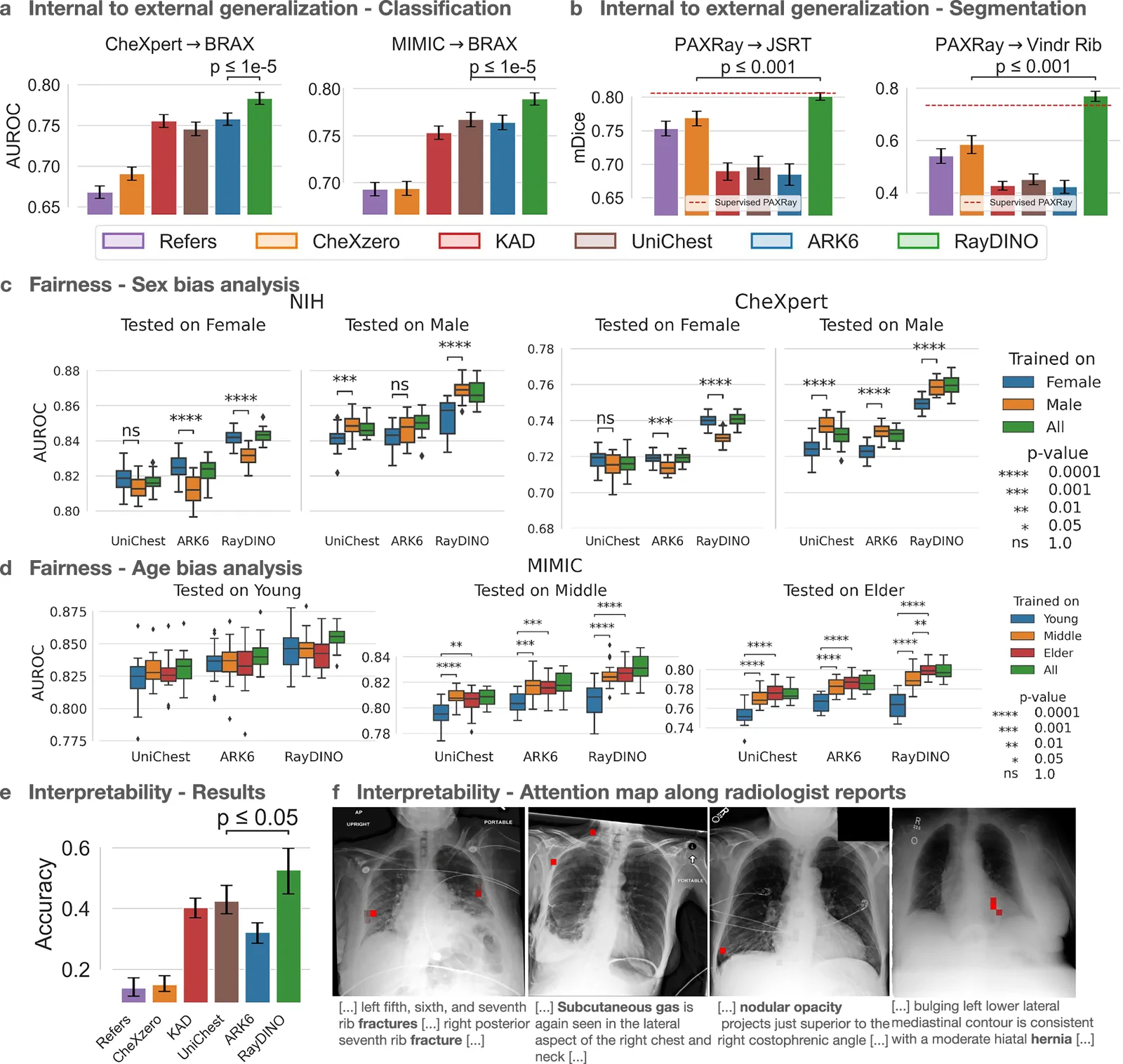
Interpretability and Fairness evaluation of RayDINO between different demographics. **a** AUROC comparisons by training and validating a classifier on MIMIC or CheXpert (USA) and testing it on BRAX (Brazil, *n* = 40, 967 test images). **b** mDice comparisons by training and validating a segmentation head on PAXRay++, a synthetic dataset generated from 2D CT-scan projections (mainly the USA and China), and testing it on real world datasets JSRT (Japan, *n* = 50) and Vindr Rib (Vietnam, *n* = 49). **c** AUROC fairness comparisons by training a classifier on one sex-specific split and testing it on the other split of NIH and CheXpert datasets (*n* = 20 cross-validation folds). Box plots show the median (centre line), interquartile range (box bounds, 25th–75th percentiles), whiskers extending to 1.5 × interquartile range, and individual outliers. *p*-values obtained with a two-sided Mann-Whitney test to assess performance disparities when training with the same sex versus a different sex than the test set. **d** AUROC fairness comparisons by training a classifier on one age-specific split and testing it on the two other splits of the MIMIC dataset (*n* = 20 cross-validation folds). Box plot elements are defined as in (**c**). **e** Macro Accuracy interpretability comparisons by training one classifier for each class of Vindr (*n* = 3 000 test images) and by looking if the position where the attention is looking the most matches the radiologist bounding box on the test set. **f** Attention maps comparison with radiologist reports to demonstrate the accurate localization of RayDINO’s explainable classifiers (specific finding highlighted in bold in the report). In **a**, **b** and **e**, data are presented as mean values  ± 95% confidence intervals obtained via bootstrapping (1000 resamples, 2.5th–97.5th percentiles). Statistical significance in **a** and **b** was assessed using a two-sided DeLong test for AUROC and a two-sided paired permutation test (1000 bootstraps) for mDice; no adjustments were made for multiple comparisons. Exact p-values are reported in the main text.

In clinical practice, it is crucial for models to be robust to the demographic and social origins of patients. Thus, we evaluated our classification models on external patients from populations with different profiles. We used either CheXpert or MIMIC as internal training data, both collected in the USA, and tested them on the external BRAX dataset^33^ from Brazil. As depicted in Fig. 4a, RayDINO significantly outperforms all other models on the BRAX dataset when trained on CheXpert (0.783 AUROC 95%CI [0.776–0.790],  + 0.025 above 0.758 of ARK6, *p*≤1e − 4) and on MIMIC (0.789 AUROC 95%CI [0.783–0.796],  + 0.022 above 0.767 of UniChest, *p*≤1e − 4). This demonstrates its robustness to different disease distributions and patient origins. We highlight an interesting observation in Supplementary Fig. 4: despite not being the top performer on the CheXpert dataset, RayDINO generalizes significantly better than all other models on a new population, indicating its ability to avoid bias and overfitting to the training population.

We further evaluate RayDINO’s generalization performance in segmentation tasks, training on CT-scan projections mainly from the USA and China (PAXRay++) and testing on two external datasets from Japan (JSRT) and Vietnam (Vindr Rib), as shown in Fig. 4b. We emphasize the fact that the training and validation data are synthetic 2D chest X-rays made from CT-scan projections while the test data are real chest X-rays. Despite the synthetic nature of the training data and the distinct geographical origins of the patients, RayDINO shows a significant generalization improvement with, respectively, 0.801 and 0.770 mDice in Japanese and Vietnamese patients (95%CI [0.795–0.807] and [0.749–0.788],  + 0.032 and  + 0.185 above 0.769 and 0.585 of CheXzero, *p*≤1e − 3 and *p*≤1e − 3). RayDINO even outperforms the fully supervised CXAS model^34^ trained on PAXRay++ when evaluated on Vinr Rib with  + 0.036 mDice above 0.734. We show an example of how both models segment real world images in Supplementary Fig. 5.

### Examining performance across sex and age groups

Ensuring fairness and reliability is crucial for foundation models that would be used on diverse applications and populations. While we make no definitive claims about our model, we provide an analysis of biases to aid researchers in effectively utilizing our method.

First we evaluated sex-specific bias by following Larrazabal et al.’s setting^35^. We assessed the AUROC difference on a test split comprising only one sex when training on the same sex versus training on the other sex (male or female, intersex and hermaphrodite data being unavailable). We use 20 folds and also included a combined sex split termed ‘All’ as a topline. We report results in Fig. 4c. According to the Mann-Whitney test, we observe that RayDINO exhibits a significant performance gap between male and female when tested on the same sex (*p*≤1e − 4 for all four comparisons). The two other models, ARK6 and UniChest, show a non-significant gap in similar scenarios one time out of four for ARK6 and two times for UniChest, suggesting that self-supervised learning may be more sensitive to sex-specific characteristics.

However, while the sex bias seems to be higher than supervised baselines, RayDINO outperforms by a large margin other models in terms of raw performance on both NIH and CheXpert (respectively  + 0.011 AUROC above 0.842 of ARK6 and  + 0.025 AUROC above 0.724 of UniChest on females  → males with *p*≤0.01 and *p*≤1e − 5,  + 0.018 above 0.814 of UniChest and  + 0.016 above 0.719 of ARK6 on males  → females with *p*≤1e − 5 for both cases). Notably, RayDINO surpasses these models when comparing its worst minority group performance against the best group performances of ARK6 and UniChest ( + 0.008 average AUROC when testing on males and training RayDINO on females above 0.793 of the other best model trained on males, *p*≤0.05, and  + 0.009 above 0.772 when testing on females and training RayDINO on males against the other best model trained on females with *p*≤1e − 5). This indicates that even when the task-specific adapter is trained on biased data, RayDINO can effectively mitigate this bias. Despite a larger performance gap between males and females, RayDINO makes fewer errors on all splits.

To emulate a complex scenario where populations vary in terms of prevalence and visual characteristics, we extended this analysis to patient’s ages. We divided the MIMIC dataset into three age groups: Young/Y (15–35 years), Middle/M (35–60 years), and Elder/E (60–100 years), and sampled them equally. As depicted in Fig. 4d, RayDINO achieves the best performance across all combinations with an average AUROC increase of  + 0.008 above 0.808 of ARK6. It either matches the performance (in the Y → M, Y → E, E → Y combinations with *p* = 0.47, *p* = 0.62 and *p* = 0.08) or consistently outperforms the best alternative model ARK6 in the six other combinations (*p*≤0.05). Further details about split and dataset statistics used for the bias analysis are provided in the Additional Results section and Supplementary Table 2.

### Interpretability

Our model is designed to be inherently interpretable, utilizing a simple two-layer decoder with attention pooling on top of our localized holistic features. This enables the generation of attention maps, visual representations that highlight the specific areas the model focuses on during prediction. Examples of such attention maps, confirmed by expert radiologists from this study, are shown in Fig. 2c for Pneumothorax classification, in Fig. 3e for Scoliosis angle regression and in Fig. 4f for Fractures, Subcutaneous Emphysema, Nodules and Hernia. We show additional images in Supplementary Fig. 6 as well as an example of a identified failure cases thanks to RayDINO’s interpretability in Supplementary Fig. 7. By analyzing these maps, we can accurately pinpoint the location and extent of diseases within the images. Radiologists can also gain insights into the model’s prediction process. For instance, in the scoliosis case, each attention head (represented by different colors) focuses on a different part of the image. Such detailed decomposition of the model’s reasoning process is not possible with traditional methods like GradCAM^36^, which only produce a single, less precise heatmap^37^. We evaluated the pinpointing performance of each model by examining if the area with the highest attention score for each disease was within the radiologists’ bounding boxes on the VinDr dataset (Fig. 4e). RayDINO outperforms all other model in term of localization, achieving a macro accuracy of 0.527% (95%CI [0.448–0.598],  + 0.102pp above 0.425% of UniChest, *p*≤0.05). This level of interpretability not only offers insights into the model’s decision-making process but also aids in validating its predictions, thereby enhancing trust in the model’s outputs.

## Discussion

By using recent techniques for scaling self-supervision based on DINOv2, we developed RayDINO, a state-of-the-art model that can analyze chest X-rays in a holistic manner, trained in a patient-centric way using only the imaging data rather than annotations that follow country-specific guidelines. Our findings indicate that RayDINO’s representation provides state-of-the-art performance on all 21 benchmarks by surpassing the best available chest X-ray foundation models in the litterature on most of them (Fig. 1d), without relying on human annotations during its pre-training phase. In addition, RayDINO handles better rare categories, worst minority groups, and external demographics. Finally, as an additional source of information, our model also provides stronger interpretability, qualitatively validated by expert radiologists and quantitatively using attention pinpointing.

Recently, several models presented in the literature proposed general representations of X-ray imaging using either imaging, or coupling imaging with text, evaluated on different tasks^20–24^. We have proven RayDINO’s superiority over these models on several radiology tasks. In addition, it has been assessed on more holistic and clinically relevant issues compared to other methods for X-ray image processing (REMEDIS^6^, Rad-Dino^38^), showing better adaptation to unseen tasks, with few labels and good localization, staying more robust in new populations and better mitigating bias inherent to the data. To the best of our knowledge, our study is the first to train the largest self-supervised method on 840.000 X-rays and test it across 9 tasks on 21 different benchmarks comprising more than 200 categories in both internal and external settings, from patients of seven countries across four continents, and reaching good interpretability with state-of-the-art results in the process. Previous work focused on populations from one country, few benchmarks, or without completely external validation^6,7,38^. Our extensive evaluation, audit RayDINO’s ability to handle multiple tasks across diverse demographics, providing more insights about performance and limitations of current foundation models.

This study highlights also multiple advances in image-based self-supervised foundation models, providing evidence for their holistic understanding and real clinical usability. One of the first advances of RayDINO compared to other task-oriented models is that it does not make assumptions about specific classes. Instead, RayDINO focus on all discriminative characteristics of the X-ray, capturing the real properties of the patient without guideline-related limitations. This holistic approach allows RayDINO to tackle multiple tasks at once and to reach better performances compared to other models on new diseases that do not exist in the original data such as COVID-19 or abnormalities such as pneumoperitoneum, pneumomediastinum and subcutaneous emphysema, from the MIMIC Long-Tail evaluation, which are clinically rare^39,40^.

Moreover, the general purpose of our model is also highlighted in the report generation task. A radiologist’s report includes multiple pieces of information such as whether a disease is present along with elements supporting that conclusion, as well as other general observations unrelated to the aforementioned disease, e.g. bone-related issues, presence of catheters or pacemakers, and so on. The variety of information included in text reports is difficult to cover with weakly-annotated datasets, justifying the new usage of textual supervision in models such as Refers^20^, CheXzero^21^ and UniChest^23^. Interestingly, RayDINO outperforms all of these models on the report generation task without using text annotations during its pretraining. These findings highlight that image-based models that are trained to explicitly search for discriminative or rare features, thanks to self-supervised learning, can outperform multimodal models when building open-ended medical artificial intelligence^41^.

While defining fairness in medical imaging remains a complex challenge^42,43^, RayDINO has the potential to reduce disparities in access to medical care across diverse populations. Studies have highlighted variations in medical practice within the United States, with clinicians demonstrating differences in the treatment and diagnosis of disorders among patients from different demographics, irrespective of the patients’ actual needs^12–14^. Moreover, diagnostic guidelines can vary between countries, but our model aims to cater to a broader audience beyond the countries it has been trained on. By not relying on human annotations for training, our RayDINO backbone has learned none of the bias inherent in patient-based diagnostic guidelines and radiologists’ interpretations. This also removes errors that can arise in automatic annotation pipelines^16,17,19^, enabling the fairer training of RayDINO and the extraction of holistic features that accurately represent the patient’s condition.

However, such bias can re-emerge during the fine-tuning of the foundation model using annotations. To address this, we evaluated our backbone in a frozen setting, without modifying its parameters, and instead trained very small task-specific adapters. This approach offers three key advantages. Firstly, using the same foundation model for all tasks enhances the ability to audit the raw features of the model without incorporating label and task-specific bias. Secondly, the bias from the annotations is captured only by a simple decoder, reducing overfitting, as demonstrated in our generalization evaluation from the USA to Brazil. Lastly, the inherent interpretability of our adapters allows radiologists to directly verify whether the model is relying on spurious correlations in the X-ray for its predictions as shown in the interpretability section. The consistent and significant improvement of RayDINO on the worst minority groups with respect to sex and age, as well as the good classification transfer on new populations, shows the potential of this model for clinical use and adaptability in regions with limited access to healthcare systems.

### Limitations

Although RayDINO performs better than prior work on our benchmarks, it isn’t devoid of limitations. Firstly, to enhance generalization to external populations, future iterations should incorporate data from a broader range of regions and demographics beyond the USA and Europe and test on external populations other than Brazil, Vietnam and Japan. Secondly, while RayDINO demonstrates improved performance for minority groups and bias mitigation, it exhibits a slightly larger gap between performances stratified by sex. Thirdly, the group-specific bias could be developed to provide more comprehensive results. This could be achieved by exerting more control over the studied population, for instance, through an intersectionality analysis. Finally, the primary objective of this study is to explore the capabilities of our unimodal foundation model, which is solely based on 2D imaging. We aimed to demonstrate its ability to perform a holistic analysis of X-rays, with a particular emphasis on diagnostic tasks. Future work will focus on using this model on prognostic tasks investigating also the importance of patient’s history, symptoms and follow-up as well as 3D CT-scans.

## Methods

### Ethics statement

This research complies with all relevant ethical regulations. This study exclusively uses publicly available, de-identified datasets that were collected and released under their respective institutional review board (IRB) approvals and data use agreements. No original human subjects data were collected for this work; therefore, no additional IRB approval or informed consent was required. All datasets used are fully anonymised and do not contain personally identifiable information. Details of each dataset, including the country of origin, are provided in Table 2.

**Table 2:** Overview of all the datasets used in this study

Name	Used For	Train	Val	Test	Country	Type	Labels	# Label	% Female	Avg. Age	% Healthy
*Pretraining Datasets*											
PadChest	Pretrain	160.845	-	-	Spain	-	-	-	49.6	58.6	31.4
CheXpert	Pre. & Int.	223.414	234	668	USA	Classif.	Findings	14	40.7	60.6	10.0
MIMIC	Pre. & Int.	368.960	2.991	5.159	USA	Classif.	Findings	14	47.5	61.6	38.3
NIH	Pre. & Int.	83.524	3.000	25.596	USA	Classif.	Findings	15	44.0	46.6	58.4
*Internal Evaluation Datasets*											
VinDr	Internal	14.000	1.000	3.000	Vietnam	Classif.	Findings	28	-	-	70.7
PAXRay++	Internal	11.802	1.475	1.476	Multiple	Segm.	Bones & Organs	159	-	-	-
Scoliosis	Internal	481	-	128	Canada	Reg.	Spine angles	3	-	-	-
Pneumothorax	Internal	10.675	1.372	3.205	USA	Segm.	Diseases	2	-	-	77.7
POLCOVID	Internal	2.985	509	1.315	Poland	Classif.	Diseases	3	47.4	60.2	65.8
Catheter	Internal	24.066	3.008	3.009	USA	Classif.	Catheter Pos.	9	-	-	-
MIMIC-LongTail	Internal	264.849	36.769	75.492	USA	Classif.	Findings	40	47.5	61.6	38.3
Report Generation	Internal	267.215	2.114	3.822	USA	Text Gen.	Radiological Reports	-	48.6	61.3	46.4
*External Evaluation Datasets*											
JSRT	Int. & Ext.	197	-	50	Japan	Segm.	Organs	5	52.8	58.2	-
VinDr Rib	Int. & Ext.	196	-	49	Vietnam	Segm.	Ribs	20	-	-	-
BRAX	External	-	-	40.967	Brasil	Classif.	Findings	14	50.6	46.0	70.8

They are grouped in 3 categories: pre-training of RayDINO, internal evaluation set (downstream adapter trained on them), or external evaluation set (adapter not trained on them). One dataset can be used in multiple setups. The number of exams used for the training, validation and test splits for each dataset is presented together with information about the country of origin, the type of benchmark and labels as well as the % of female and healthy exams and the average age.

Sex information was available for CheXpert, MIMIC, NIH, POLCOVID, JSRT and BRAX datasets (see Table 2); it was recorded as a binary variable (male/female) based on the original dataset annotations. Age was available for CheXpert, MIMIC, NIH, POLCOVID, JSRT and BRAX. Sex and gender were not considered in the study design for the pretraining phase, as no annotations were used. For the bias analysis experiments (Fig. 4c-d), sex and age were explicitly used as stratification variables to evaluate model fairness. We acknowledge that the binary sex categorisation does not capture the full spectrum of sex and gender identities, and that intersex and non-binary data were unavailable in the datasets used.

All experiments were repeated 5 times with different random seeds (or 20 folds for the bias analysis), and all attempts at replication were successful. Due to the retrospective nature of this study using pre-existing anonymised datasets, blinding of investigators to group allocation was not applicable.

### Unsupervised pretraining

Throughout this work, we use Vision Transformers^15^ (ViTs). These models, when given a new image, produce a global representation, as well as a feature map describing individual patches of 16 × 16 pixels in the context of the image. For a 224 × 224-pixel image, the model provides one global representation token [CLS], and a 14 × 14 map of patch-level representation tokens. We train our RayDINO models using an unsupervised feature learning method, adapting the training algorithm of DINOv2^3^. The training procedure proposed in that work uses three different loss functions: (i) the DINO loss, first proposed in DINO^44^, encourages different data augmentation views (e.g. crops, geometric and colorimetric distortions) of a given image to lead to the same [CLS] token. (ii) a regularization term, KoLeo^45^, ensuring better coverage of the representation space by pushing the [CLS] tokens of different images away from each other. (iii) the iBOT loss, first proposed in iBOT^46^, allows learning better local representations through masked image modeling: during training, the model learns to predict a hidden part of its input, based on the surrounding context.

Following previous work, we train the biggest model currently available for radiology based on the ViT architecture with 307M parameters coined ViT-L (Large), producing tokens of dimensionality 1024. We provide an ablation of the performance as a function of model size in Supplementary Fig. 1, showing in particular that performance increases with model size. We train the model for 125k iterations with a minibatch size of 2048. The learning rate follows a standard cosine schedule with 12.5k iterations of linear warmup to a peak value of 1.10^−3^. The weight decay follows a cosine schedule from 0.04 to 0.4. We point out that these are the default hyperparameters of the “Fast Setup" setting in the open-source DINOv2 implementation (https://github.com/facebookresearch/dinov2). We deviate from the default settings only for the image crops due to the higher level of detail of XRay images: we employ 512 × 512-pixel crops (instead of 224 × 224) for the large crops, 224 × 224 (instead of 96 × 96) for the small crops; additionally, the large crops must cover at least 15% of the image (instead of 32%) while the small crops cover at most 15% of the image (instead of 32%). Training is performed on 128 A100-80GB Nvidia GPUs and lasts 36 hours.

### Data

The original DINOv2 model was trained on a dataset composed of 142M images, curated from a large pool of internet images. However, in the context of X-ray image analysis, we don’t have access to such large web-sized data pools: therefore, we combine four publicly available datasets (CheXpert^17^, MIMIC^16^, NIH-14^18^, PadChest^19^) to train the RayDINO models. This sums up to approximately 840k images used for our unsupervised pretraining; in contrast to supervised approaches, we do not employ any annotations; therefore, it is possible to increase the size of the pretraining dataset easily by simply collecting more radiographs or including more suitable datasets.

Our evaluation datasets include MIMIC, NIH, CheXpert, and SIIM-ACR Pneumothorax^47^ from the USA; VinDr-CXR^48^ and Vindr-RibCXR^49^ from Vietnam; POLCOVID^30^ from Poland; BRAX^33^ from Brazil; JSRT^50^ from Japan; AASCE2019 Scoliosis^31^ from Canada; and PAXRay++^34,51^, which consists of synthetic 2D projections of CT-scans primarily from the USA and China. We also improve the granularity of the MIMIC and NIH annotations by incorporating new annotations from RANZCR CLiP Catheter^28^ and MIMIC Long-Tail^27^. All these datasets are publicly available, and for our experiments, we used the official training, validation, and test splits whenever available. Their repartition to pretraining of RayDINO, their distributions, and their available annotations are summarized in Table 2 and 3. We use train, validation, and test splits for all evaluations. To make our results reproducible we used all official test splits when available. When no such official splits are available, we used respectively 80%, 10%, and 10% of the data for the train, validation, and test sets. For all the dataset, we applied the same preprocessing including:

- In case of raw dicom files, we use a Look-Up Table (LUT) to convert pixels to the [0 − 255] range using a window width of (max − min) and a window level of (max + min)/2.
- All exams were resized to 512 × 512 pixels and transformed to three channels by replicating the channel provided.
- All exams were normalized using classical ImageNet parameters of mean = (0.485, 0.456, 0.406) and std = (0.229, 0.224, 0.225).

**Fig. 5:**
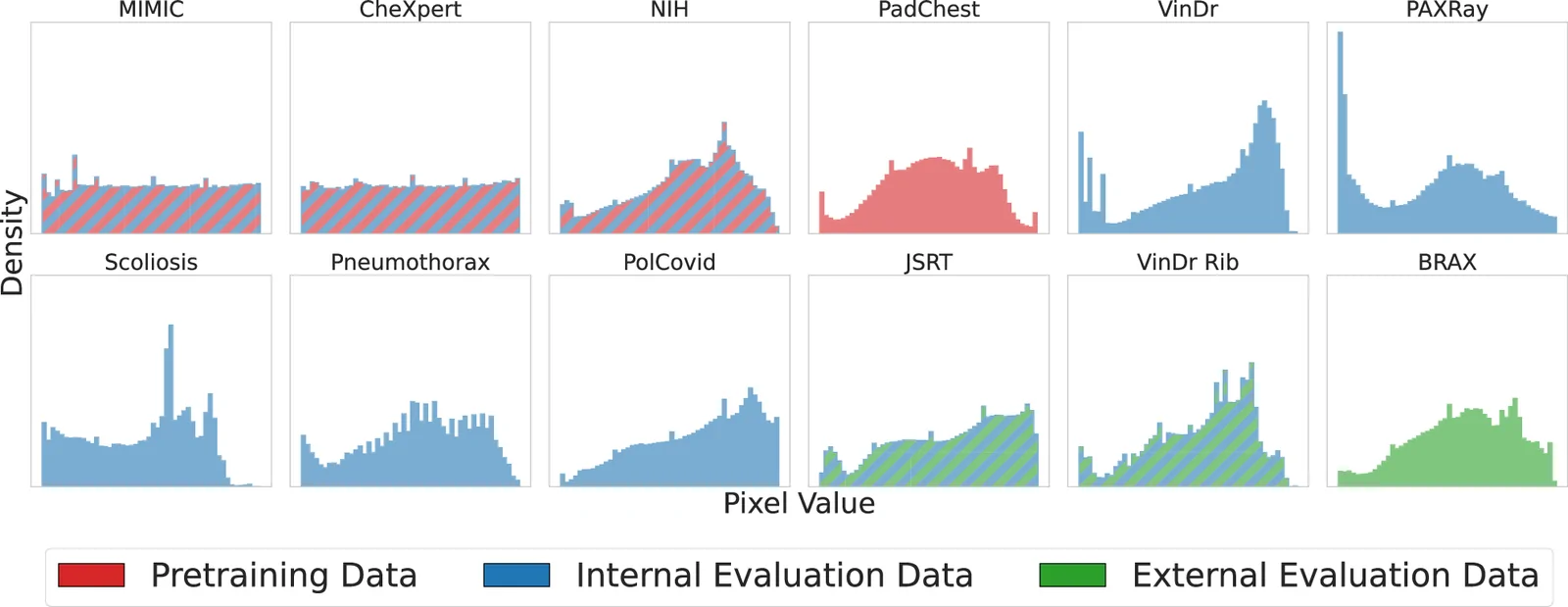
Non-zero pixel values histogram for each dataset in this study. For each histogram we indicate with different colors their use to pretraining, internal and external evaluation datasets.

To highlight the differences of these datasets, we present the overall pixel-value distributions for each of them before normalization in Fig. 5. One can observe that while all datasets use the full 0-255 range, most of them have very different distributions, showcasing the difficulty to generalize from eg. MIMIC to BRAX or from PAXRay to JSRT and VinDr Rib.

**Table 3:** Overview of the different datasets and their detailed classes used in our study

CheXpert	Atelectasis, Cardiomegaly, Consolidation, Edema, Enlarged Cardiomediastinum, Fracture, Lung Lesion, Lung Opacity, No Finding, Pleural Effusion, Pleural Other, Pneumonia, Pneumothorax, Support Devices
MIMIC	Atelectasis, Cardiomegaly, Consolidation, Edema, Enlarged Cardiomediastinum, Fracture, Lung Lesion, Lung Opacity, No Finding, Pleural Effusion, Pleural Other, Pneumonia, Pneumothorax, Support Devices
NIH	Atelectasis, Cardiomegaly, Consolidation, Edema, Effusion, Emphysema, Fibrosis, Hernia, Infiltration, Mass, No Finding, Nodule, Pleural Thickening, Pneumonia, Pneumothorax
VinDr	Aortic Enlargement, Atelectasis, Calcification, Cardiomegaly, Clavicle Fracture, Consolidation, Copd, Edema, Emphysema, Enlarged Pa, Ild, Infiltration, Lung Cavity, Lung Cyst, Lung Opacity, Lung Tumor, Mediastinal Shift, No Finding, Nodule Mass, Other Disease, Other Lesion, Pleural Effusion, Pleural Thickening, Pneumonia, Pneumothorax, Pulmonary Fibrosis, Rib Fracture, Tuberculosis
PaxRAY++	Spine, Cervical Spine, Thoracic Spine, Lumbar Spine, Vertebrae C1, C2, C3, C4, C5, C6, C7, Vertebrae T1, T2, T3, T4, T5, T6, T7, T8, T9, T10, T11, T12, Vertebrae L1, L2, L3, L4, L5, Rib Cartilage, Sternum, Clavicles, Clavicle Left, Clavicle Right, Scapulas, Scapula Left, Scapula Right, Posterior Rib R1, R2, R3, R4, R5, R6, R7, R8, R9, R10, R11, R12, Anterior Rib R1, R2, R3, R4, R5, R6, R7, R8, R9, R10, R11, Posterior Rib L1, L2, L3, L4, L5, L6, L7, L8, L9, L10, L11, L12, Anterior Rib L1, L2, L3, L4, L5, L6, L7, L8, L9, L10, L11, Diaphragm, Left Hemidiaphragm, Right Hemidiaphragm, Stomach, Small Bowel, Duodenum, Liver, Pancreas, Kidney Left, Kidney Right, Cardiomediastinum, Upper Mediastinum, Lower Mediastinum, Anterior Mediastinum, Middle Mediastinum, Posterior Mediastinum, Heart, Heart Atrium Left, Heart Atrium Right, Heart Myocardium, Heart Ventricle Left, Heart Ventricle Right, Aorta, Ascending Aorta, Descending Aorta, Aortic Arch, Pulmonary Artery, Inferior Vena Cava, Esophagus, Lung, Right Lung, Left Lung, Lung Base, Mid Zone Lung, Upper Zone Lung, Apical Zone Lung, Right Upper Zone Lung, Right Mid Zone Lung, Right Lung Base, Right Apical Zone Lung, Left Upper Zone Lung, Left Mid Zone Lung, Left Lung Base, Left Apical Zone Lung, Lung Lower Lobe Left, Lung Upper Lobe Left, Lung Lower Lobe Right, Lung Middle Lobe Right, Lung Upper Lobe Right, Trachea, Tracheal Bifurcation, Breast, Breast Left, Breast Right
Scoliosis	MT Angle, PT Angle, TL-L Angle
Pneumothorax	Normal, Pneumothorax
POLCOVID	Covid, Normal, Pneumonia
JSRT	Left Lung, Right Lung, Left Clavicle, Right Clavicle, Heart
VinDr Rib	Rib R1, R2, R3, R4, R5, R6, R7, R8, R9, R10, L1, L2, L3, L4, L5, L6, L7, L8, L9, L10
BRAX	Atelectasis, Cardiomegaly, Consolidation, Edema, Enlarged Cardiomediastinum, Fracture, Lung Lesion, Lung Opacity, No Finding, Pleural Effusion, Pleural Other, Pneumonia, Pneumothorax, Support Devices
Catheter	Ett Abnormal, Ett Borderline, Ett Normal, Ngt Abnormal, Ngt Borderline, Ngt Normal, Cvc Abnormal, Cvc Borderline, Cvc Normal
MIMIC-LT	Adenopathy, Atelectasis, Azygos Lobe, Calcification Of The Aorta, Cardiomegaly, Clavicle Fracture, Consolidation, Edema, Emphysema, Enlarged Cardiomediastinum, Fibrosis, Fissure, Fracture, Granuloma, Hernia, Hydropneumothorax, Infarction, Infiltration, Kyphosis, Lobar Atelectasis, Lung Lesion, Lung Opacity, Mass, Nodule, Normal, Pleural Effusion, Pleural Other, Pleural Thickening, Pneumomediastinum, Pneumonia, Pneumoperitoneum, Pneumothorax, Pulmonary Embolism, Pulmonary Hypertension, Rib Fracture, Rounded Atelectasis, Subcutaneous Emphysema, Support Devices, Tortuous Aorta, Tuberculosis

The table presents a clear list of all the benchmarked X-ray anomalies and anatomies, showing the extensive evaluation of RayDINO.

### Comparisons and baselines

In all our evaluations, we compare our model to several publicly available X-ray foundation models. We select five recent methods, representative of the current state of the art, as comparison baselines. ref.^20^ is a model trained using cross-supervision between radiology images and text from radiology reports. CheXzero^21^ employs contrastive language-image pretraining (akin to CLIP^9^) to produce a vision model able to tackle Chest X-ray images. KAD^22^ is similarly trained using paired radiology images and reports, with the addition of a knowledge-base component to improve performance. Inference is then performed following the CLIP zero-shot protocol, effectively performing classification over the list of extracted disease names. UNIChest^23^ consists of an improved model built on top of the KAD method. Finally, ARK6^24^ is a foundation model trained on a concatenation of multiple existing datasets used in a classification training pipeline, effectively using a large amount of categorical supervision. Regarding training data, the models against which we show comparisons are trained on a relatively similar set of datasets as our model. REFERS, CheXzero, and KAD are trained on the MIMIC dataset^16^. UniChest additionally leverages NIH^18^, CheXpert^17^, and Vindr^48^. ARK6 uses all these datasets along with the data from the RSNA Pneumonia Detection Challenge (https://www.kaggle.com/c/rsna-pneumonia-detection-challenge/) and the Shenzhen dataset^52^. An overview of the different models used for the comparison of RayDINO together with information about the task-specific adapters used for each of the benchmarks is presented in Table 1.

Recent concurrent work RAD-DINO^38^ explores a direction similar to our approach by building on DINOv2 models. They initialize a vision model with a DINOv2 checkpoint, using a smaller model ViT-B of 86M parameters, then perform training for a small number of iterations (7 times less than RayDINO) using a combination of datasets and compare favorably against smaller models found in the literature. They find that text supervision is not necessary to build strong vision models for the medical domain, that SSL features present viable properties for medical applications, and that it should be possible to build a strong foundational image encoder with an SSL approach. In this study, our model RayDINO successfully tests that hypothesis, leading to a versatile model that outperforms all prior state-of-the-art approaches on a multitude of downstream tasks. We compare our work against the strongest model available in the literature on a wider variety of tasks including very important ones like external validation on new demographics and age and sex-related bias analysis.

### Experimental protocols

#### Classification

All the tasks considered in this part for the evaluation take as input the whole image and have a simple output space (classification or regression). As mentioned before, for all models, we keep the parameters of the image encoder frozen and learn a simple decoder on top using stochastic gradient descent. Thanks to the use of very small decoders, we can use the validation set to select the best decoder through a grid search over the following hyperparameters: learning rate: 13 values: [1.10^−5^, 2.10^−5^, 5.10^−5^, 1.10^−4^, 2.10^−4^, 5.10^−4^, 1.10^−3^, 2.10^−3^, 5.10^−3^, 1.10^−2^, 2.10^−2^, 5.10^−2^, 1.10^−1^]; weight decay: 3 values: [0, 1.10^−5^, 1.10^−4^]; number of layers in decoder: 1 (linear) or 2 (MLP); feature pooling: attentive pooling using *n*_*classes*_ heads or average pooling. When using MLPs, we set the hidden dimension to 64 and use ReLU as the activation function. In total, the grid contains 156 hyperparameter combinations that we execute at a minimal cost by performing only a single inference of the image encoder and training decoders simultaneously. We use a binary cross-entropy loss for multi-label classification tasks (CheXpert^17^, MIMIC^16^, NIH^18^), a simple cross-entropy loss for POLCOVID (as it is not multi-label), and the SMAPE loss for Scoliosis regression.

#### Segmentation

We evaluate all models on 4 datasets: PAXRay++^34,51^, JSRT^50^, Vindr Rib^49^ and SIIM Pneumothorax^47^. We compare the models using the macro averaged Dice score computed per class. We use the official sets from Vindr Rib, take 80/10/10% splits for PAXRay and 80/20% splits for JSRT by validating on the train set, and use the stage 2 validation data from Siim Pneumothorax as test set. All images are rescaled to 512 × 512 pixels for RayDINO and the nearest smaller multiple of the patch_size for other models and metrics are computed at 512 × 512 pixels. All backbones are frozen and we train conjointly a UperNet head and a FCN head with a feature pyramid network on top of the extracted features using a Dice loss. We train for 5000 iterations using a batch size of 16 and run a grid search over 9 learning rates [1.10^−5^, 5.10^−4^, 1.10^−4^, 1.10^−3^, 3.10^−3^, 1.10^−2^, 3.10^−2^, 1.10^−1^, 3.10^−1^] with a linear schedule (1500 warmup iterations, then linear decay until 0).

#### Generalization

Models are evaluated on two generalization tasks. For the population generalization tasks we select the classification heads trained and validated on CheXpert^17^ (USA, 224k images) or MIMIC^16^ (USA, 377k images), and evaluate them on the whole BRAX^33^ dataset (Brazil, 41k images), reporting AUROC. For the population and CT-scan projection generalization we train and validate segmentation heads on PAXRay++^34,51^ (multiple countries, mainly China and the USA, 15k images) and test them on JSRT^50^ (Japan, 154 images) and Vindr Rib^49^ (Vietnam, 245 images) by mapping labels accordingly and reporting mDice score.

#### Report generation

We finetune a publicly available large language model, namely the LLama2-7B model^53^ to perform report generation. We first convert image features, using a small two-layer Transformer adapter, into tokens of the dimensionality expected by the language model. Then, this language model can be trained to produce reports directly from the image tokens. To this end, we employ an image captioning loss to generate the findings section of the MIMIC^16^ reports, using the indication section as prompt when available. We train for 10.000 iterations with a batch size of 8 sequences of 2048 tokens. For a fair comparison, we align on the split and the text preprocessing used in MAIRA-1^26^, and refer to their manuscript for more details.

#### Bias analysis

To perform the bias analysis of our RayDINO backbone, we train classification heads using only subsets of the same size from the training data corresponding to a category of interest, e.g. the female subset in NIH^18^. Then, we evaluate the resulting model on the other categories to assess the presence of bias, and report the AUROC metric. We also train a classification head on the whole training data of all subsets to observe the performance differences with respect to represented categories. For each task and data subset in the bias analysis, we follow the protocol for classification evaluation described further above. We repeat this on 20 different folds and report the results of a Mann-Whitney test to look for significant differences.

#### Interpretability

We train a disease-specific classification adapter for each model using a single attention head on the VinDr training set. We then generate attention maps for each test image in the VinDr dataset. To calculate the final accuracy result for each disease, we examine all true disease bounding boxes and check if the patch with the highest attention score from the specific disease’s adapter intersects with the bounding box by at least half the patch’s area. We then compute the macro average of accuracies for all diseases and report the result.

### Evaluation metrics

Throughout this work, we employ several evaluation metrics to compare our model to the state of the art in the literature, and we describe these here for the case of image-related tasks. For multilabel binary classification, we employ the Area Under the Receiver-Operator Curve (AUROC) metric, and report the macro average over classes. For long-tail binary classification, we employ the Area Under the Precision-Recall Curve (AUPRC), as this metric handles unbalanced classes better, and we report the macro averaged AUPRC over classes, when applicable. For simple classification (where classes are mutually exclusive, as in POLCOVID^30^), we report the accuracy of the classifier averaged over classes (Macro Accuracy). For segmentation tasks, we use the Dice metric and report the average over classes (mDice); this metric indicates the quality of overlap between predicted and ground truth segmentation masks. For regression tasks (e.g. scoliosis angle), we report the correlation value between the prediction and the ground truth.

For evaluating text outputs, as done for the report generation task, the performance measurement is more complex. In order to compare with alternatives, we report several metrics used in the literature, namely BLEU-4^54^, ROUGE-L^55^, RadGraph F1, CheXbert vector, RadCliQ, and Macro-F1-14. BLEU-4 and ROUGE-L are metrics designed for the evaluation of translation models and allow measuring the quality of generated text with respect to a reference text. In the case of report generation, they are approximate metrics that we employ as proxies for reference, using the pycocoevalcap library. For proper comparison with the MAIRA-1 work, proposing a radiology report generation pipeline, we also compute the same metrics as their authors and refer to their manuscript for additional details. RadGraph-F1 is a metric based on the RadGraph model, which parses generated and ground truth radiology reports into graphs containing clinical entities and relations between them, then computes the overlap which is averaged into a metric. RadCliQ combines RadGraph-F1 and BLEU. Similarly, the CheXbert vector metric compares a generated and reference report using the CheXbert model. CheXpert F1 metrics (referred to as Macro-F1-14 and Micro-F1-14 in the results) use the CheXbert automatic labeller to produce a metric based on the precision and recall over the 14 radiology observations. We used the reference implementation provided with the original work introducing the metrics^56^.

### Statistical analysis

When comparing the performance of two models, to verify that the difference between their results is significant, we use the DeLong^25^ test when computing AUROC. Moreover, we also report a bootstrapped paired permutation test for other metrics (1000 bootstraps and 2.5 − 97.5 percentiles). We report p-values for all our statistical tests, with low p-value meaning that the difference in performance is statistically significant. In the bias analysis, we train populations of models (using 20 different cross-validation folds) using labels from different groups considered (e.g. age, sex) and obtain classification accuracy scores; we then employ the Mann-Whitney test to measure, by sampling X and Y from the two groups, that the probability of *X* > *Y* is equal to the probability of *Y* > *X*. A high value suggests that populations perform equally.

We can see the results of this analysis in table 4, where we computed per task and per dataset the number of classes with RayDINO being either significantly superior, inferior, or non-significantly different form the best baseline model.

**Table 4:** Statistical comparison of RayDINO and the best other model for each task, dataset and class

			Num. classes with better performances			
Task	Dataset	Num. Class.	RayDINO (*p* < 0.05)	NS (*p*≥0.05)	Other Best Model (*p* < 0.05)	Other Best Model
Classification	MIMIC	14	5	9	0	UniChest
	NIH	15	8	7	0	ARK6
	VinDr	27	3	18	6	UniChest
	CheXpert	14	1	12	1	UniChest
	Total	70	17	46	7	-
Rare Class Prediction	MIMIC Long-Tail	26	22	4	0	UniChest
	Catheter Malposition	9	8	1	0	ARK6
	Total	35	30	5	0	-
Segmentation	JSRT	5	5	0	0	CheXzero
	PAXRay	159	152	7	0	CheXzero
	Pneumothorax	1	1	0	0	UniChest
	VinDr Rib	20	20	0	0	Refers
	Total	185	178	7	0	-
Generalization Classification	CheXpert → BRAX	14	8	5	1	ARK6
	MIMIC → BRAX	14	8	6	0	UniChest
	Total	28	16	11	1	-
Generalization Segmentation	PAXRAY → JSRT	5	3	2	0	CheXzero
	PAXRAY → VinDr Rib	20	20	0	0	CheXzero
	Total	25	23	2	0	-
Generalization Task & Disease	Scoliosis	3	3	0	0	ARK6
	PolCovid	3	2	1	0	ARK6
	Total	6	5	1	0	-
Attention Pinpointing	VinDr	20	5	14	1	UniChest
	Total	20	5	14	1	-
Total		369	274	86	9	-

We use a DeLong test for AUROC comparisons and a paired permutation test for all other metrics.

### Software

All models were implemented in Python (v3.10) using PyTorch (v2.1). Self-supervised pretraining used the open-source DINOv2 codebase (https://github.com/facebookresearch/dinov2). Segmentation heads were implemented using mmsegmentation (v1.2). Report generation used the Llama-2-7B language model^53^. Evaluation metrics for report generation were computed using pycocoevalcap (v1.2), RadGraph^57^, CheXbert^58^, and the RadCliQ reference implementation^56^. Statistical tests were performed using scipy (v1.11). Data preprocessing and analysis used NumPy (v1.24) and pandas (v2.0). All software versions should be verified against the released code repository.

### Reporting summary

Further information on research design is available in the Nature Portfolio Reporting Summary linked to this article.

## Supplementary information

Supplementary Information

Reporting Summary

Transparent Peer Review file

## Source data

Source Data

## Data Availability

All data used in this study are publicly available. CheXpert is available at https://stanfordmlgroup.github.io/competitions/chexpert/. MIMIC-CXR is available at https://physionet.org/content/mimic-cxr/(requires credentialed access via PhysioNet). NIH ChestX-ray14 is available at https://nihcc.app.box.com/v/ChestXray-NIHCC. PadChest is available at https://bimcv.cipf.es/bimcv-projects/padchest/. VinDr-CXR is available at https://physionet.org/content/vindr-cxr/and VinDr-RibCXR is available at https://vindr.ai/ribcxr. BRAX is available at https://physionet.org/content/brax/. JSRT is available at http://db.jsrt.or.jp/eng.php. SIIM-ACR Pneumothorax is available at https://www.kaggle.com/c/siim-acr-pneumothorax-segmentation. PAXRay++ is available at https://www.kaggle.com/datasets/constantinseibold/anatomy-in-chest-X-rays-paX-ray. POLCOVID is available at https://doi.org/10.7303/syn50877085. AASCE2019 Scoliosis is available at https://aasce19.github.io/. RANZCR CLiP Catheter is available at https://www.kaggle.com/c/ranzcr-clip-catheter-line-classification. MIMIC Long-Tail labels are available at https://physionet.org/content/cxr-lt-iccv-workshop-cvamd. Source data are provided with this paper.

## Code availability

Training code is available at https://github.com/facebookresearch/dinov2. The RayDINO model weights are available at https://ai.meta.com/resources/models-and-libraries/raydino-downloads/. The RayDINO model weights are available under the X-ray DINO Research License, which permits use, modification, and distribution strictly for non-commercial research purposes. RayDINO is not an official Meta product.
