# Supplementary material for: Advancing human-centric AI for robust X-ray analysis through holistic self-supervised learning
Source: Nat Commun. 2026 Aug 20;17:9931. doi: 10.1038/s41467-026-76076-4 (PMC13586212; doi:10.1038/s41467-026-76076-4)
Supplement: Supplementary file 1 [file 41467_2026_76076_MOESM1_ESM.pdf]

Advancing human-centric AI for robust X-ray analysis through holistic self-supervised learning

Supplementary Results

We provide additional experimental results and details that complement the analysis presented in the Results Section of the main text. We show additional metrics and comparison points for report generation, statistics for the data used in the fairness analysis, an analysis of the influence of model scaling and data source, and additional analysis for the POLCOVID Classification, spine curvature estimation and Robustness to Patient Demographics probes. We also add more visualizations of RayDINO's interpretability and a comparison of segmentation generalization from 2D CT-scan projection to real-world X-rays between RayDINO and a supervised segmentation model on PAXRay++ called CXAS[34].

Report generation

We present in Supplementary Table 1 the results of the evaluation on the generation of reports for all the competing models considered, including the strong recent state-of-the-art MAIRA-1 model, specifically designed for this task; we provide 95% confidence intervals on performance metrics for better comparison. We observe that RayDINO performs on par or signifiantly better than all competitor models on all metrics. In particular, RayDINO significantly outperforms MAIRA-1 on radiologist-aligned metrics (RadGraph F1, CheXbert score, RadCliQ). We note that, interestingly, RayDINO performs on par with the strongly-engineered GPT4-augmented version MAIRA-1† in all metrics, while relying only on the MIMIC-CXR reports.

Scaling model and dataset size

We perform an ablation study of different model architecture sizes to assess the importance of model size in achieving strong performance. We test our approach on the standard ViT-S (21M params), ViT-B (86M params) and ViT-L (307M params) architectures and present the results in Supplementary Fig. 1 **(top)**. For clarity and brevity, we aggregate tasks in groups. For each group of tasks we report the performance of best competitor model. Unsurprisingly, we observe that the performance significantly increases with model size, indicating a healthy scaling trend. More interestingly, the smaller models match or compare favorably with the best competitor model for all task groups, confirming the superior performance and versatility of the RayDINO pipeline regardless of the model size.

In a second ablation, we look at the influence of the data source we use to train our models. RayDINO is trained on a combined dataset of images from both the USA and Europe, but to evaluate the impact of a diverse set of populations during the pretraining, we train a similar model exclusively on images from the USA. Interestingly, we show in Supplementary Fig. 1 **(bottom)** that the models maintain consistent performance on traditional classification and segmentation tasks regardless of if the training dataset contains images only from the USA or if we also add European data (-0.4 AUROC, $p \leq 0.05$ and $+1.2$ mDice, $p = 0.74$ when we remove European data). This observation suggests that these conventional tasks may not fully capture the models' capabilities or their ability to generalize. However, the inclusion of a more diverse dataset in the training process significantly enhances the model's ability to generalize across different populations in classification ($+0.8$ AUROC, $p \leq$ 1e-5), in segmentation ($+1.9$ mDice, $p \leq 0.01$) and to more accurately predict rare classes ($+0.9$ AUPRC, $p \leq$ 1e-5), thereby boosting its overall performance and applicability in real-world clinical scenarios. While it is systematically better or similar to include European data from PadChest, we point out that the absolute performance difference is of the order of one percent and the strong performances of our model is not only due to the additional data used.

Detailed Analysis for Out-of-Domain Task Performance

We study more in detail the results obtained on out-of-domain tasks in Supplementary Fig. 2 and Supplementary Fig. 3. On POLCOVID, the confusion matrix shows that the model predicts very few (0.6%) false negatives on this dataset when considering the ill/healthy classification task. In a medical context, we want to avoid these type-II errors as much as possible, to make sure ill patients are treated. On the Scoliosis angle regression task, we observe that the model predictions are much more precise for the Main Thoracic curve Cobb angle (MT: $r^2 = 0.90$) than for the other angles (PT: $r^2 = 0.50$ and TL/L: $r^2 = 0.72$).

Statistics of the bias analysis dataset

In Supplementary Table 2, we present statistics on the dataset splits used for our bias analysis. We observe that there is a significant disparity in terms of patients with findings based on sex and age splits. For example, males have 9% fewer findings than females in NIH and 11.8% fewer findings in CheXpert, making these splits easier to predict. Similarly, younger patients have significantly fewer findings compared to the Middle and Elder splits (-13.2% and -34.2%, respectively). This disparity may be due to the data sampling process and may not reflect actual clinical prevalence. It could also introduce bias in

models trained on one split and tested on another, even if the model itself is not biased towards a particular sex or age group. Therefore, it is important to carefully consider the data sampling strategy during the generation of splits to ensure equal odds of finding-specific outcomes.

Additional analysis of generalization between populations

We have shown that our model get excellent robustness when applied to different patient demographics. The observation supporting this claim is the strong generalization of decoders trained on CheXpert or MIMIC when transferred to BRAX. In Supplementary Fig. 4, we show the performance on the transfer dataset (BRAX) as a function of performance on the source dataset (CheXpert or MIMIC). We plot performance for our model and all competing backbones. Each dot represents one trial, and we draw the two-dimensional confidence interval. We also represent the linear regression that we fit on these data points. Models above the plot's linear fit exhibit stronger generalization performance. First, we see that RayDINO exhibits much better generalization, with a significant gap with the linear fit, especially when transferring from CheXpert. Second, while our model achieves the best performance on BRAX, its performance on the source CheXpert data is below that of UniChest, indicating strong overfitting of the competing method on the CheXpert dataset.

It's also noteworthy that all competitor models have been trained in a supervised manner using MIMIC labels. When RayDINO's classifier is trained using labels from MIMIC instead of CheXpert but still tested on CheXpert, it achieves a performance of 89.4 AUROC (95%CI [89.2-89.6]), comparable to the best competitor (-0.02, $p = 0.85$). This suggests that MIMIC may contain higher quality labels than CheXpert and that the comparison in Fig 2a and Supplementary Fig. 4 against ARK6 and UniChest on CheXpert is skewed in their favor.

Generalization results from 2D CT-scan projection to real-world X-rays.

To supplement the quantitative analysis presented in the Results section, we display in Supplementary Fig. 5 real-world X-rays from the NIH dataset. These images are segmented using RayDINO's adapter, trained on PAXRay++ and CXAS[34], a supervised segmentation model also trained on PAXRay++. RayDINO demonstrates the ability to handle more complex images with changes in patient's position and large opacities due to diseases, compared to the supervised model. This is achieved through the use of RayDINO's frozen features, which are trained on real-world X-rays, preventing overfitting on synthetic 2D projections of CT-scans and ensuring familiarity with real-world X-ray distributions.

RayDINO's ability to effectively transfer from 2D CT-scan projections to real chest X-rays is promising. This generalization could pave the way for labeling difficult diseases where practitioners annotate CT-scans as a gold standard and project the labels in 2D. RayDINO could then learn from these synthetic 2D data and transfer the knowledge to real-world X-rays. This approach could potentially reduce annotation errors and, ultimately, improve X-ray diagnostic performance while lowering the cost barrier for medical access.

Additional results for the interpretability of RayDINO.

We show additional interpretability images in Supplementary Fig. 6. We can see that RayDINO can accurately pinpoint findings from all kind of categories on frontal and lateral X-rays. As an example of how RayDINO's interpretability can be harnessed in clinical settings, we show in Supplementary Fig. 7 a RayDINO's classification failure case where the model correctly predicts a fracture but using a wrong spurious correlation. We can see that RayDINO is looking in the top blue rectangle at a neck brace (white arrow) to predict a fracture and miss the badly imaged clavicle fracture in the lower green rectangle (black arrow). Neck braces probably appear more on patients with fractures and thus the model associated such information with the class 'fracture'. Explainable artificial intelligence such as RayDINO can help radiologist and researchers find such spurious correlation and train better classifier using corrected training sets while making more informed analysis of X-rays.

Additional comparisons between RayDINO and other models.

We show per-task and per-dataset individual class comparisons between RayDINO and the baseline models in Supplementary Figs. $8-31$.

Additional statistical tests between RayDINO and other models.

In Supplementary Figs. $32-37$, We show the comparison of RayDINO and other models with different random seeds, in order to assess the consistency and robustness of observed performance differences. Given the sensitivity of representation learning to initialization and data sampling, we believe it is meaningful to assess consistency over multiple seeds and report statistical comparisons across them. We use a t-test across the 5 seeds of each models to compare them. We can see that all models show very low variability, and that RayDINO's performances are statistically significant across the range of all evaluations.

Supplementary Figures

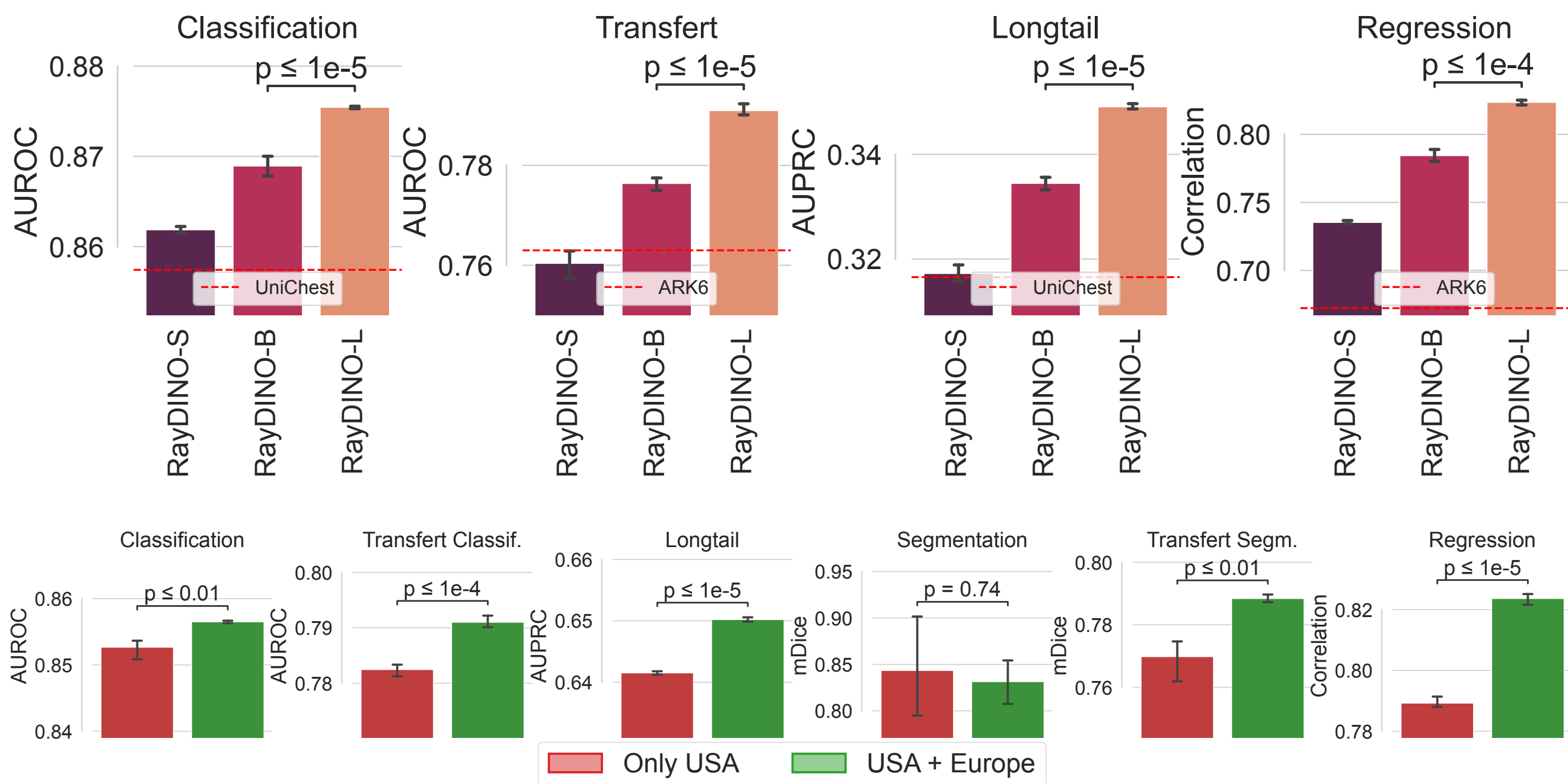

Supplementary Fig. 1. **(top)** Scaling Curve: we compare the impact of model's size on the aggregated results for multiple benchmarks. We report the best competitor model with a red dashed line as a reference. **(bottom)** Ablation on Populations from Training Data: we compare the impact of using patients from the USA only versus the USA and Europe when pretraining RayDINO.

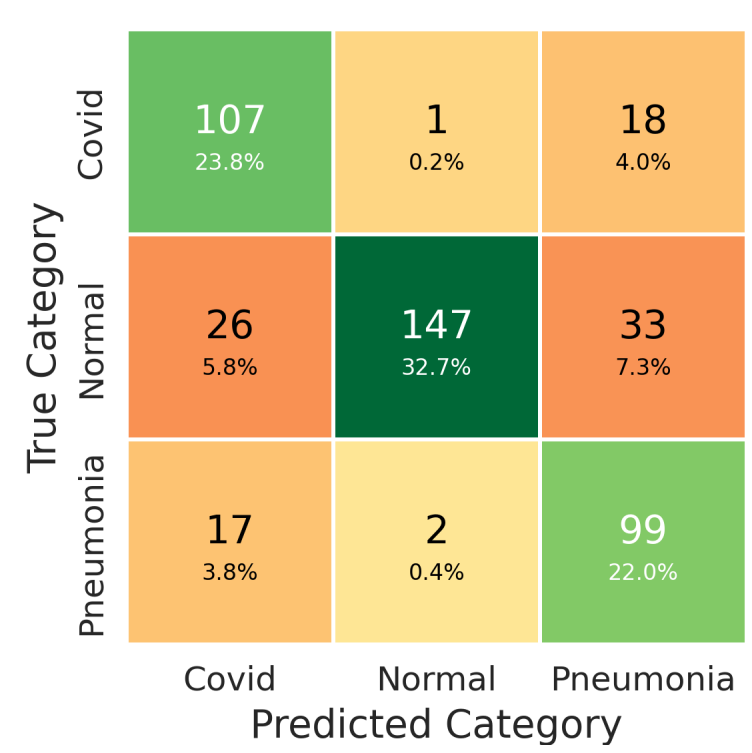

Supplementary Fig. 2. **POLCOVID Confusion Matrix:** we present the confusion matrix of RayDINO on the test set of the POLCOVID dataset.

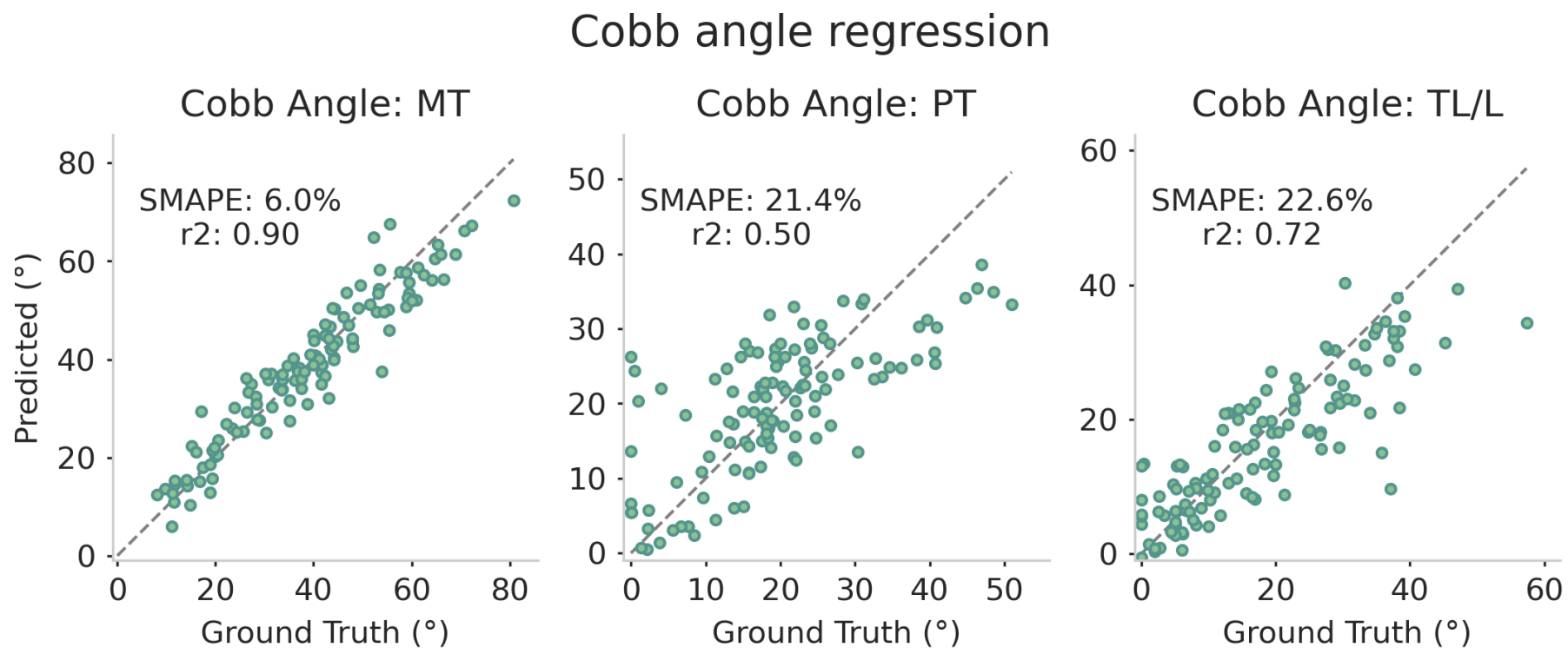

Supplementary Fig. 3. Details of the scoliosis angle regression. We report SMAPE and r^2 on the test set for main thoracic (MT), proximal thoracic (PT) and thoraco-lumbar (TL/L) angles on the test set.

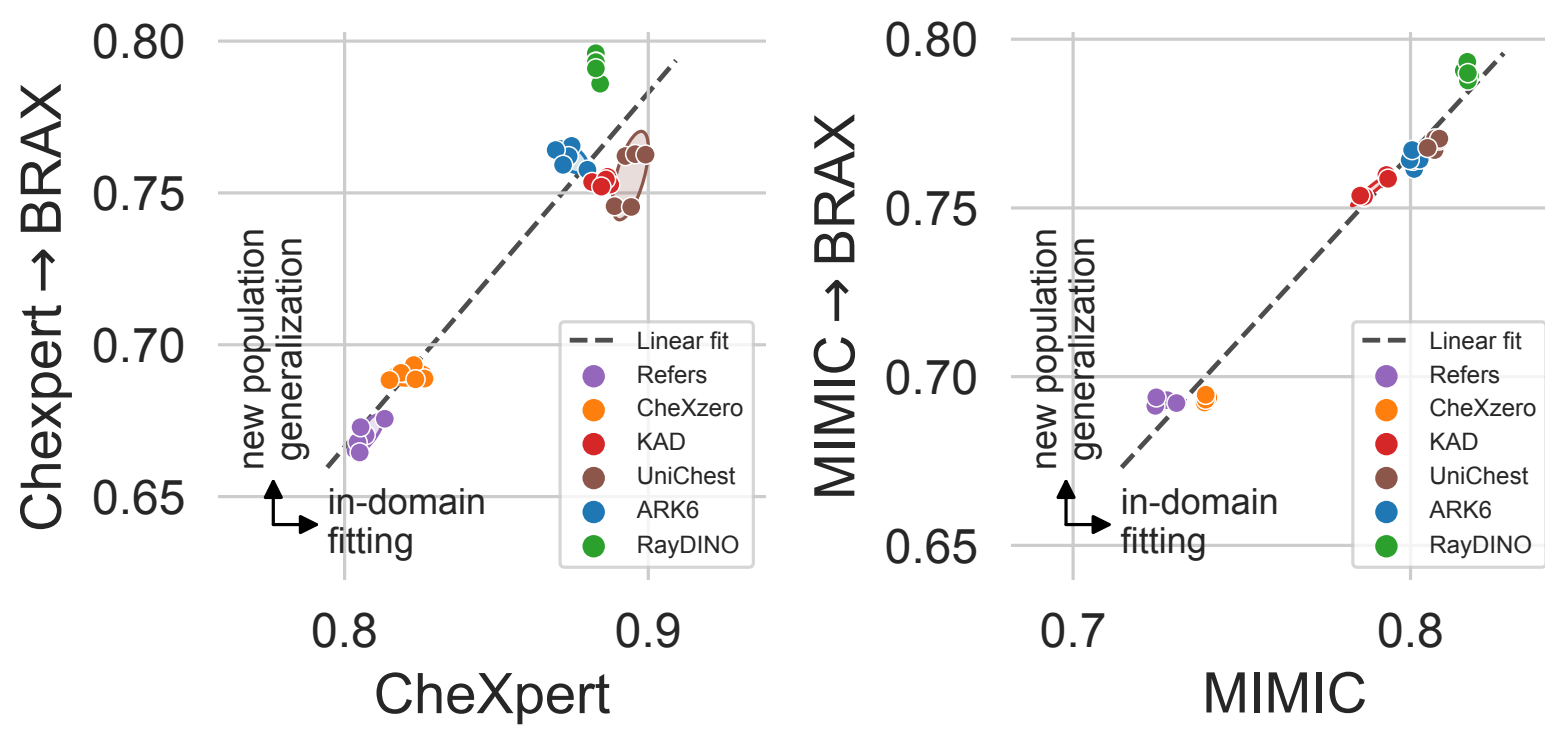

Supplementary Fig. 4. In-Domain versus Out-Of-Domain performances: We compare for each model the correlation between performances on the internal datasets CheXpert and MIMIC and the external dataset BRAX. Colors correspond to the backbone model and each of the 5 dots of the same color correspond to a different seed. We report the 95%CI as ellipses.

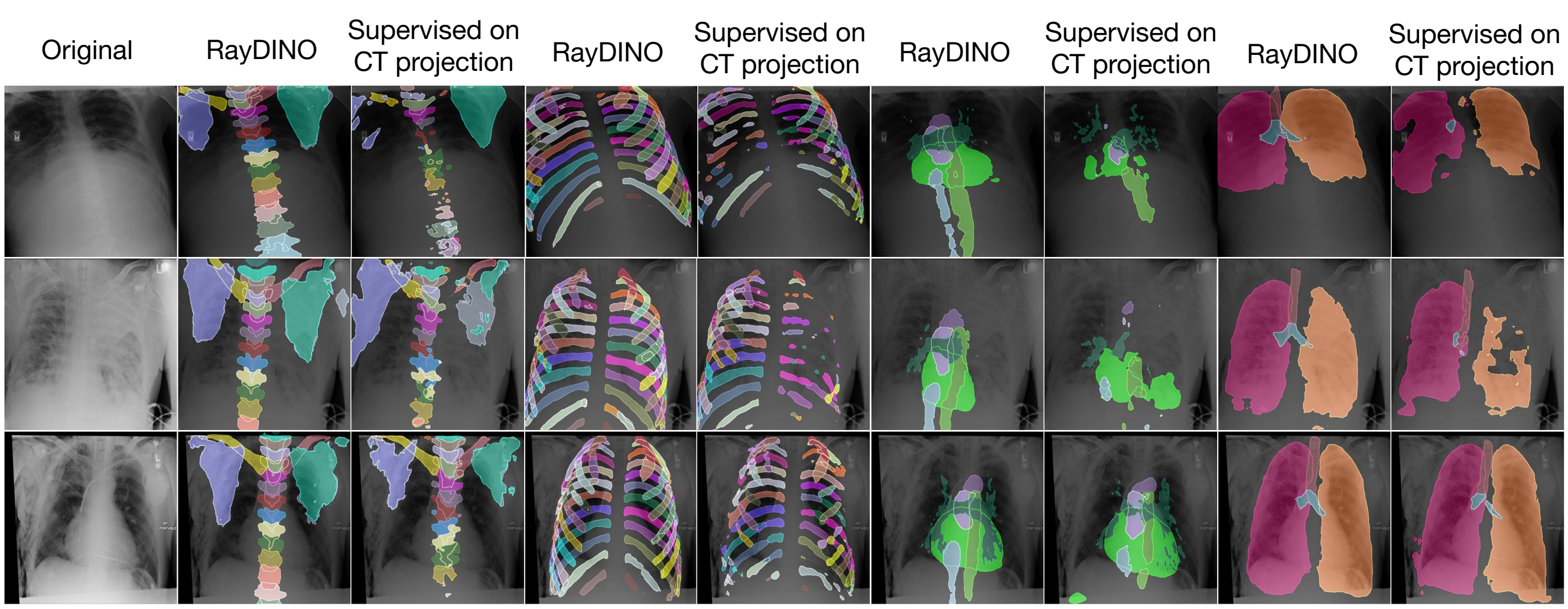

Supplementary Fig. 5. Generalization of segmentation on real X-rays: We present examples of RayDINO's segmentation adapter trained on 2D CT-scan projection and evaluated on real-world X-rays. We compare it with a strong supervised baseline CXAS[34].

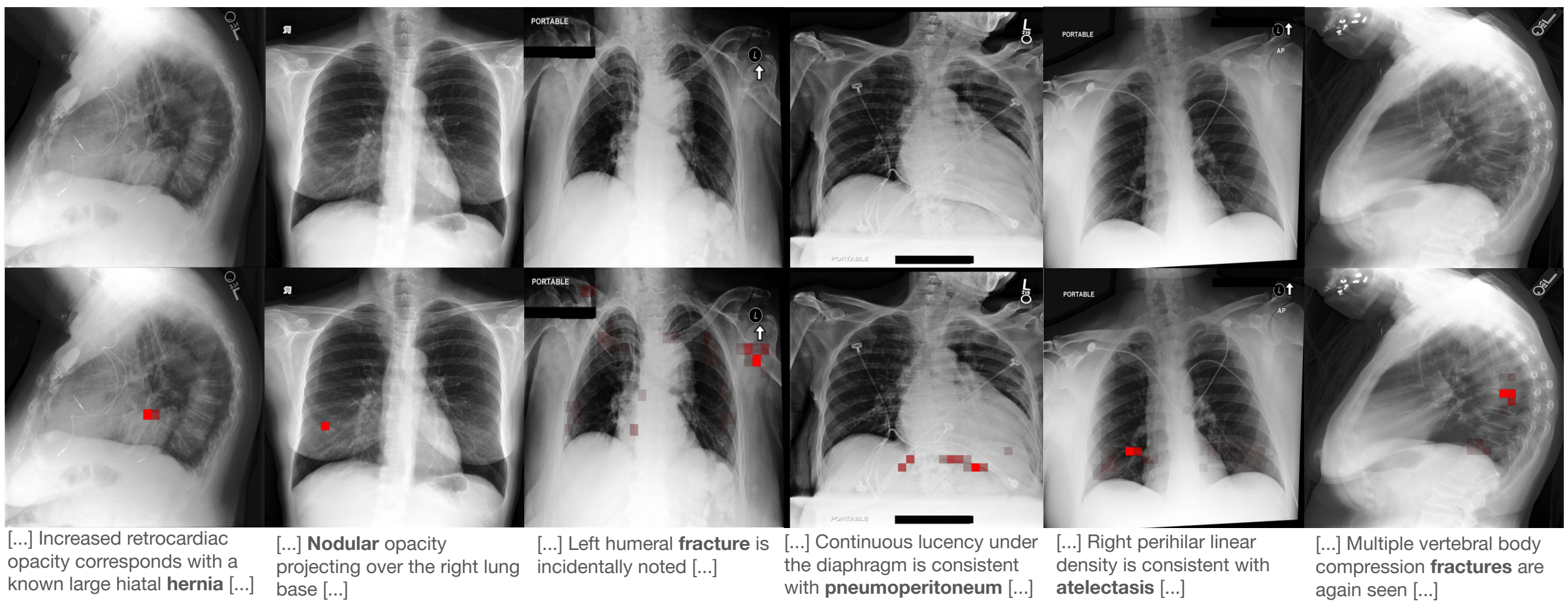

Supplementary Fig. 6. Correlating attention maps with radiologist reports: We present additional examples of RayDINO's attention maps alongside radiologist reports (specific finding of the classifier highlighted in bold) to demonstrate the accurate localization of the interpretability of RayDINO.

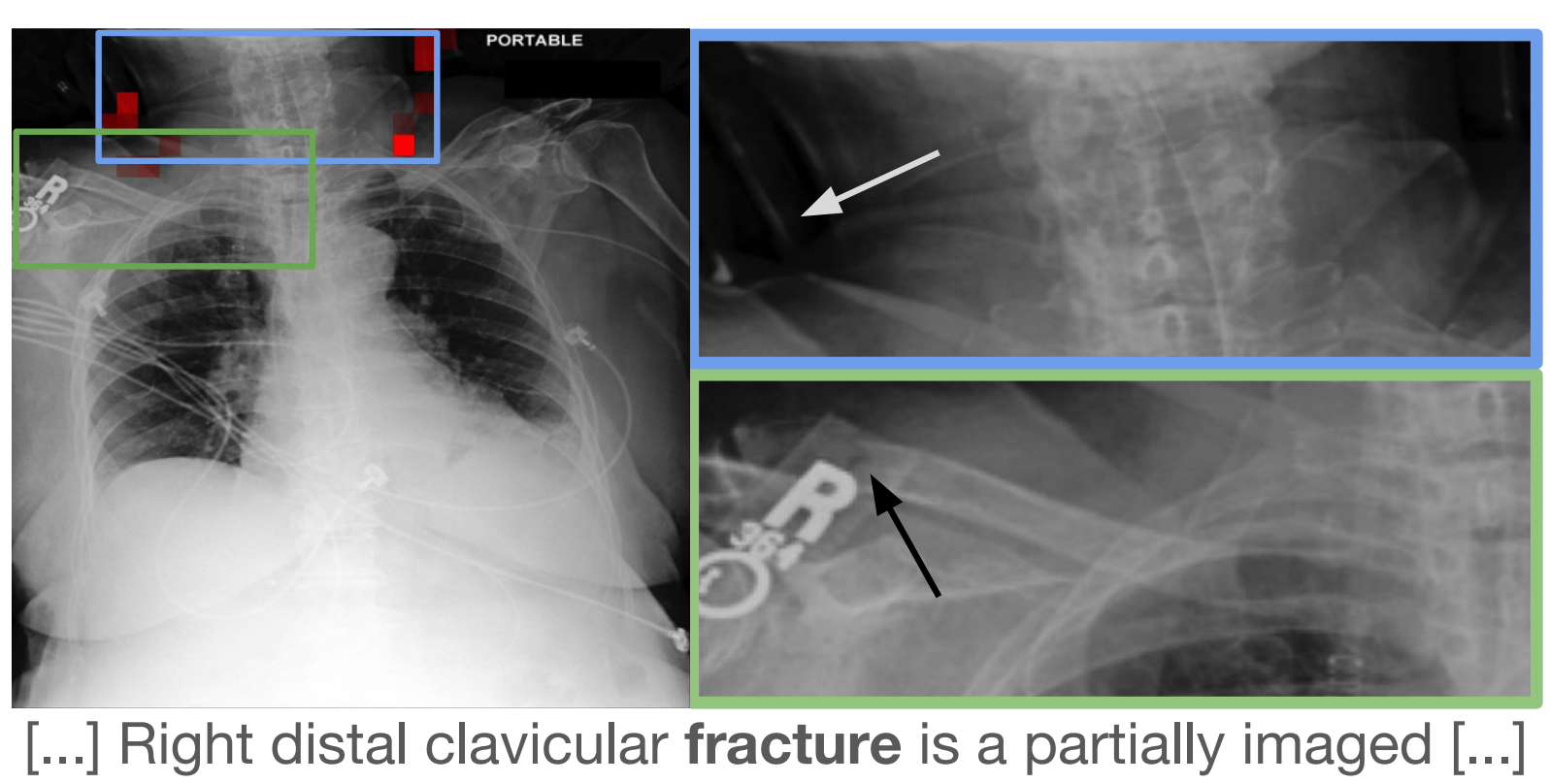

Supplementary Fig. 7. Identifying failure case through RayDINO's interpretability: We highlight a failure case where RayDINO incorrectly predicts a fracture by focusing on the patient's rigid neck brace (top rectangle in blue, white arrow). It fails to detect the actual clavicle fracture (bottom rectangle in green, black arrow) due to its partial imaging.

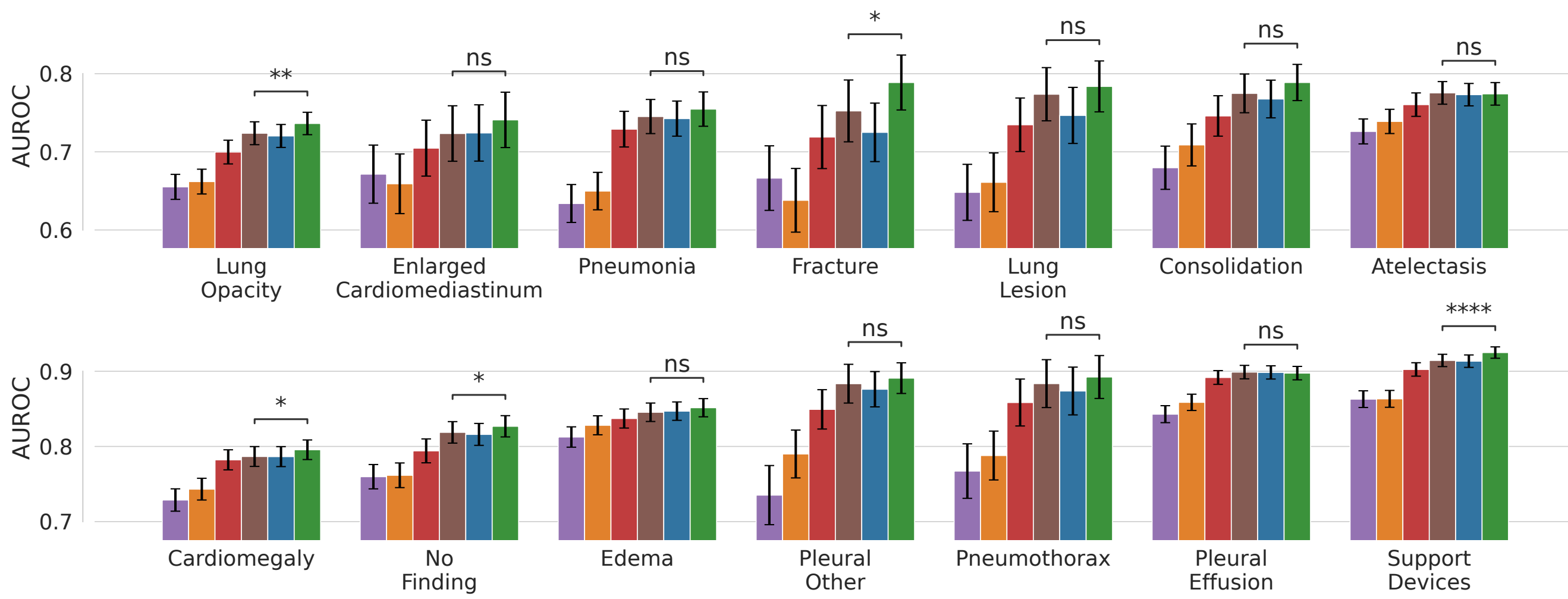

Supplementary Fig. 8. Individual class comparison for MIMIC. Statistical comparison between RayDINO and the best competing model in average using DeLong[25] test.

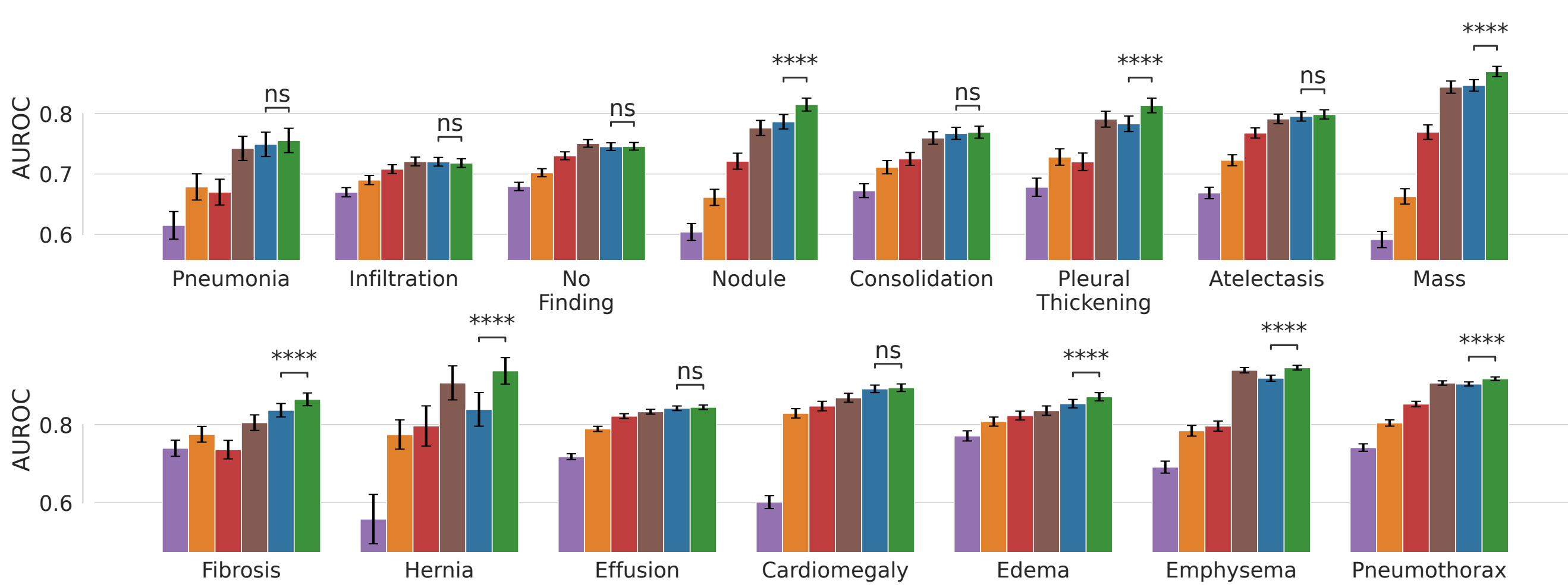

Supplementary Fig. 9. Individual class comparison for NIH. Statistical comparison between RayDINO and the best competing model in average using DeLong[25] test.

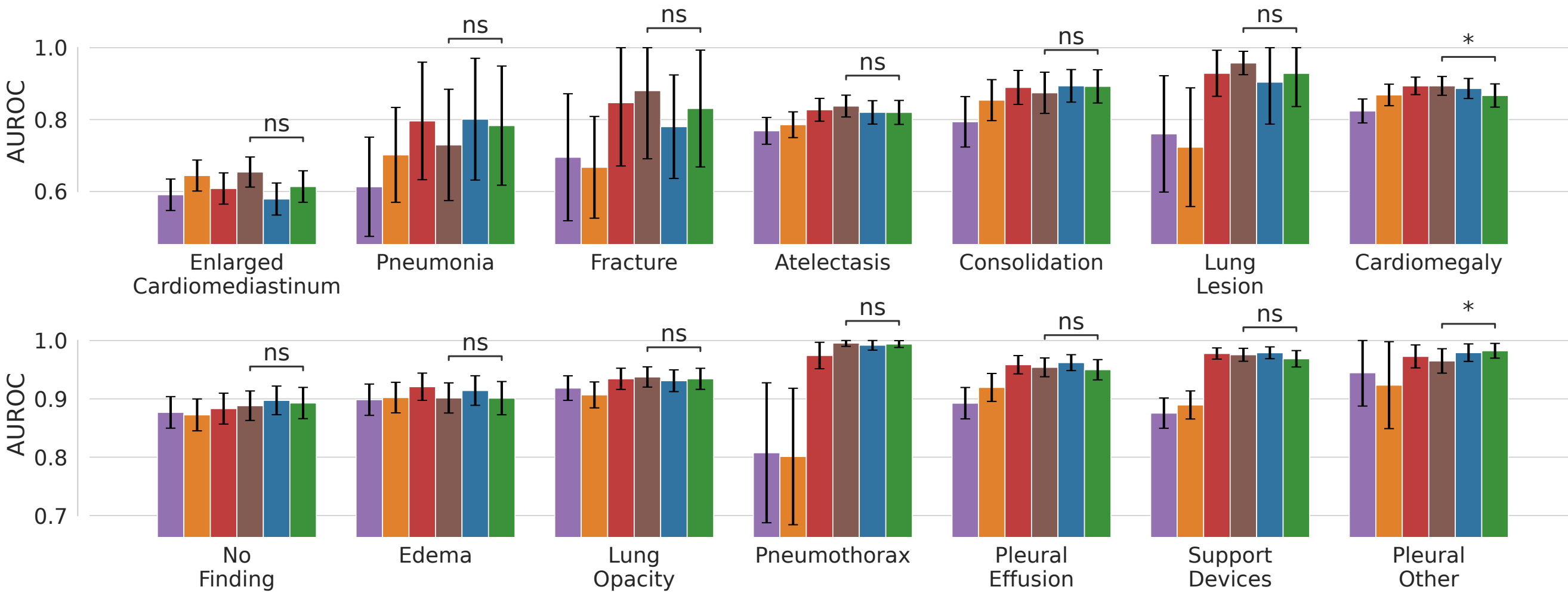

Supplementary Fig. 10. Individual class comparison for CheXpert. Statistical comparison between RayDINO and the best competing model in average using DeLong[25] test.

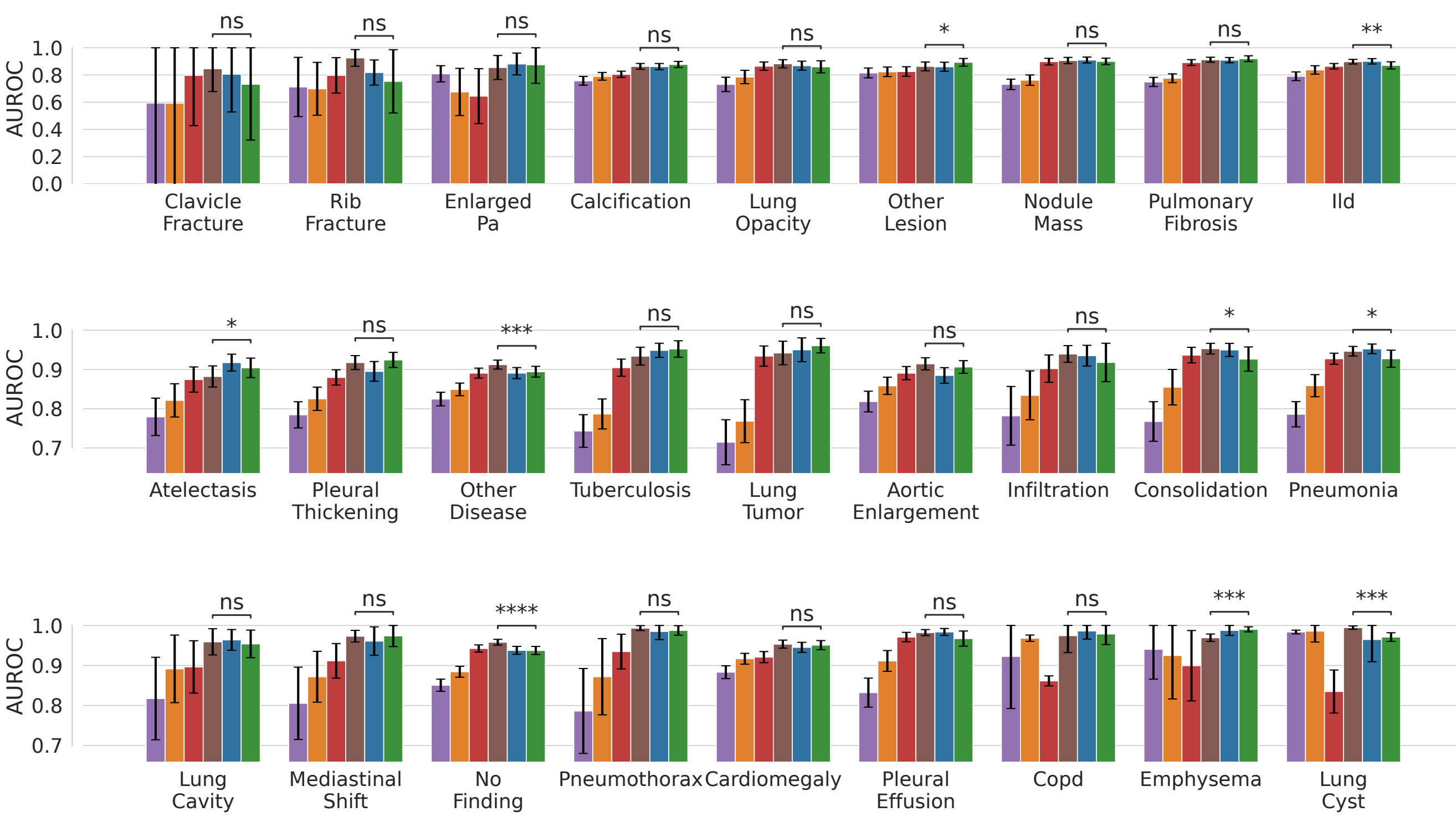

Supplementary Fig. 11. Individual class comparison for Vindr. Statistical comparison between RayDINO and the best competing model in average using DeLong[25] test.

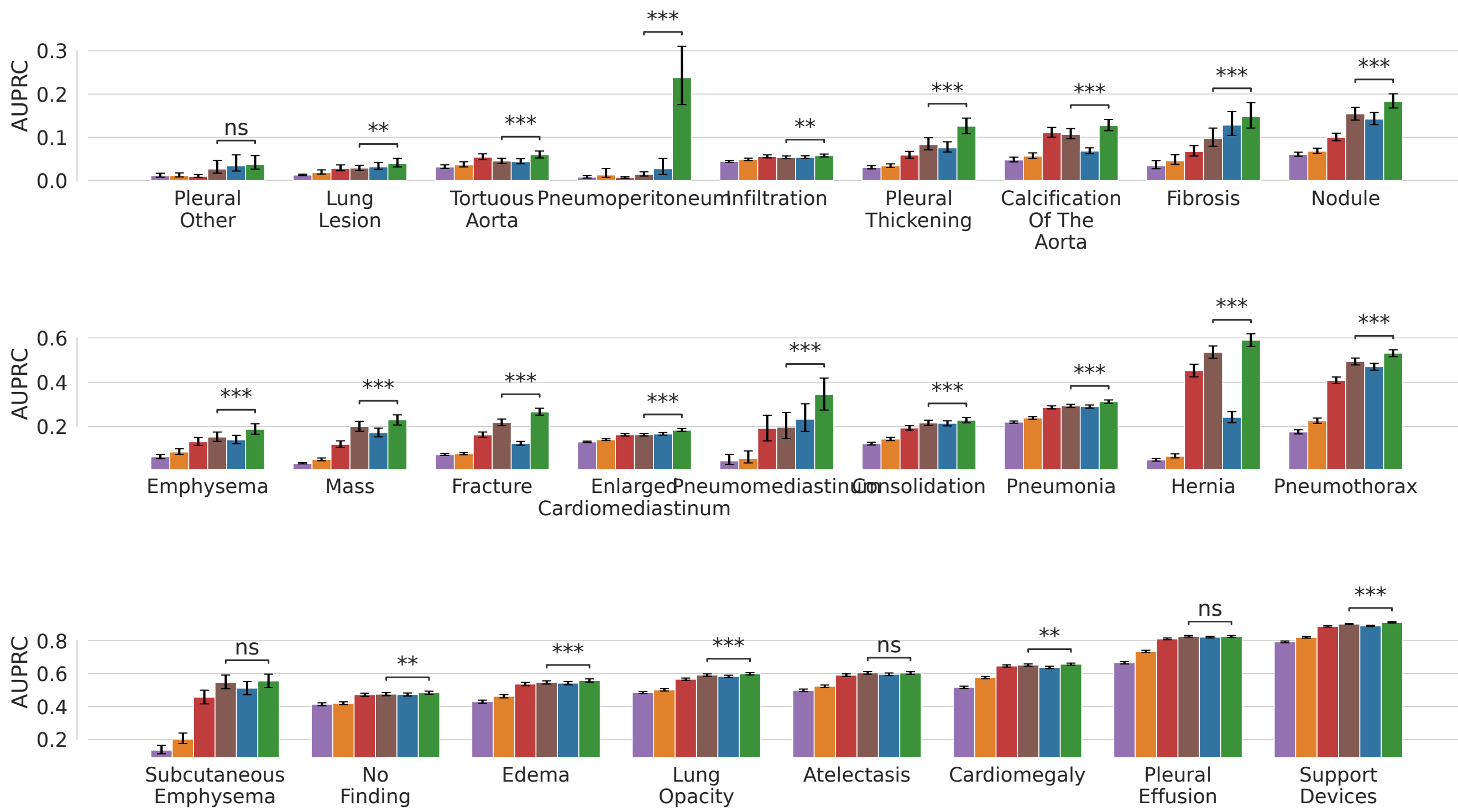

Supplementary Fig. 12. Individual class comparison for MIMIC Long-Tail. Statistical comparison between RayDINO and the best competing model in average using bootstrapped paired permutation test.

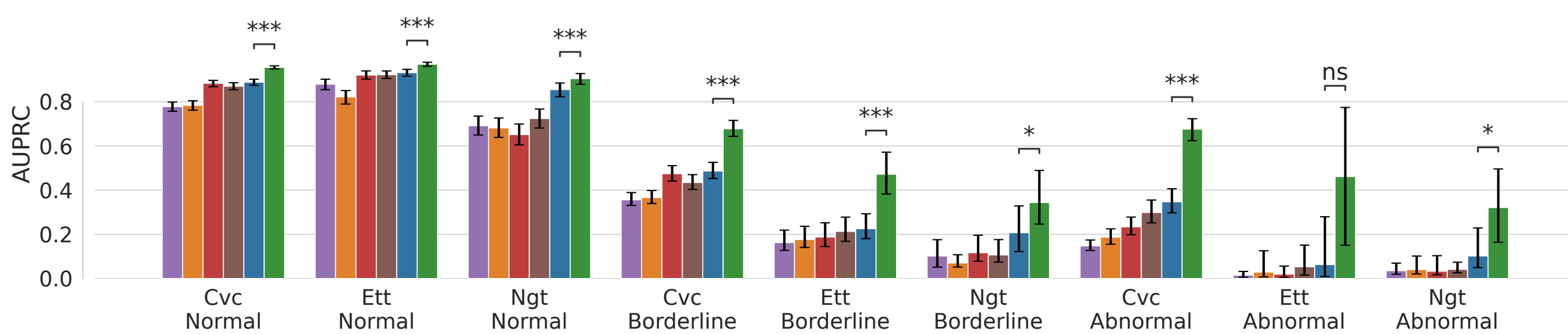

Supplementary Fig. 13. Individual class comparison for Catheter prediction. Statistical comparison between RayDINO and the best competing model in average using bootstrapped paired permutation test.

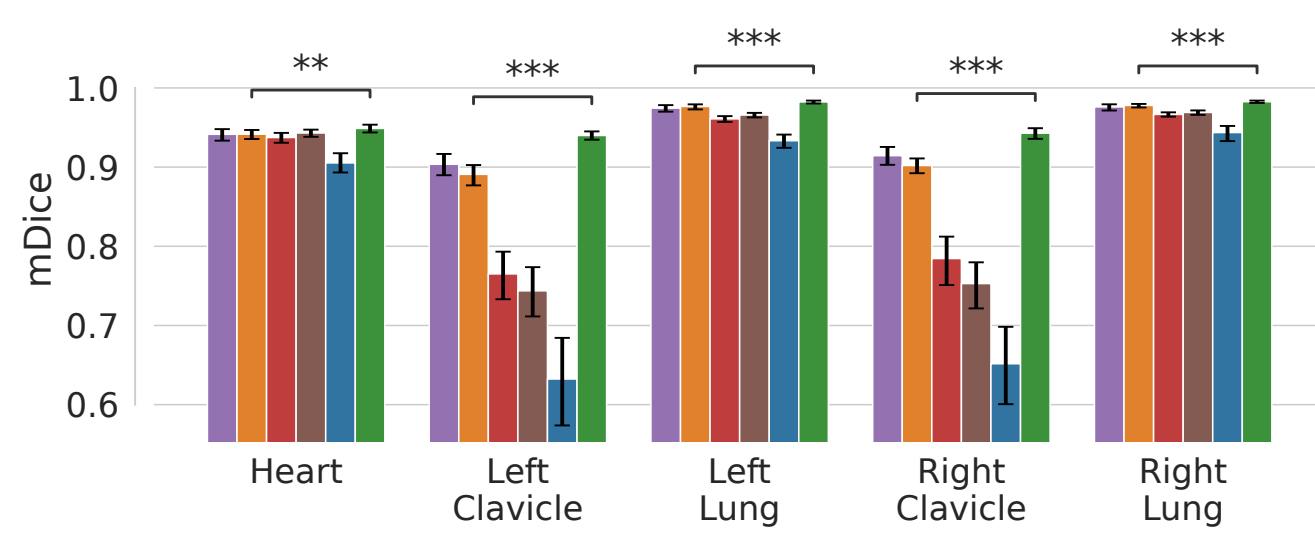

Supplementary Fig. 14. Individual class comparison for JSRT segmentation. Statistical comparison between RayDINO and the best competing model in average using bootstrapped paired permutation test.

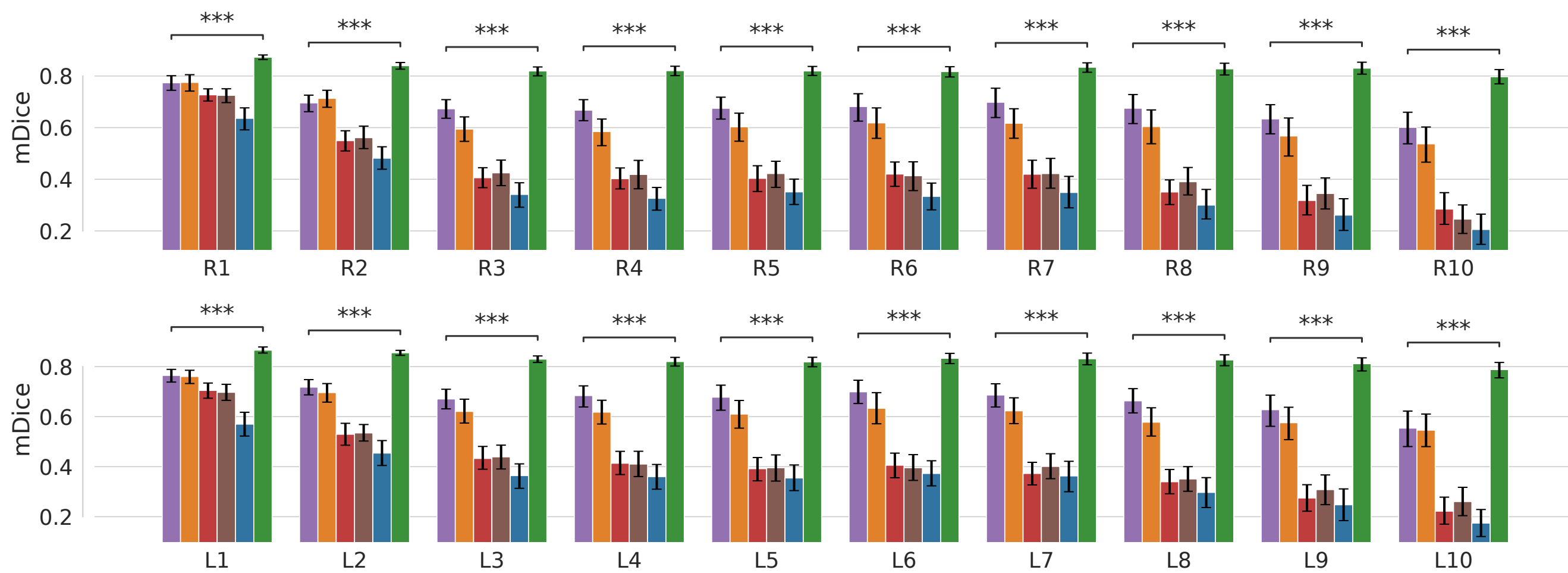

Supplementary Fig. 15. Individual class comparison for Vindr Rib segmentation. Statistical comparison between RayDINO and the best competing model in average using bootstrapped paired permutation test.

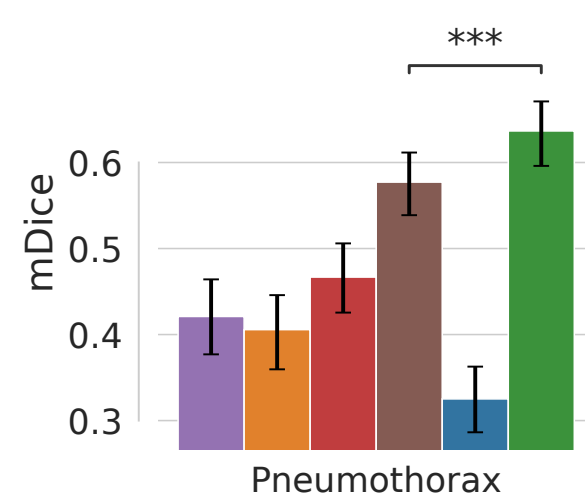

Supplementary Fig. 16. Individual class comparison for Pneumothorax segmentation. Statistical comparison between RayDINO and the best competing model in average using bootstrapped paired permutation test.

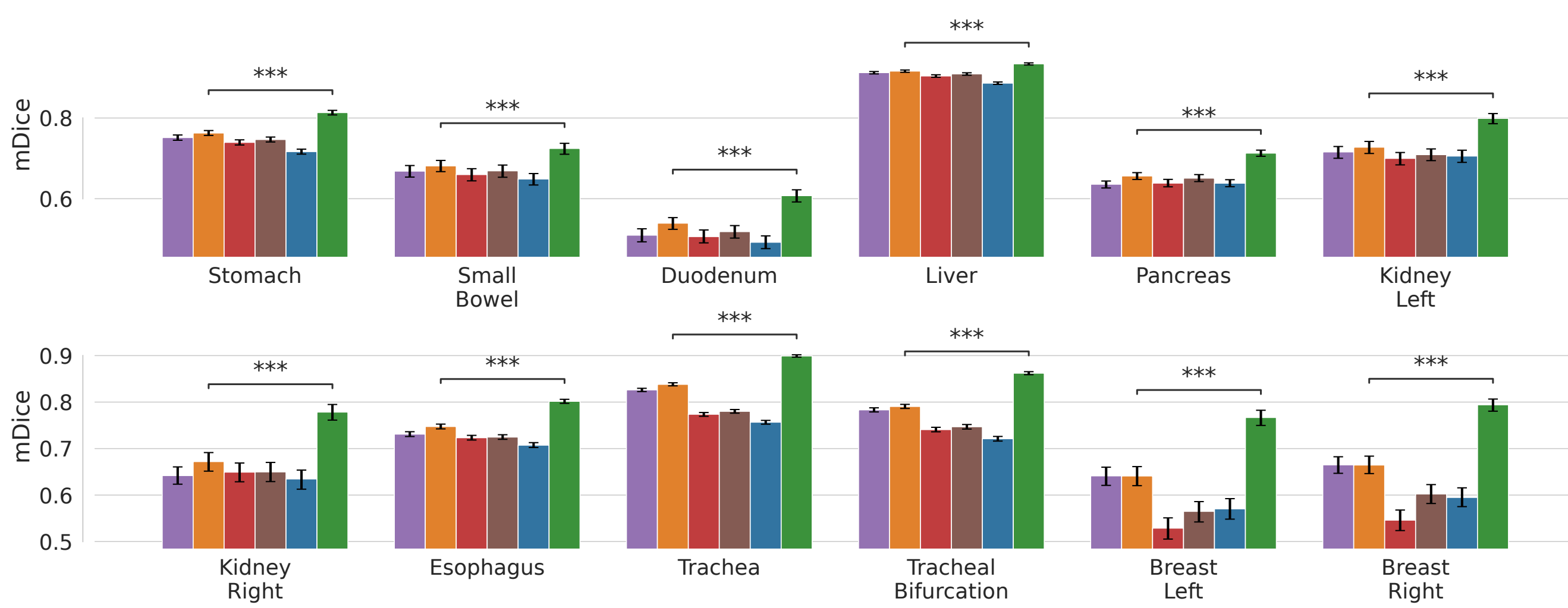

Supplementary Fig. 17. Individual class comparison for **abdomen organs** segmentation from the PAXRay dataset. Statistical comparison between RayDINO and the best competing model in average using bootstrapped paired permutation test.

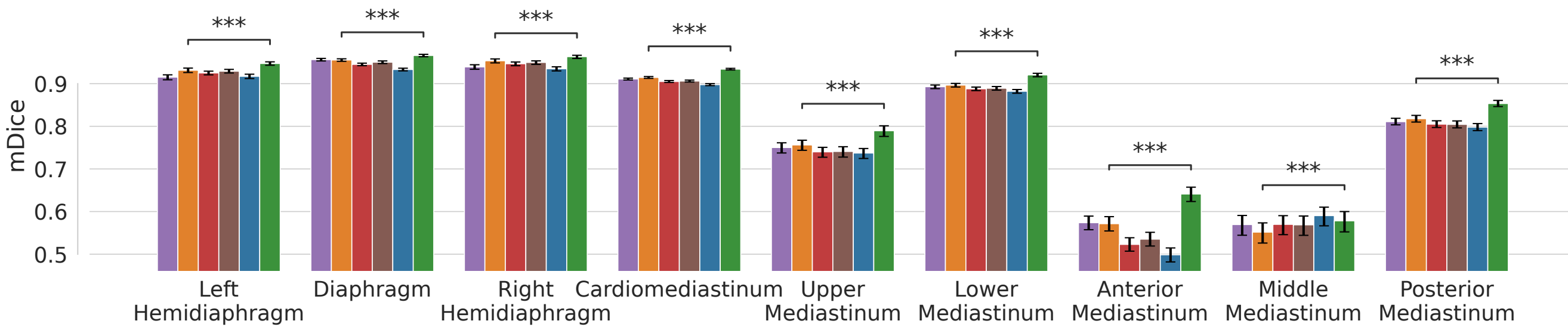

Supplementary Fig. 18. Individual class comparison for **diaphragm** and **mediastinum** segmentation from the PAXRay dataset. Statistical comparison between RayDINO and the best competing model in average using bootstrapped paired permutation test.

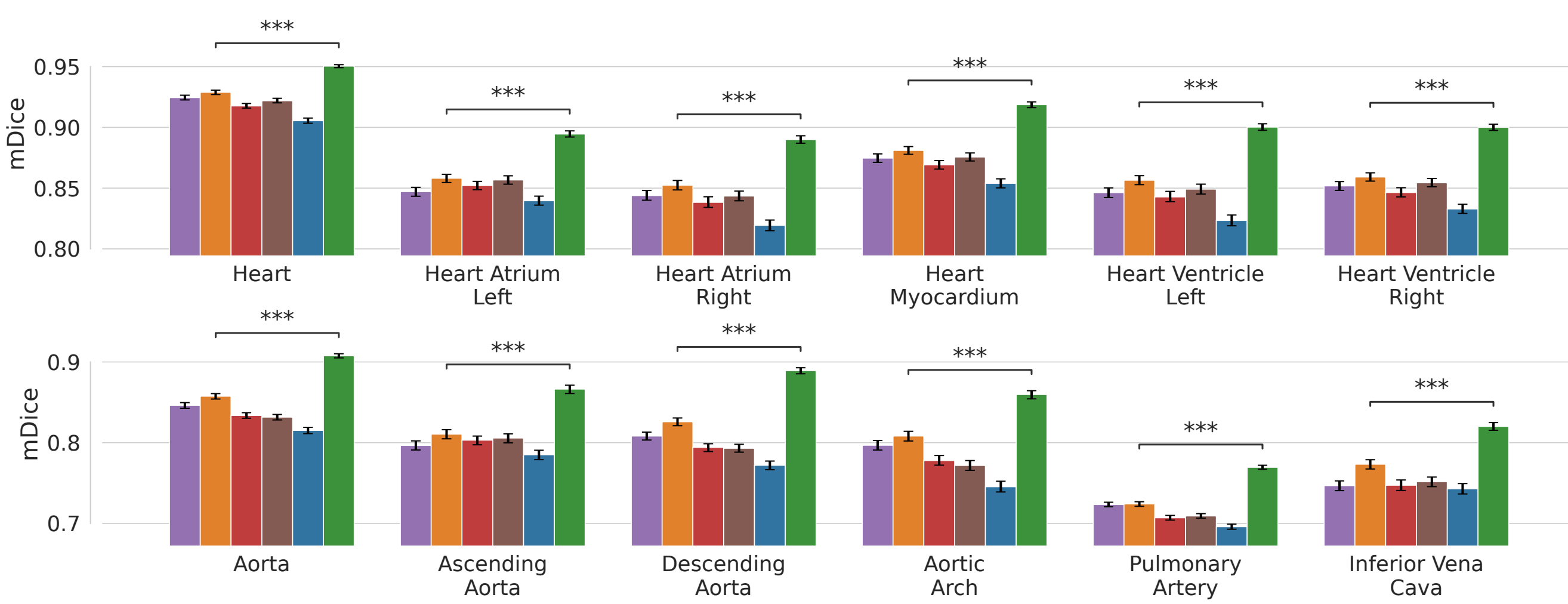

Supplementary Fig. 19. Individual class comparison for **heart related** organs segmentation from the PAXRay dataset. Statistical comparison between RayDINO and the best competing model in average using bootstrapped paired permutation test.

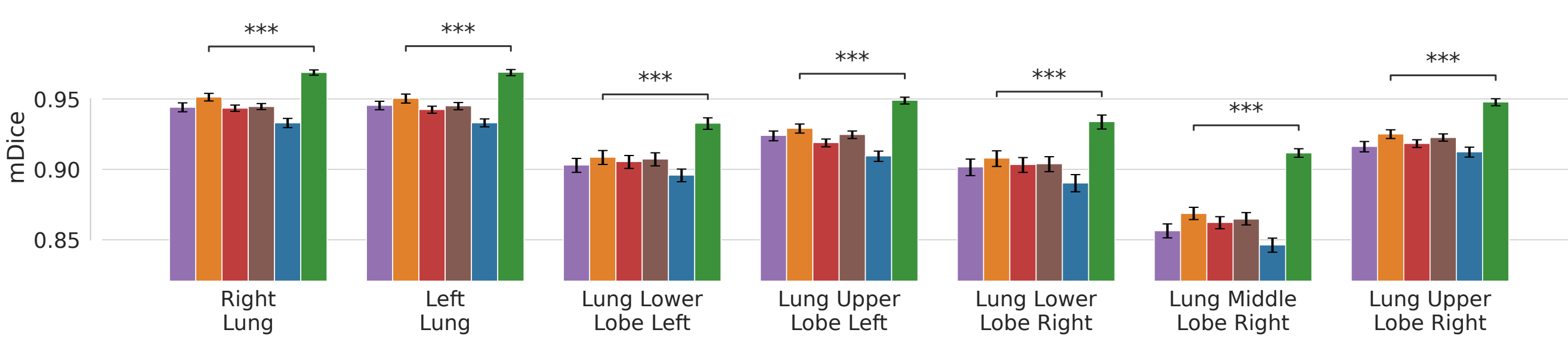

Supplementary Fig. 20. Individual class comparison for **lungs** segmentation from the PAXRay dataset. Statistical comparison between RayDINO and the best competing model in average using bootstrapped paired permutation test.

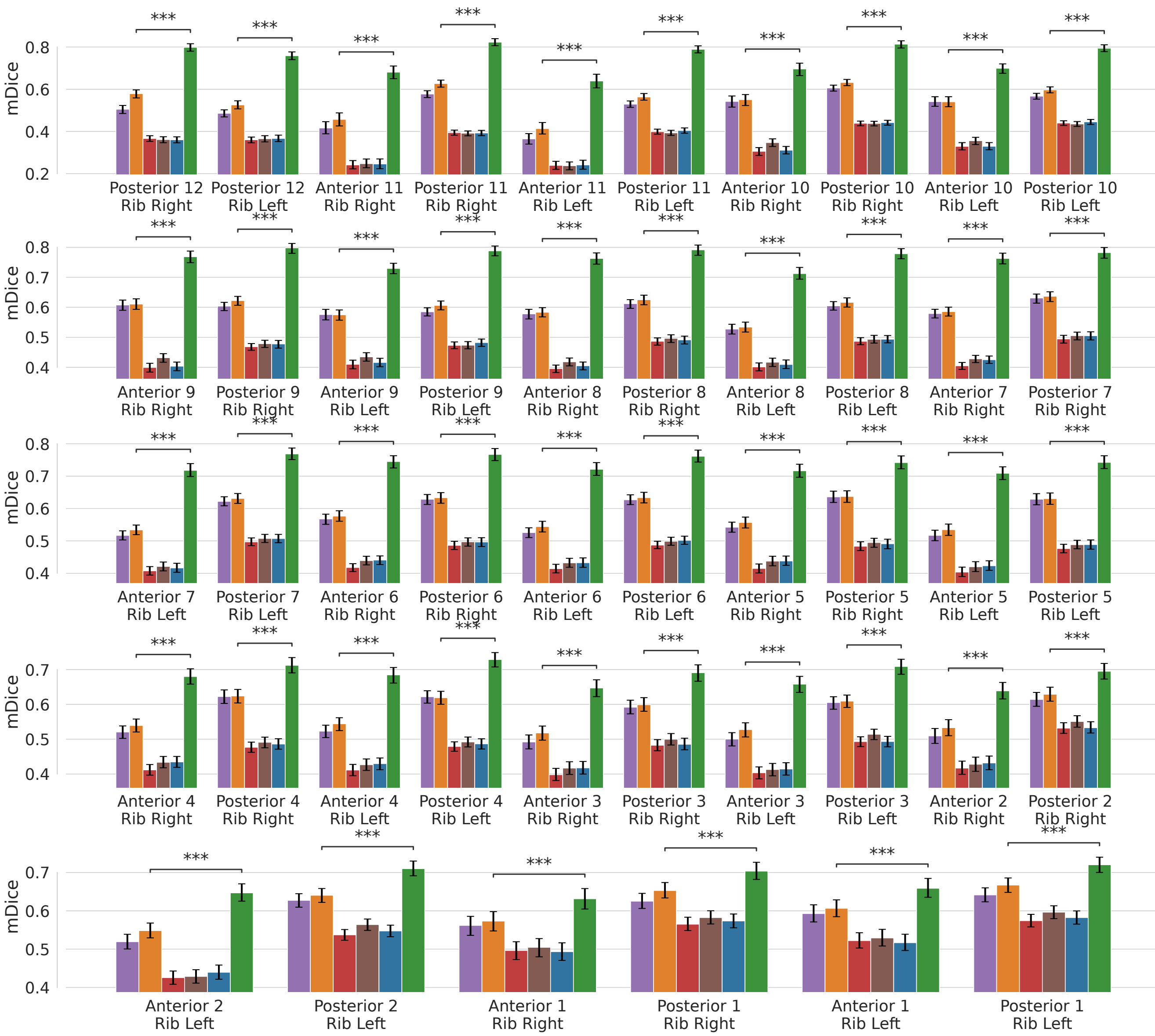

Supplementary Fig. 21. Individual class comparison for **rib** segmentation from the PAXRay dataset. Statistical comparison between RayDINO and the best competing model in average using bootstrapped paired permutation test.

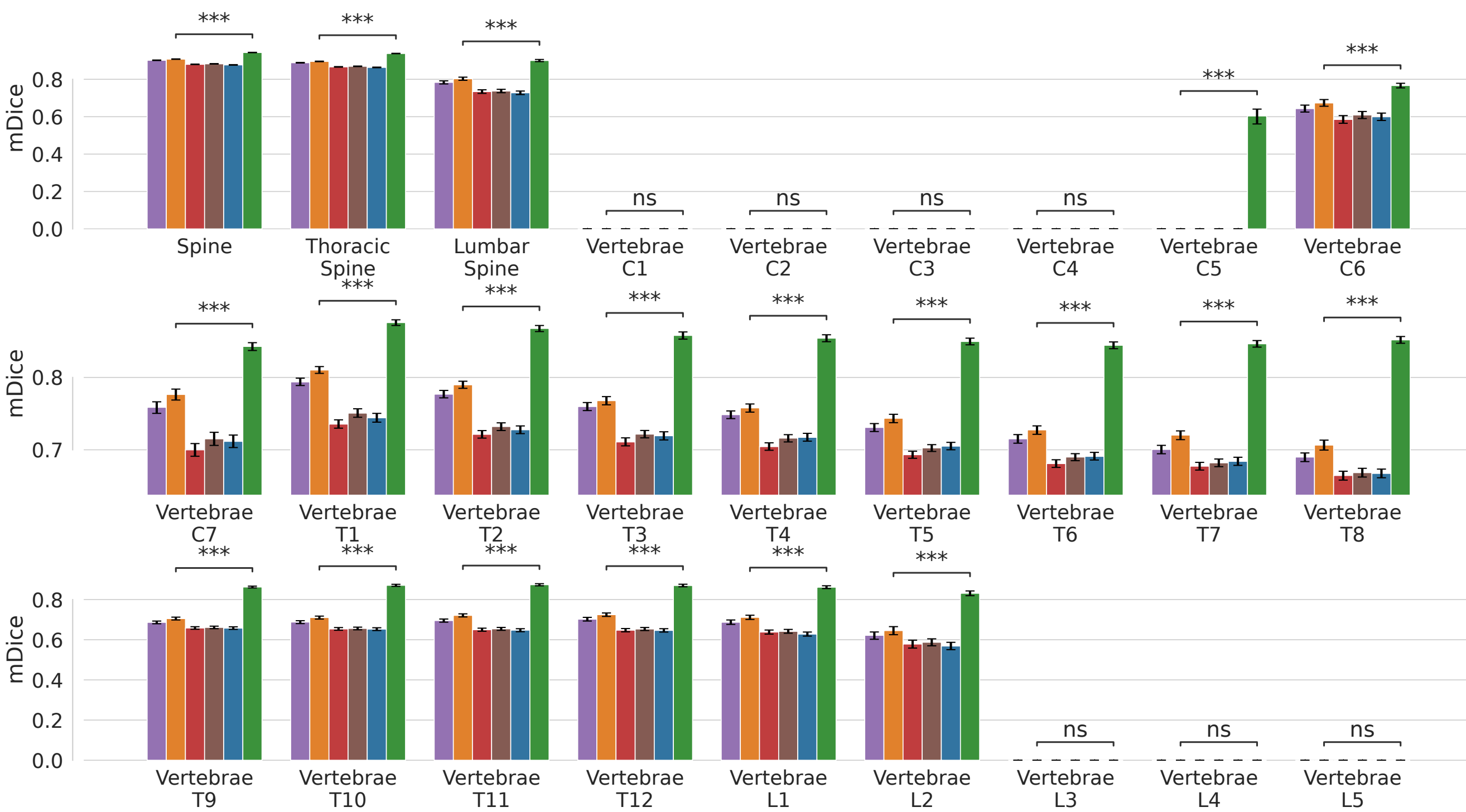

Supplementary Fig. 22. Individual class comparison for **spine** segmentation from the PAXRay dataset. Statistical comparison between RayDINO and the best competing model in average using bootstrapped paired permutation test.

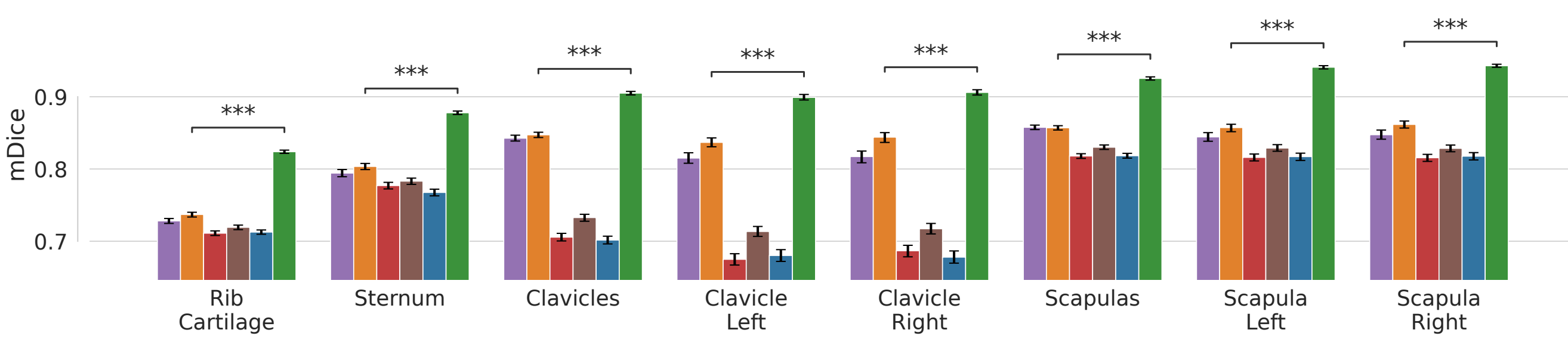

Supplementary Fig. 23. Individual class comparison for **other bones** segmentation from the PAXRay dataset. Statistical comparison between RayDINO and the best competing model in average using bootstrapped paired permutation test.

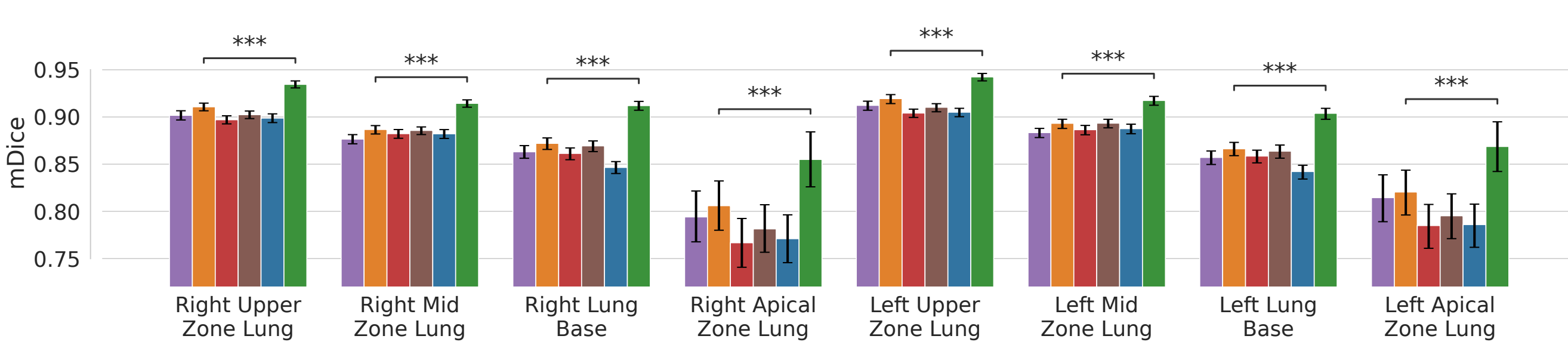

Supplementary Fig. 24. Individual class comparison for different **zones** segmentation from the PAXRay dataset. Statistical comparison between RayDINO and the best competing model in average using bootstrapped paired permutation test.

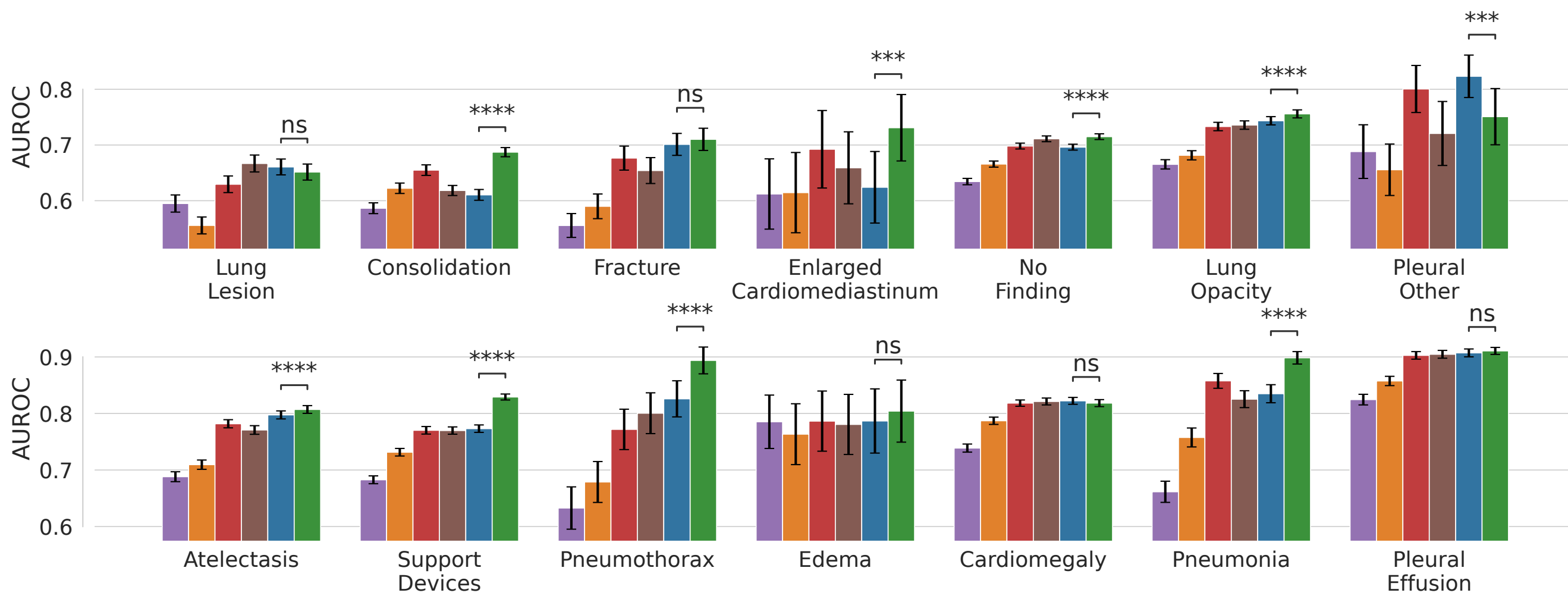

Supplementary Fig. 25. Individual class comparison for classification generalization from CheXpert to BRAX. Statistical comparison between RayDINO and the best competing model in average using DeLong[25] test.

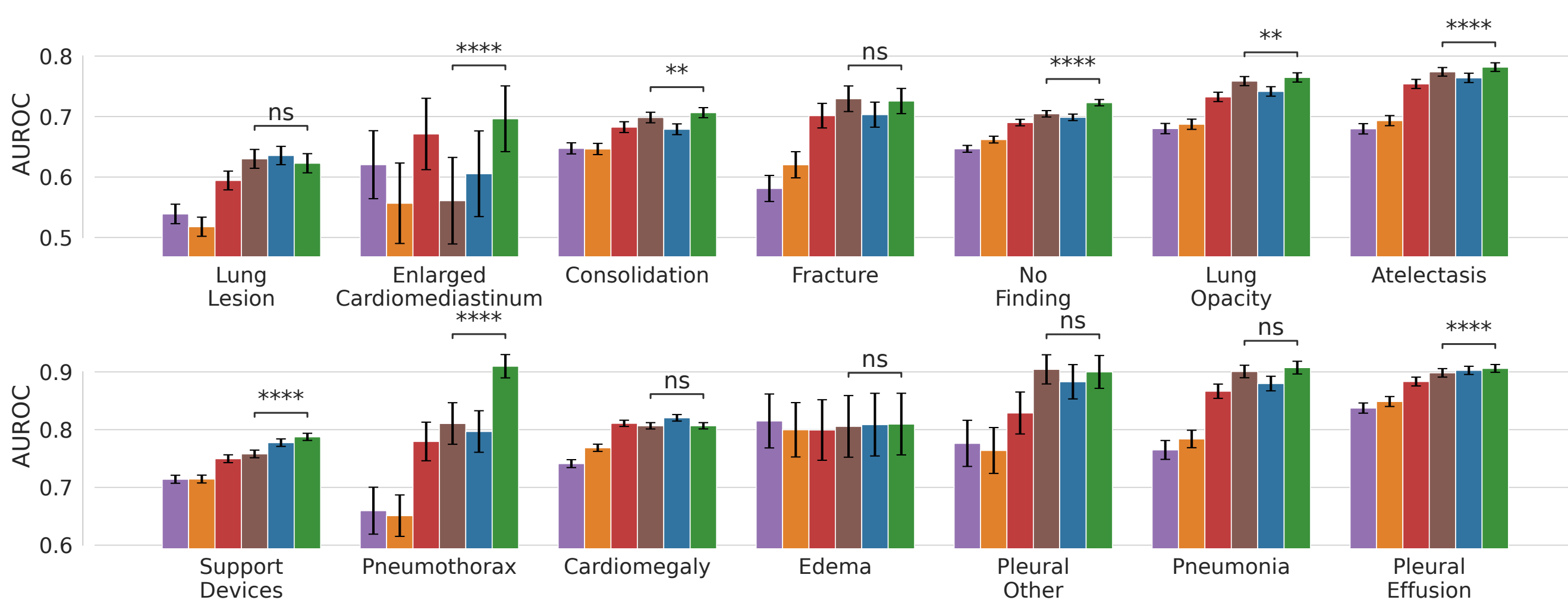

Supplementary Fig. 26. Individual class comparison for classification generalization from MIMIC to BRAX. Statistical comparison between RayDINO and the best competing model in average using DeLong[25] test.

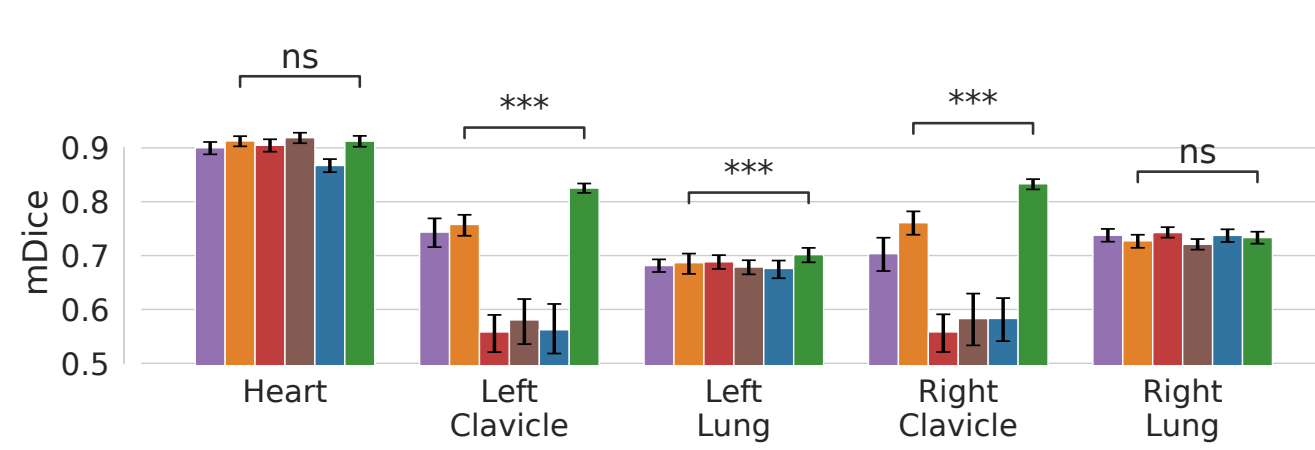

Supplementary Fig. 27. Individual class comparison for segmentation generalization from PAXRay to JSRT. Statistical comparison between RayDINO and the best competing model in average using bootstrapped paired permutation test.

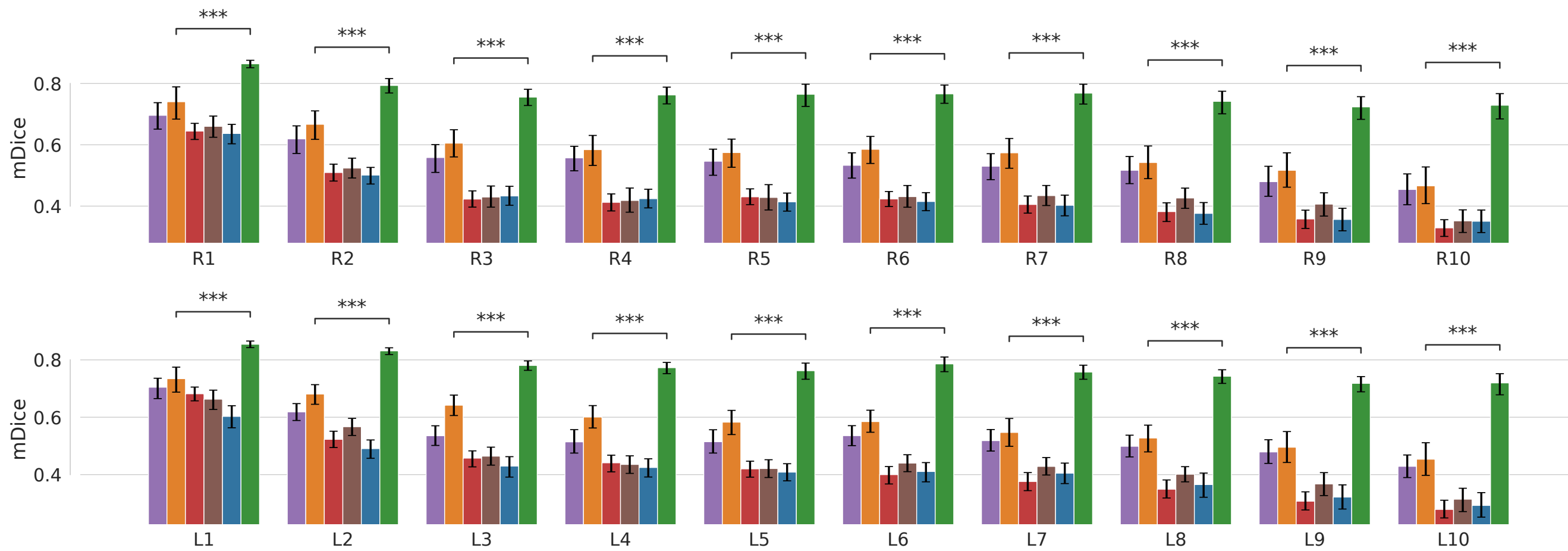

Supplementary Fig. 28. Individual class comparison for rib segmentation on Vindr Rib. Statistical comparison between RayDINO and the best competing model in average using bootstrapped paired permutation test.

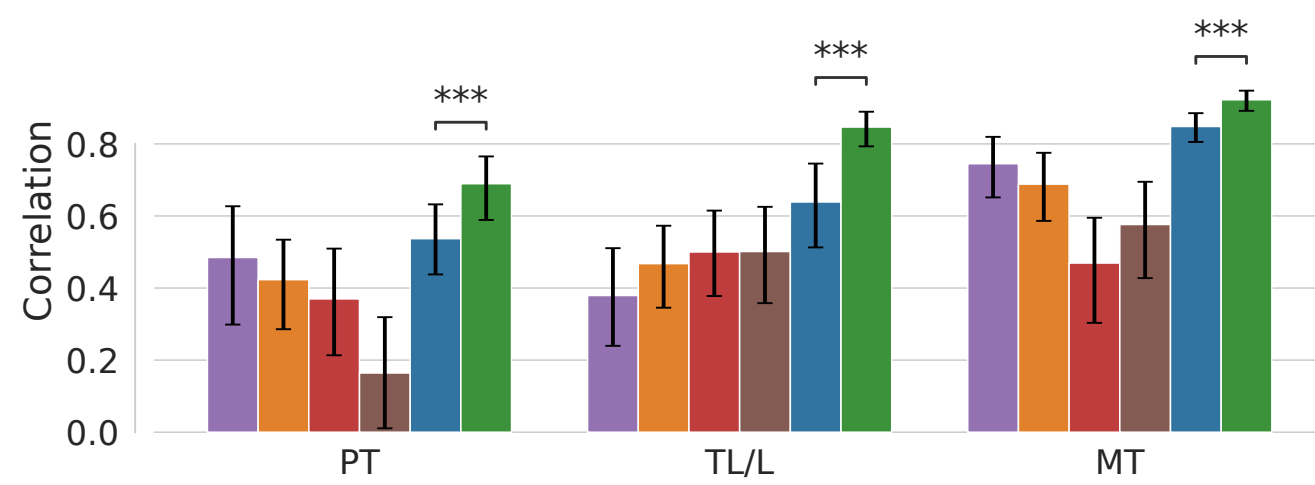

Supplementary Fig. 29. Individual class comparison for scoliosis angle regression. Statistical comparison between RayDINO and the best competing model in average using bootstrapped paired permutation test.

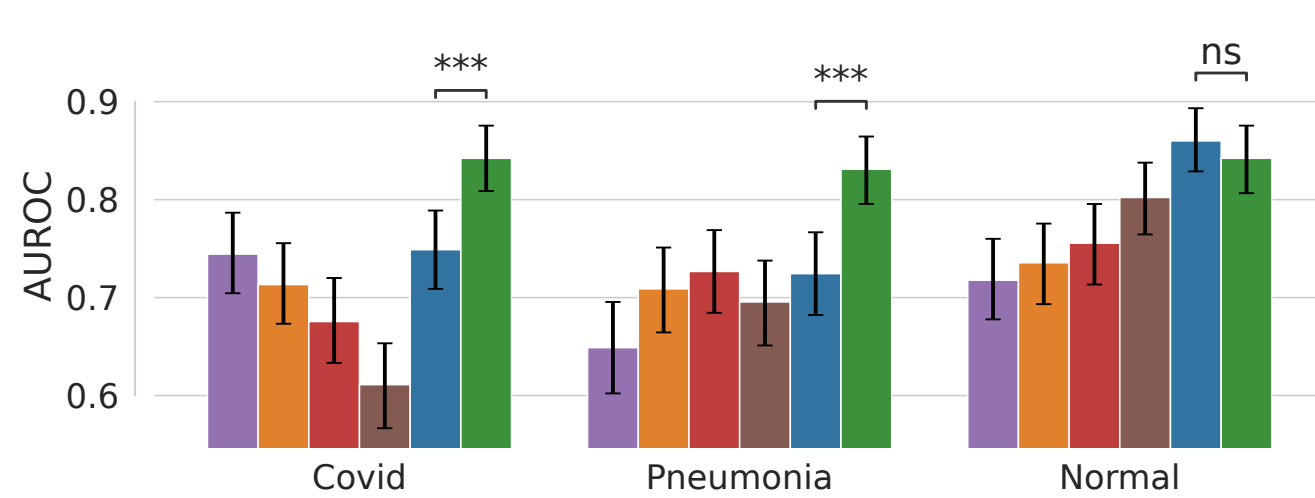

Supplementary Fig. 30. Individual class comparison for unseen disease classification (COVID-19). Statistical comparison between RayDINO and the best competing model in average using bootstrapped paired permutation test.

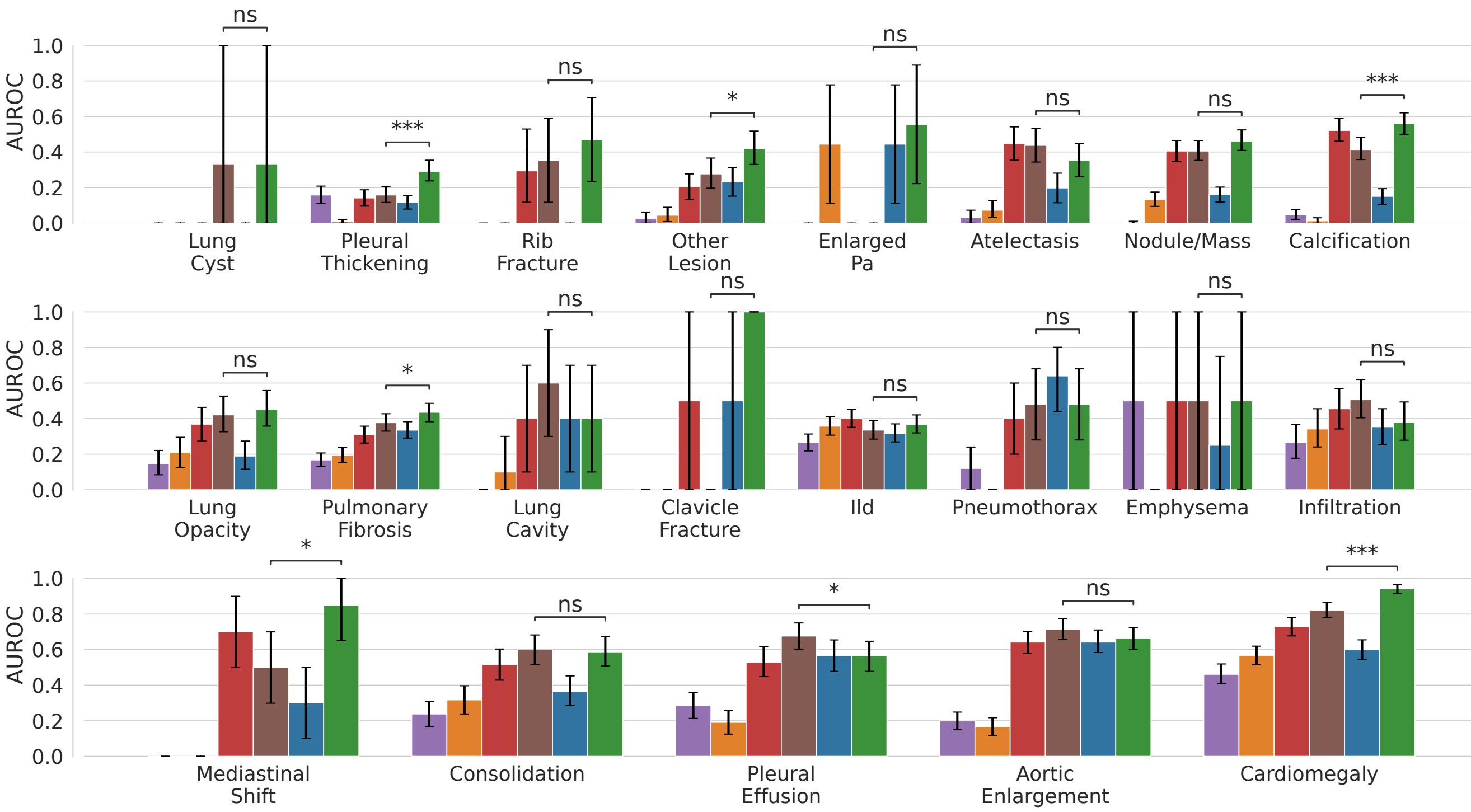

Supplementary Fig. 31. Individual class comparison for attention pinpointing (interpretability) on Vindr. Statistical comparison between RayDINO and the best competing model in average using bootstrapped paired permutation test.

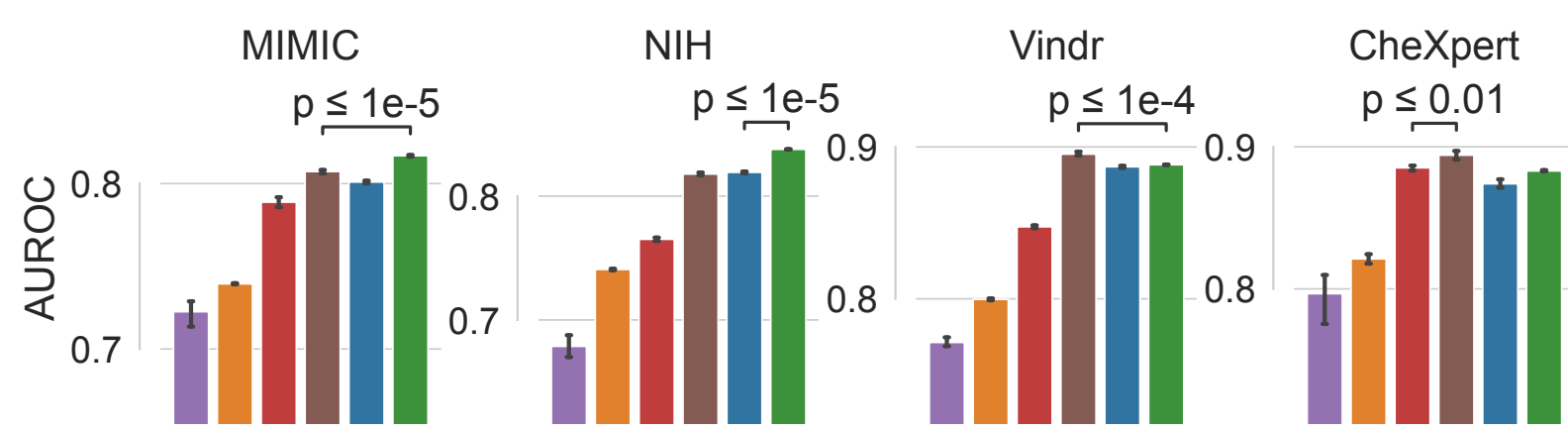

Supplementary Fig. 32. Comparison for classification performances across 5 seeds. Statistical comparison between RayDINO and the best competing model using t-test.

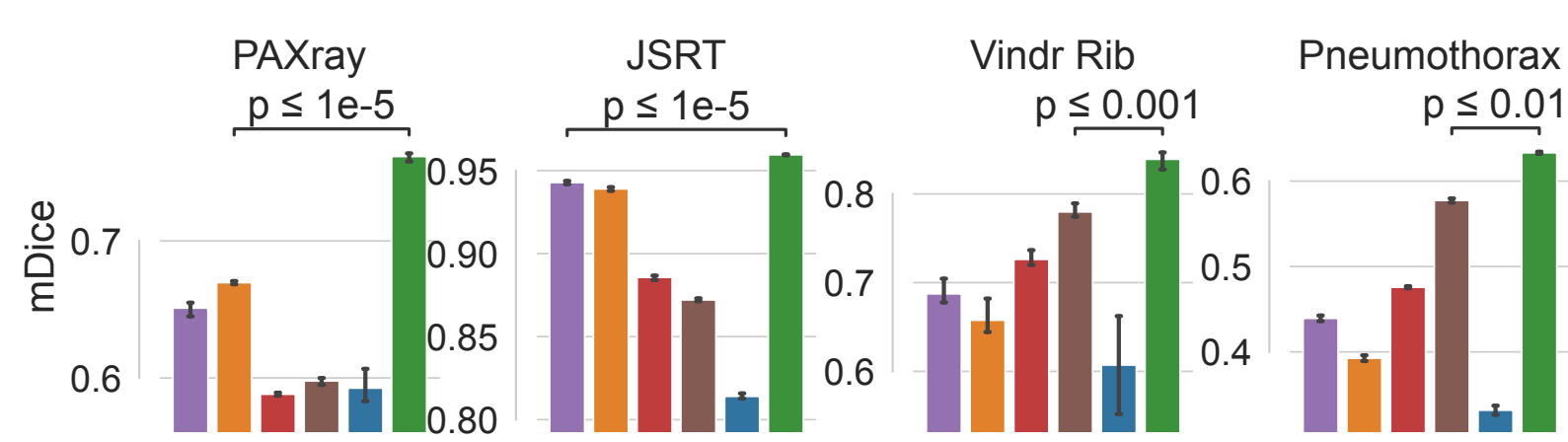

Supplementary Fig. 33. Comparison for segmentation performances across 5 seeds. Statistical comparison between RayDINO and the best competing model using t-test.

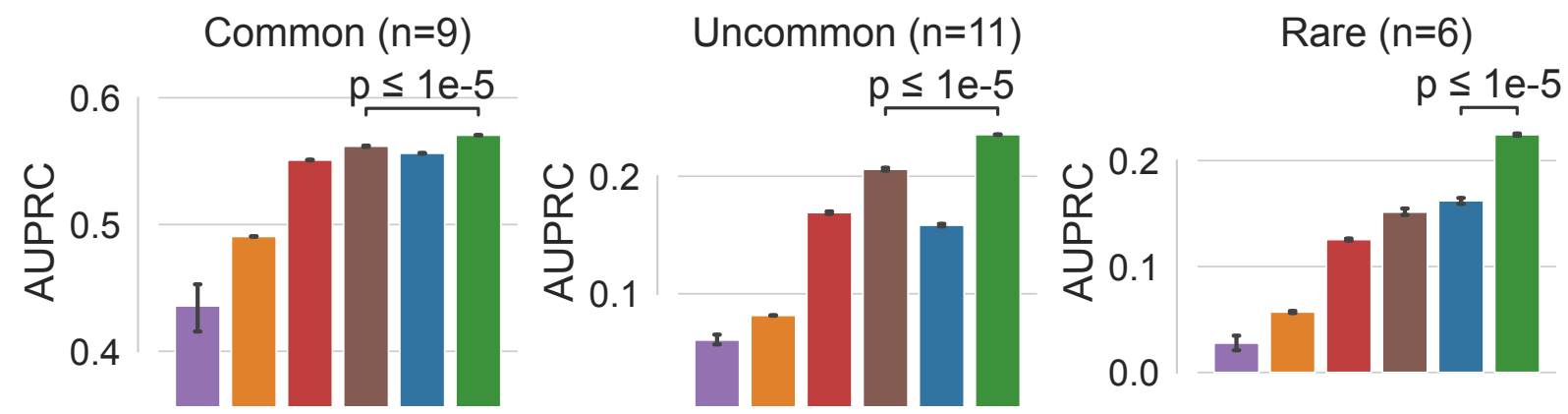

Supplementary Fig. 34. Comparison for MIMIC long-tail classification performances across 5 seeds. Statistical comparison between RayDINO and the best competing model using t-test.

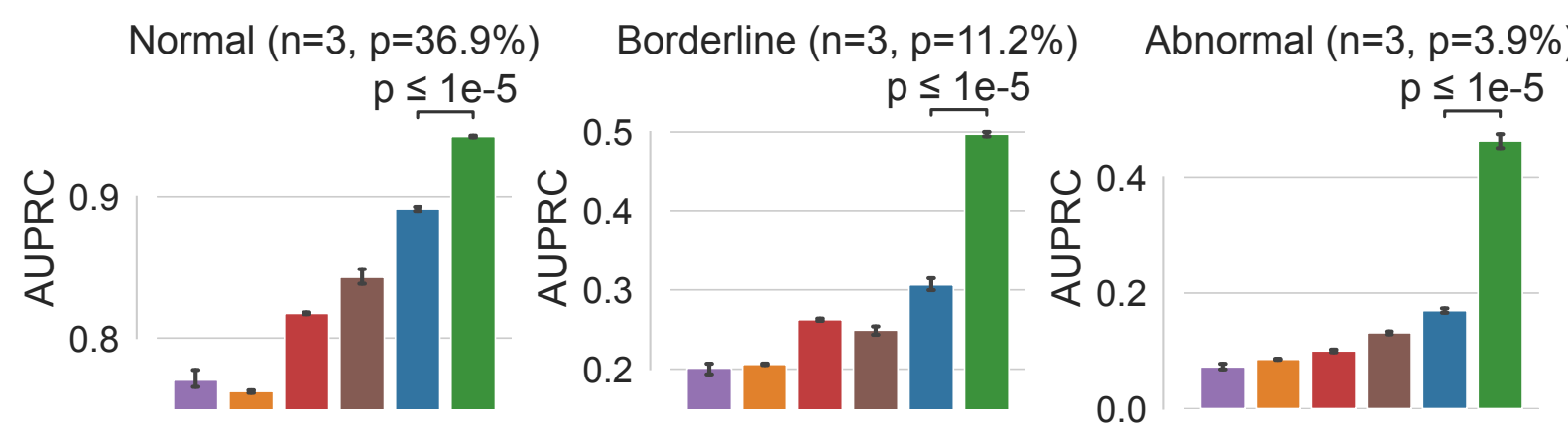

Supplementary Fig. 35. Comparison for Catheter long-tail classification performances across 5 seeds. Statistical comparison between RayDINO and the best competing model using t-test.

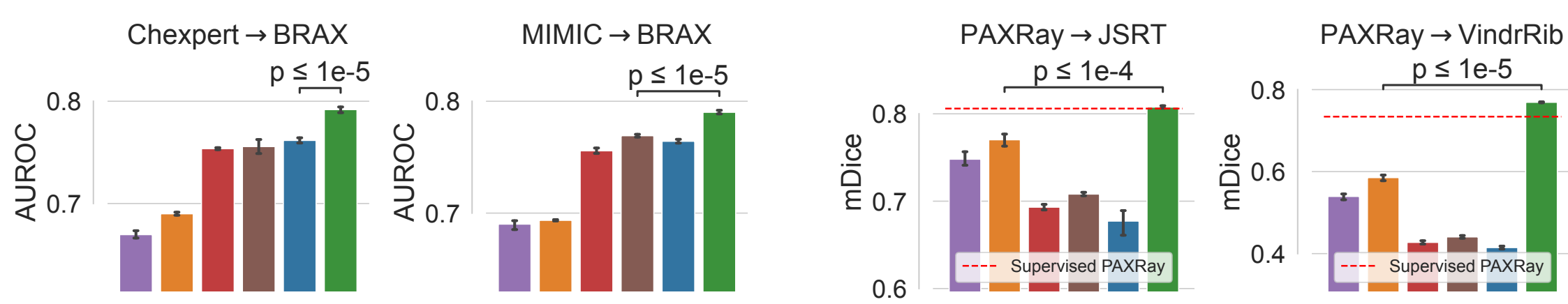

Supplementary Fig. 36. Comparison for classification and segmentation generalization performances across 5 seeds. Statistical comparison between RayDINO and the best competing model using t-test.

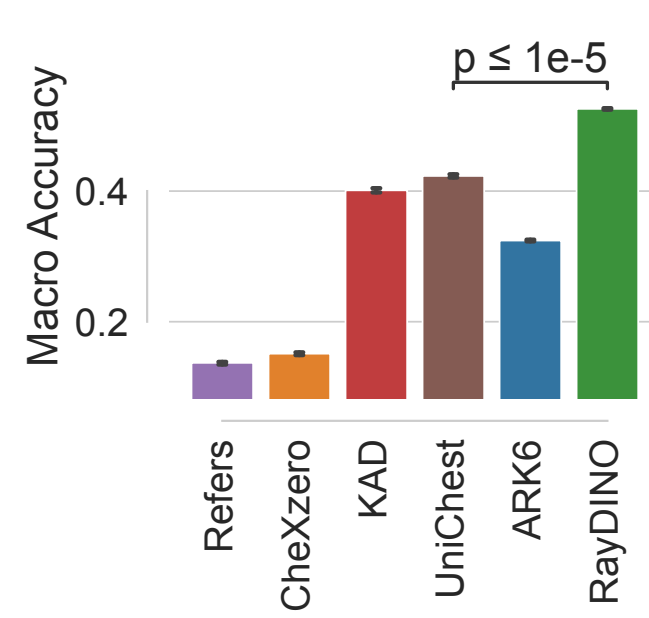

Supplementary Fig. 37. Comparison for attention pin-pointing (interpretability) performances across 5 seeds. Statistical comparison between RayDINO and the best competing model using t-test.

Supplementary Tables

Model	Bleu 4	Rouge-L	RadGraph F1	CheXbert score	RadCliQ $\downarrow$	Macro-F1-14	Micro-F1-14
MAIRA-1†	14.2 [13.7,14.7]	28.9 [28.4,29.4]	24.3 [23.7,24.8]	44.0 [43.1,44.9]	3.10 [3.07,3.14]	38.6 [37.1,40.1]	55.7 [54.7,56.8]
MAIRA-1	**14.0** [13.6,14.5]	28.5 [28.0,28.9]	22.8 [22.1,23.3]	42.8 [42.0,43.6]	3.16 [3.13,3.19]	**36.7** [35.0,38.2]	54.3 [53.3,55.4]
Refers	10.8 [10.5,11.3]	27.3 [26.6,27.9]	19.8 [19.2,20.5]	36.7 [35.9,37.5]	3.36 [3.33,3.40]	23.2 [22.0,24.6]	40.1 [38.6,41.3]
CheXzero	10.5 [10.2,10.9]	27.6 [27.0,28.1]	20.5 [19.9,21.0]	38.8 [37.9,39.7]	3.30 [3.27,3.34]	26.4 [24.9,27.8]	43.3 [42.1,44.6]
KAD	12.7 [12.3,13.1]	29.6 [29.0,30.2]	23.0 [22.4,23.7]	42.8 [42.0,43.7]	3.13 [3.09,3.17]	32.5 [30.9,34.1]	50.2 [48.8,51.4]
UniChest	13.0 [12.5,13.4]	29.7 [29.1,30.3]	23.5 [23.0,24.2]	43.6 [42.8,44.5]	3.10 [3.07,3.14]	32.5 [30.9,34.1]	52.4 [51.1,53.6]
ARK6	12.5 [12.0,12.9]	29.3 [28.8,30.0]	22.8 [22.1,23.4]	42.8 [42.0,43.7]	3.14 [3.10,3.18]	32.2 [30.6,33.5]	51.1 [49.8,52.1]
RayDINO	13.8 [13.4,14.2]	**30.3** [29.7,30.8]	**23.9** [23.3,24.6]	**44.8** [44.0,45.6]	**3.07** [3.04,3.11]	36.1 [34.4,37.6]	**54.8** [53.5,56.0]

Supplementary Table 1. Report generation results:. We report median and 95% confidence intervals based on 500 bootstrap samples from the MIMIC-CXR test set. † trained on GPT-4 augmented reports. RayDINO performs on par or significantly better than all competitors on all metrics.

	Split	Female (%)	Age	No Finding (%)
Mimic	Young	57.4	27.1 (5.0)	68.8
	Middle	49.0	50.1 (6.8)	55.6
	Elder	52.9	74.6 (9.0)	34.6
NIH	Female	100.0	46.8 (16.3)	49.2
	Male	0.0	46.9 (17.3)	58.2
ChX.	Female	100.0	62.4 (19.0)	22.5
	Male	0.0	59.5 (18.4)	34.3

Supplementary Table 2. Split statistics for bias analysis.
