# Supplementary material for: Advancing human-centric AI for robust X-ray analysis through holistic self-supervised learning
Source: Nat Commun. 2026 Aug 20;17:9931. doi: 10.1038/s41467-026-76076-4 (PMC13586212; doi:10.1038/s41467-026-76076-4)
Supplement: Supplementary file 2 [file 41467_2026_76076_MOESM2_ESM.pdf]

nature portfolio

Corresponding author(s): Théo Moutakanni

Last updated by author(s): 29/05/2026

Reporting Summary

Nature Portfolio wishes to improve the reproducibility of the work that we publish. This form provides structure for consistency and transparency in reporting. For further information on Nature Portfolio policies, see our Editorial Policies and the Editorial Policy Checklist.

Statistics

For all statistical analyses, confirm that the following items are present in the figure legend, table legend, main text, or Methods section.

n/a	Confirmed	
☐	☒	The exact sample size (*n*) for each experimental group/condition, given as a discrete number and unit of measurement
☐	☒	A statement on whether measurements were taken from distinct samples or whether the same sample was measured repeatedly
☐	☒	The statistical test(s) used AND whether they are one- or two-sided<br>*Only common tests should be described solely by name; describe more complex techniques in the Methods section.*
☐	☒	A description of all covariates tested
☐	☒	A description of any assumptions or corrections, such as tests of normality and adjustment for multiple comparisons
☐	☒	A full description of the statistical parameters including central tendency (e.g. means) or other basic estimates (e.g. regression coefficient) AND variation (e.g. standard deviation) or associated estimates of uncertainty (e.g. confidence intervals)
☐	☒	For null hypothesis testing, the test statistic (e.g. *F*, *t*, *r*) with confidence intervals, effect sizes, degrees of freedom and *P* value noted<br>*Give P values as exact values whenever suitable.*
☒	☐	For Bayesian analysis, information on the choice of priors and Markov chain Monte Carlo settings
☒	☐	For hierarchical and complex designs, identification of the appropriate level for tests and full reporting of outcomes
☐	☒	Estimates of effect sizes (e.g. Cohen's *d*, Pearson's *r*), indicating how they were calculated

Our web collection on statistics for biologists contains articles on many of the points above.

Software and code

Policy information about availability of computer code

Data collection	No data collection have been made. We used publicly available data from multiple institutions, as stated in the manuscript.
Data analysis	All models were implemented in Python (v3.10) using PyTorch (v2.1). Self-supervised pretraining used the open-source DINOv2 codebase (https://github.com/facebookresearch/dinov2). Segmentation heads were implemented using mmsegmentation (v1.2). Report generation used the Llama-2-7B language model54. Evaluation metrics for report generation were computed using pycocoevalcap (v1.2), RadGraph58, CheXbert59, and the RadCliQ reference implementation57. Statistical tests were performed using scipy (v1.11). Data preprocessing and analysis used NumPy (v1.24) and pandas (v2.0). All software versions should be verified against the released code repository.

For manuscripts utilizing custom algorithms or software that are central to the research but not yet described in published literature, software must be made available to editors and reviewers. We strongly encourage code deposition in a community repository (e.g. GitHub). See the Nature Portfolio guidelines for submitting code & software for further information.

Data

Policy information about availability of data

All manuscripts must include a data availability statement. This statement should provide the following information, where applicable:

- Accession codes, unique identifiers, or web links for publicly available datasets
- A description of any restrictions on data availability
- For clinical datasets or third party data, please ensure that the statement adheres to our policy

All data are available in the manuscript or the supplementary materials.
Code availability Training code available at https://github.com/facebookresearch/dinov2. RayDINO model is available at https://ai.meta.com/resources/models-and-libraries/raydino-downloads/

Research involving human participants, their data, or biological material

Policy information about studies with human participants or human data. See also policy information about sex, gender (identity/presentation), and sexual orientation and race, ethnicity and racism.

Reporting on sex and gender	When designing sex related analysis, we used the sex category made available by the CheXpert and the NIH datasets.
Reporting on race, ethnicity, or other socially relevant groupings	The countries of origin of the hospitals providing the images were used to evaluate the bias of benchmarked AI models. We used the reporting of the dataset providers to get them.
Population characteristics	We analyzed age as a covariate relevant to our study.
Recruitment	We didn't perform recruitment as the data are originating from external public sources. We selected all the biggest dataset available to us, with a focus on maximizing diversity (eg. country, labels and hospitals).
Ethics oversight	NA

Note that full information on the approval of the study protocol must also be provided in the manuscript.

Field-specific reporting

Please select the one below that is the best fit for your research. If you are not sure, read the appropriate sections before making your selection.

[x] Life sciences [ ] Behavioural & social sciences [ ] Ecological, evolutionary & environmental sciences

For a reference copy of the document with all sections, see nature.com/documents/nr-reporting-summary-flat.pdf

Life sciences study design

All studies must disclose on these points even when the disclosure is negative.

Sample size	Given the cost of training AI models, we picked 5 samples for each evaluations of each models and 20 per population for the covariate analysis as it has higher variance.<br>Most of the cited literature in this field report 1 to 5 samples per evaluated model, and given the low variance of our reported results, we think that 5 is large enough to make good conclusions.
Data exclusions	No data were excluded.
Replication	We repeated all evaluation trainings 5 times and report mean and std. The performances reached by all benchmarked models are stable across different run and thus can be repeated.
Randomization	Train, validation and test sets are created following the specification in the manuscript (either by following the same splits as found in the literature to increase reproducibility, or by random sampling if they were not available). When sampling randomly, we made sure to not include the same patient in different sets (train/val/test). Covariate-specific settings are stated in our manuscript (eg.
Blinding	NA

Reporting for specific materials, systems and methods

We require information from authors about some types of materials, experimental systems and methods used in many studies. Here, indicate whether each material, system or method listed is relevant to your study. If you are not sure if a list item applies to your research, read the appropriate section before selecting a response.

Materials & experimental systems

n/a	Involved in the study
☒	☐ Antibodies
☒	☐ Eukaryotic cell lines
☒	☐ Palaeontology and archaeology
☒	☐ Animals and other organisms
☒	☐ Clinical data
☒	☐ Dual use research of concern
☒	☐ Plants

Methods

n/a	Involved in the study
☒	☐ ChIP-seq
☒	☐ Flow cytometry
☒	☐ MRI-based neuroimaging

Plants

Seed stocks: na

Novel plant genotypes: na

Authentication: na
