# Supplementary material for: Advancing human-centric AI for robust X-ray analysis through holistic self-supervised learning
Source: Nat Commun. 2026 Aug 20;17:9931. doi: 10.1038/s41467-026-76076-4 (PMC13586212; doi:10.1038/s41467-026-76076-4)
Supplement: Supplementary file 3 [file 41467_2026_76076_MOESM3_ESM.pdf]

nature portfolio

Peer Review File

Advancing human-centric AI for robust X-ray analysis through holistic self-supervised learning

Corresponding Author: Mr Théo Moutakanni

This file contains all reviewer reports in order by version, followed by all author rebuttals in order by version.

Version 0:

Reviewer comments:

Reviewer #1

(Remarks to the Author)
Content:

The paper evaluates the performance of a foundation model when tested on a large variety of radiologist tasks, aiming to generalize properly on new data, remove biases, and provide detailed information about the population tested.

The results show that the approach can be effective and allow to build strong tools to help medical doctors. However, I have some concerns about the experimental setup, especially on the comparison with benchmark algorithms.

Comments / suggestions:
- The abstract can include the number of datasets (11) and new samples tested, helping to catch the attention of a reader and increase the impact.
- ‘providing separate AI models for each organ and anomaly visible in chest X-rays is impractical and can be unfeasible’. I completely agree on the infeasibility, but usually medical doctors are specialized on a specific domain of medicine that can involve a single organ. I would maybe remove ‘impractical’, stressing the fact that it involves a lot of manual work that does not scale with the amount of new medical data (as partially mentioned in the following sentence).- The introduction can be smoother, better linking some sentences (such as ‘Recent studies have raised concerns about the quality of automatic text-based data labeling and the reliability of AI systems trained on reports from medical images’, ‘). Recommend revising to improve readability for the less informed reader.
- The paragraph in the introduction where RayDINO is described should be restructured. As presented, first, the experimental setup is described, then how the model is analyzed, then again, the experimental setup (the datasets, the benchmarks).
- Results section – 1st paragraph - Elaborate further on how RayDINO was compared with benchmarks.
- ‘Furthermore, despite using 100x less supervised parameters’ 100x less supervised parameters compared to what?
- How can RayDINO be multimodal? In Figure 1 it looks like it has been trained just with images.
- The comparison is sometimes hard to understand. Can you please report your result, the benchmark result and the difference in performance, instead of your result and the difference in performance only?
- The results notation is sometimes fuzzy, especially when the improvement is limited (e.g. +0.9 over UniChest on rare diseases). Could you report the performance metrics between 0 and 1?
- The division between age groups (Section Examining Performance Across Sex and Age Groups) can be misleading: a kid shows a completely different chest morphology compared with a person of 20 years old, while instead a 35-year-old person is more similar to 50 years old. Would it be possible to repeat the experiment, using 0-12, 12-35, 35-60, 60-100 as groups?
- ‘Explainability. I would change the title to ‘Interpretability’ since Explainability is a slightly different domain (focuses on how a model produces results to justify decisions) and what you are proposing exploits interpretability (investigates the algorithm logic, helping to make algorithm components comprehensible to humans). Furthermore, interpretability and explainability are both used in the text. I would just use interpretability.
- Data section within methods is a bit light. I think this section should be restructured, maybe adding a table showing data distribution and better describing their characteristics (machine used to acquired data, origin) to highlight the heterogeneity and make your paper stronger. Also on the downstream task, it would be nice to see how data are used and to better highlight how the classifier is finetuned (I guess just training the layers after the image encoder).
- The report generation is not clear to me. The text states that only images are used during the training, therefore it sounds

strange that the feature space of images is perfectly aligned with the one of reports, so that image features can feed a LLM without any other modification. Can you please better describe the report generation?
- How are the data used to pre-train the model? How are they split in training, validation, testing later?
- I have a doubt about the result: a single value is reported for every task analyze. Did you train a single model? Are you reporting the best model among multiple models? This detail must be clarified. I can imagine you trained just a single backbone with SSL, due to the massive amount of data. What about the downstream tasks?
- I have some doubts about the comparisons: how did you compare the benchmark algorithms? Did you use the same data to pre-train and fine-tune? Can you please better clarify this detail, since the paper is based on this comparison, but it is not clear to me.
-

(Remarks on code availability)
We have used DINOv2 in some of our work. However, the article is about RayDINO which is specifically for chest X-ray images. DINOv2 is (mostly) on general purpose images but may contain some medical images.

Reviewer #2

(Remarks to the Author)
I co-reviewed this manuscript with one of the reviewers who provided the listed reports. This is part of the Nature Communications initiative to facilitate training in peer review and to provide appropriate recognition for Early Career Researchers who co-review manuscripts.

(Remarks on code availability)

Reviewer #3

(Remarks to the Author)
Thanks for this impressive and fascinating manuscript. I will break comments in to larger issues and smaller issues. Just to be clear, I am a clinician scientist who continues to practice medicine, yet have a reasonable understanding of AI methods and techniques and have used them for my own research.
#1) The first issue is that the manuscript is overflowing with methods, technics and results, but actually far too many to be able to sufficiently explain what was done and how to interpret them. This looks more like a compressed thesis than a research manuscript. In the effort to squeeze this all into a manuscript with a limited number of words, much of the detail needed for the readers to trust and understand the tool are missing. More explicit examples of the are as follows;

Unclear Radiology Tasks: The authors mention nine radiology tasks, but they fail to clarify what these tasks entail. They categorize them into three broad categories: classification, segmentation, and report generation, but specific details about each of the nine tasks are missing. For example, in the classification tasks, it is not clear what exactly is being classified.

Missing Data Processing Explanation: There's no mention of how the authors process their data, which is a crucial part of any machine learning pipeline. Data preprocessing can heavily influence the results, and without this information, it’s hard to replicate or trust their findings.
Lack of Clear Presentation Despite Substantial Effort: While the paper reflects considerable work, the presentation is insufficient to convey the depth of their efforts. The authors introduce various techniques and tasks but don't explain them in detail, making it hard for readers to follow along. If the authors broke down their methodology and results more thoroughly, it would significantly improve the clarity and perceived quality of their work.

The authors frequently use terms like "foundation models" and "self-supervised learning" but fail to provide in-depth explanations or examples of how these concepts are applied in their specific work. It would be a very small group of readers that really fully understand all the terms used. One of the reasons for the minimal explanation is the massive amount of work that is being introduced, so it is clear that better explanations would require a significant amount of words/space.

#2) Another significant issue is the tone of the manuscript. This is ultimately an editorial issue, but the tone struck me as inappropriate for a scientific journal. It is very possible that all the superlatives used may turn out to be fair for such a potentially useful tool, but one it not used to this tone. Also, while the text seems to heavily promote the tool, the paper lacks a clear description of how why and how it is better. Even if it is really as good as advertised, the proof is in the details, which are not given enough explanation. Some examples;

ABSTRACT
“state-of-the-art results”
-no specific meaning of state of the art
INTRODUCTION
“RayDINO surpasses other task-specific supervised models”
-This comment is made in the introduction without any references, additionally, surpasses is non-specific. If it has previously been shown to have a better performance in a task, name the task and the reference.
“As part of our extensive quantitative evaluation,”
-can leave out “extensive””

DISCUSSION
“Impressive performances with respect to other foundation methods and the added value of RayDINO.”
-Can leave out “impressive”

#3) Some evaluation on Small Datasets: In Figure 1B, the authors mention evaluating their model using various datasets such as JSRT (154 images), Scoliosis (609 images), and VinDr Rib (245 images). These datasets are relatively small for evaluating deep learning models, which typically require larger datasets to demonstrate robustness and generalization. The small sample sizes call into question the validity of their evaluation and whether their model can generalize effectively to broader clinical settings.

#4) The role of Meta needs to be clarified. Meta is not listed as a source of funding for the study which is not possible as it's employees worked on the study. The following needs to be clarified;

-What did Meta contribute to the study?
-Is RayDino a Meta product?
-What was the role of Meta in writing the manuscript?

(Remarks on code availability)
The readme file was available and clear. The yaml file cold not be loaded and therefore the code could not be reviewed.

Reviewer #4

(Remarks to the Author)
I co-reviewed this manuscript with one of the reviewers who provided the listed reports. This is part of the Nature Communications initiative to facilitate training in peer review and to provide appropriate recognition for Early Career Researchers who co-review manuscripts.

(Remarks on code availability)

Version 1:

Reviewer comments:

Reviewer #1

(Remarks to the Author)
The authors have adequately addressed the concerns raised in the previous review cycle. I have no further comments to make.

(Remarks on code availability)
Cursorily reviewed code. Seems OK. Did not run the code or attempt to replicate results which is a significantly involved process.

Reviewer #2

(Remarks to the Author)
I co-reviewed this manuscript with one of the reviewers who provided the listed reports. This is part of the Nature Communications initiative to facilitate training in peer review and to provide appropriate recognition for Early Career Researchers who co-review manuscripts.

(Remarks on code availability)
I think the paper improved a lot from the last revision, here is some minor comments.

- L36: “Our model generates holistic representations of a compact size of 1024 for each…” it is clear from the context, but somebody outside of computer science domain may not understand what 1024 refers to.
- L84: I would better specify that other compared models are fully-supervised and so their parameters are optimized on that specific task.
- I would maybe better stress the role of the adapter between images and text. As far as it is written, text and images are not aligned, which makes the result even more impressive. Did you verify the presence of hallucinations in the report generation?

Reviewer #3

(Remarks to the Author)
Regarding the manuscript in general, some has improved, but a lot has been added, some of which needs to get cleaned up. The introduction is full of methods and results. Figure 1 and its explanation are in the Introduction and includes both methods and results. The strategy of the loss functions is also in the introduction.

The “conclusion” is now in the introduction, “Our findings indicate that RayDINO’s representation provides state-of-the-art performance on all 21 benchmarks by surpassing the best available chest X-ray foundation models in the literature on most of them (Fig. 1d), without relying on human annotations during its pre-training phase.” This is a conclusion based on the results. It should not be in the introduction. Especially the comment that it surpasses the other foundation models, without any references to the other models.

It looks like comparative hypothesis testing was done in a very unusual way. First it looks like a t-test was used to compare summary statistics. The t-test is only designed for specific uses, not to compare all summary statistics. The t-test was not designed to compare AUCs for instance, not Pearson’s correlation coefficients. Additionally, it looks like some type of bootstrapping was done by repeating a measurement only done 5 times. This needs to be clarified with the exact process used and why it was only repeated 5 times, other than a typical number of at least 1000 for the larger datasets.

Regarding my prior issues, most have been addressed at least partially.

In relation to my issues with the small data sets, I was not implying that they cannot be used for testing, but a comparison between models would be difficult. For example, the JSRT has 154 images, while the precision of the mDice for RayDino is .960 [0.959-.960]. This suggests an error of less than 0.001 in a sample of 154. This seems highly unlikely and is much smaller than errors that I have seen in other studies using mDice. This may be related to the confidence interval method noted above which I question.

Lastly I am happy to hear that Meta was listed as providing computer resources. I do not understand why Meta is allowing its employees to work on this project in their computing environment. I have no reason to think that this is necessarily a problem, but I think that it would be best to just list this as a competing interest as the code also sits in the Meta domain

(Remarks on code availability)

Version 2:

Reviewer comments:

Reviewer #3

(Remarks to the Author)

The version is much improved and I appreciate your attention to the previously noted issues. I do not have any further issues with this manuscript.

(Remarks on code availability)

Open Access This Peer Review File is licensed under a Creative Commons Attribution 4.0 International License, which permits use, sharing, adaptation, distribution and reproduction in any medium or format, as long as you give appropriate credit to the original author(s) and the source, provide a link to the Creative Commons license, and indicate if changes were made.

In cases where reviewers are anonymous, credit should be given to 'Anonymous Referee' and the source.

The images or other third party material in this Peer Review File are included in the article's Creative Commons license, unless indicated otherwise in a credit line to the material. If material is not included in the article's Creative Commons license and your intended use is not permitted by statutory regulation or exceeds the permitted use, you will need to obtain permission directly from the copyright holder.
To view a copy of this license, visit https://creativecommons.org/licenses/by/4.0/

Nature NCOMMS-24-52109A - Response to Reviewers

First of all, we would like to thank all the reviewers and the editor for the thorough and insightful feedback on our manuscript. Your constructive comments have been instrumental in enhancing the quality and depth of our work. In response to your valuable suggestions, we have made extensive revisions throughout the manuscript. All modifications have been highlighted in red in the draft, we also added Table 1,2 and Figure 5 to describe in more details the different datasets used in this study, Table 3 to present an overview of the compared models and task specific adapters used for RayDINO and improved Figure 1. We believe that these revisions have significantly strengthened the manuscript.

New or Updated Figures had been added in the new version of the manuscript including:
P2 Figure 1:

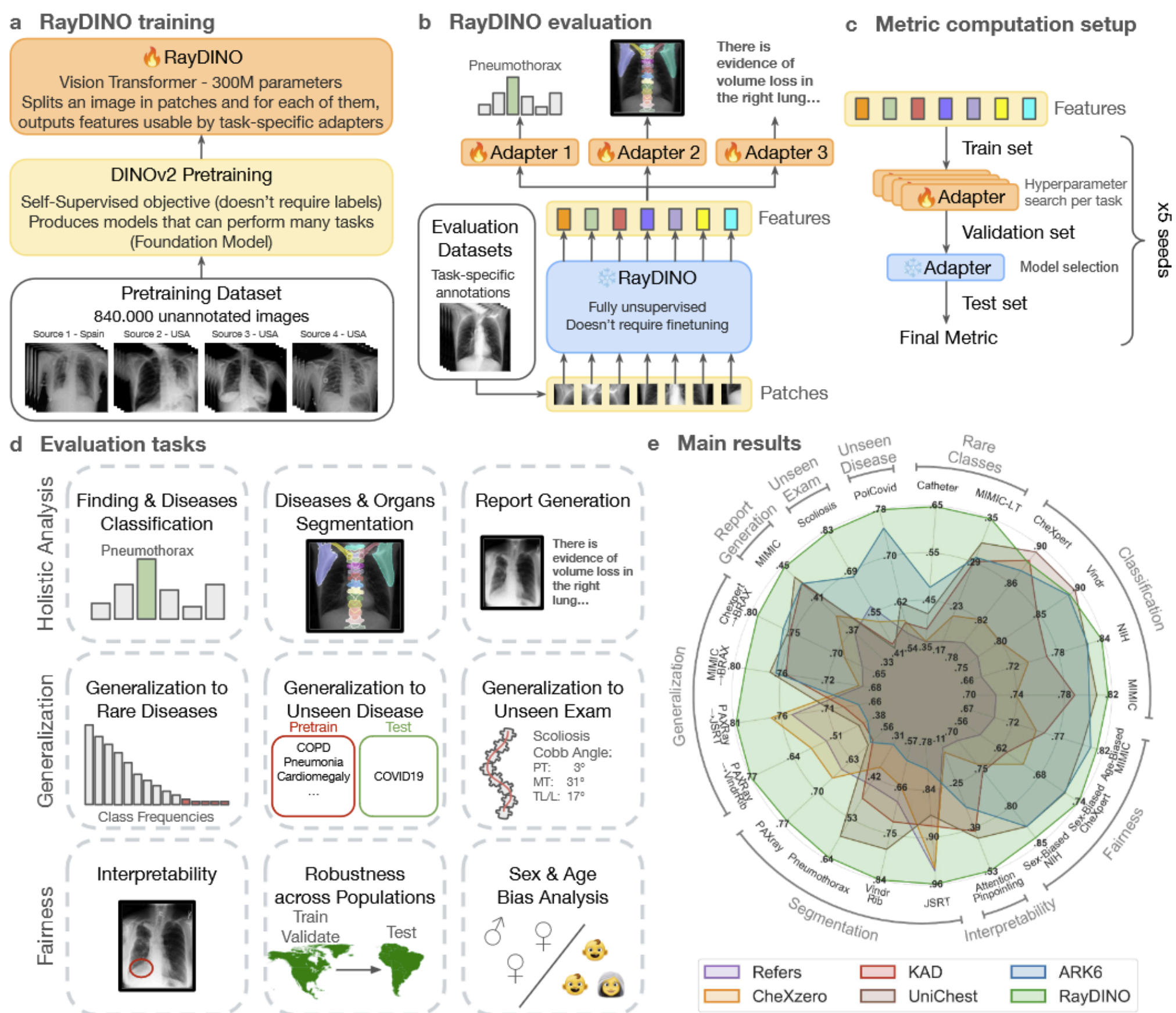

Figure 1. We introduce RayDINO, a foundation model for holistic and fairer analysis of chest X-rays. a) RayDINO is a vision transformer trained using DINOv2 objectives on four different datasets from the USA and Europe. **b)** We evaluate RayDINO and other models as-is on all tasks without any modification or specialization of their parameters by using very small task-specific adapters. **c)** Our supervised adapters are trained and validated for each task using a hyperparameters search. We repeated this setup 5 times to produce confidence intervals and compare models. **d)** RayDINO is evaluated on tasks divided into three categories. **e)** RayDINO consistently delivers excellent performance on 21 benchmarks (AUROC for Classification and Fairness, macro Accuracy for interpretability, mDice for Segmentation, AUROC for BRAX Generalization, and mDice for PAXRay Generalization, CheXbert vector similarity for Report Generation, Pearson's correlation for Unseen Exam, macro Accuracy for Unseen Disease, AUPRC/Area Under Precision Recall Curve for Rare Classes).

P12 Figure 5:

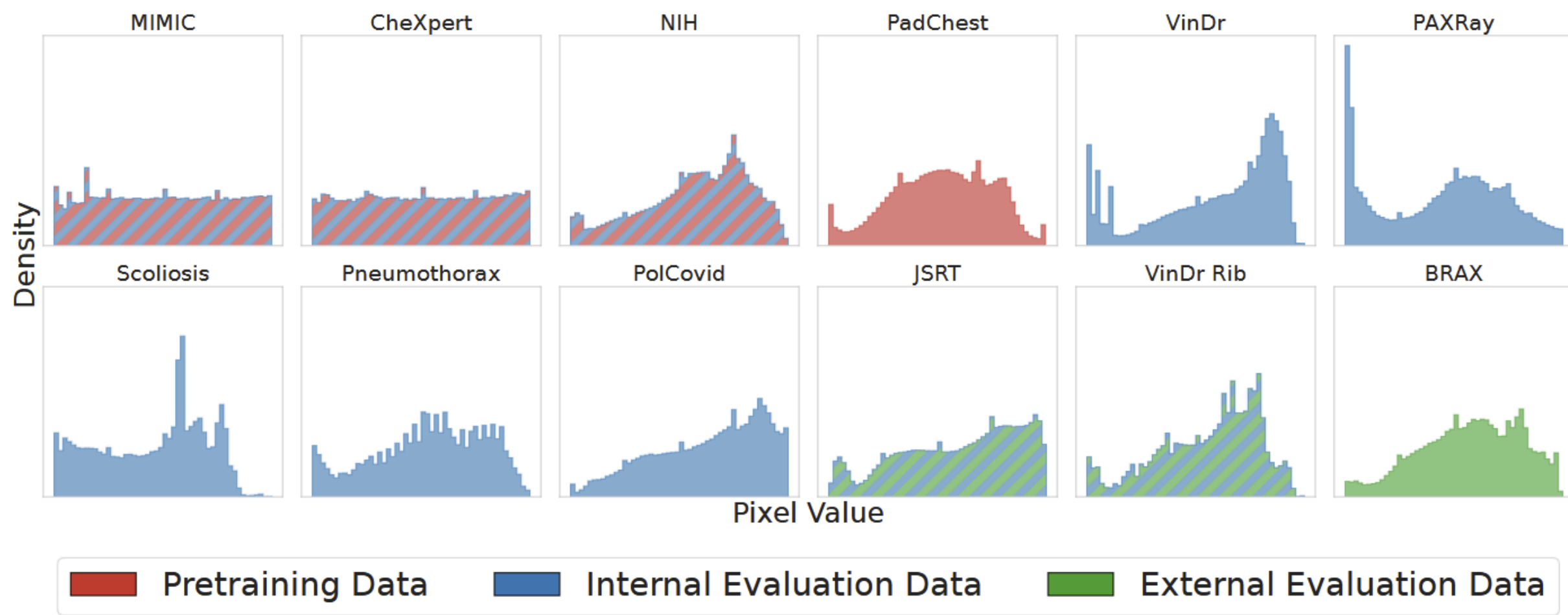

Figure 5. **Non-zero pixel values** histogram for each dataset in this study. For each histogram we indicate with different colors their use to pretraining, internal and external evaluation datasets.

New Tables:

P10 Table 1:

Name	Used For	Train	Val	Test	Country	Type	Labels	# Label	% Female	Avg. Age	% Healthy
Pretraining Datasets											
PadChest	Pretrain	160.845	-	-	Spain	-	-	-	49.6	58.6	31.4
CheXpert	Pre. & Int.	223.414	234	668	USA	Classif.	Findings	14	40.7	60.6	10.0
MIMIC	Pre. & Int.	368.960	2.991	5.159	USA	Classif.	Findings	14	47.5	61.6	38.3
NIH	Pre. & Int.	83.524	3.000	25.596	USA	Classif.	Findings	15	44.0	46.6	58.4
Internal Evaluation Datasets											
VinDr	Internal	14.000	1.000	3.000	Vietnam	Classif.	Findings	28	-	-	70.7
PAXRay++	Internal	11.802	1.475	1.476	Multiple	Segm.	Bones & Organs	159	-	-	-
Scoliosis	Internal	481	-	128	Canada	Reg.	Spine angles	3	-	-	-
Pneumothorax	Internal	10.675	1.372	3.205	USA	Segm.	Diseases	2	-	-	77.7
POLCOVID	Internal	2.985	509	1.315	Poland	Classif.	Diseases	3	47.4	60.2	65.8
Catheter	Internal	24.066	3.008	3.009	USA	Classif.	Catheter Pos.	9	-	-	-
MIMIC-LongTail	Internal	264.849	36.769	75.492	USA	Classif.	Findings	40	47.5	61.6	38.3
Report Generation	Internal	267.215	2.114	3.822	USA	Text Gen.	Radiological Reports	-	48.6	61.3	46.4
External Evaluation Datasets											
JSRT	Int. & Ext.	197	-	50	Japan	Segm.	Organs	5	52.8	58.2	-
VinDr Rib	Int. & Ext.	196	-	49	Vietnam	Segm.	Ribs	20	-	-	-
BRAX	External	-	-	40.967	Brasil	Classif.	Findings	14	50.6	46.0	70.8

Table 1. **Overview of all the datasets used in this study.** They are grouped in 3 categories: pre-training of RayDINO, internal evaluation set (downstream adapter trained on them), or external evaluation set (adapter not trained on them). One dataset can be used in multiple setups. The number of exams used for the training, validation and test splits for each dataset is presented together with information about the country of origin, the type of benchmark and labels as well as the % of female and healthy exams and the average age.

P11 Table 2:

CheXpert	Atelectasis, Cardiomegaly, Consolidation, Edema, Enlarged Cardiomediastinum, Fracture, Lung Lesion, Lung Opacity, No Finding, Pleural Effusion, Pleural Other, Pneumonia, Pneumothorax, Support Devices
MIMIC	Atelectasis, Cardiomegaly, Consolidation, Edema, Enlarged Cardiomediastinum, Fracture, Lung Lesion, Lung Opacity, No Finding, Pleural Effusion, Pleural Other, Pneumonia, Pneumothorax, Support Devices
NIH	Atelectasis, Cardiomegaly, Consolidation, Edema, Effusion, Emphysema, Fibrosis, Hernia, Infiltration, Mass, No Finding, Nodule, Pleural Thickening, Pneumonia, Pneumothorax
VinDr	Aortic Enlargement, Atelectasis, Calcification, Cardiomegaly, Clavicle Fracture, Consolidation, Copd, Edema, Emphysema, Enlarged Pa, Ild, Infiltration, Lung Cavity, Lung Cyst, Lung Opacity, Lung Tumor, Mediastinal Shift, No Finding, Nodule Mass, Other Disease, Other Lesion, Pleural Effusion, Pleural Thickening, Pneumonia, Pneumothorax, Pulmonary Fibrosis, Rib Fracture, Tuberculosis
PaxRAY++	Spine, Cervical Spine, Thoracic Spine, Lumbar Spine, Vertebrae C1, C2, C3, C4, C5, C6, C7, Vertebrae T1, T2, T3, T4, T5, T6, T7, T8, T9, T10, T11, T12, Vertebrae L1, L2, L3, L4, L5, Rib Cartilage, Sternum, Clavicles, Clavicle Left, Clavicle Right, Scapulas, Scapula Left, Scapula Right, Posterior Rib R1, R2, R3, R4, R5, R6, R7, R8, R9, R10, R11, R12, Anterior Rib R1, R2, R3, R4, R5, R6, R7, R8, R9, R10, R11, Posterior Rib L1, L2, L3, L4, L5, L6, L7, L8, L9, L10, L11, L12, Anterior Rib L1, L2, L3, L4, L5, L6, L7, L8, L9, L10, L11, Diaphragm, Left Hemidiaphragm, Right Hemidiaphragm, Stomach, Small Bowel, Duodenum, Liver, Pancreas, Kidney Left, Kidney Right, Cardiomediastinum, Upper Mediastinum, Lower Mediastinum, Anterior Mediastinum, Middle Mediastinum, Posterior Mediastinum, Heart, Heart Atrium Left, Heart Atrium Right, Heart Myocardium, Heart Ventricle Left, Heart Ventricle Right, Aorta, Ascending Aorta, Descending Aorta, Aortic Arch, Pulmonary Artery, Inferior Vena Cava, Esophagus, Lung, Right Lung, Left Lung, Lung Base, Mid Zone Lung, Upper Zone Lung, Apical Zone Lung, Right Upper Zone Lung, Right Mid Zone Lung, Right Lung Base, Right Apical Zone Lung, Left Upper Zone Lung, Left Mid Zone Lung, Left Lung Base, Left Apical Zone Lung, Lung Lower Lobe Left, Lung Upper Lobe Left, Lung Lower Lobe Right, Lung Middle Lobe Right, Lung Upper Lobe Right, Trachea, Tracheal Bifurcation, Breast, Breast Left, Breast Right
Scoliosis	MT Angle, PT Angle, TL-L Angle
Pneumothorax	Normal, Pneumothorax
POLCOVID	Covid, Normal, Pneumonia
JSRT	Left Lung, Right Lung, Left Clavicle, Right Clavicle, Heart
VinDr Rib	Rib R1, R2, R3, R4, R5, R6, R7, R8, R9, R10, L1, L2, L3, L4, L5, L6, L7, L8, L9, L10
BRAX	Atelectasis, Cardiomegaly, Consolidation, Edema, Enlarged Cardiomediastinum, Fracture, Lung Lesion, Lung Opacity, No Finding, Pleural Effusion, Pleural Other, Pneumonia, Pneumothorax, Support Devices
Catheter	Ett Abnormal, Ett Borderline, Ett Normal, Ngt Abnormal, Ngt Borderline, Ngt Normal, Cvc Abnormal, Cvc Borderline, Cvc Normal
MIMIC-LT	Adenopathy, Atelectasis, Azygos Lobe, Calcification Of The Aorta, Cardiomegaly, Clavicle Fracture, Consolidation, Edema, Emphysema, Enlarged Cardiomediastinum, Fibrosis, Fissure, Fracture, Granuloma, Hernia, Hydropneumothorax, Infarction, Infiltration, Kyphosis, Lobar Atelectasis, Lung Lesion, Lung Opacity, Mass, Nodule, Normal, Pleural Effusion, Pleural Other, Pleural Thickening, Pneumomediastinum, Pneumonia, Pneumoperitoneum, Pneumothorax, Pulmonary Embolism, Pulmonary Hypertension, Rib Fracture, Rounded Atelectasis, Subcutaneous Emphysema, Support Devices, Tortuous Aorta, Tuberculosis

Table 2. Overview of the different datasets and their detailed classes used in our study. The table presents a clear list of all the benchmarked X-Ray anomalies and anatomies, showing the extensive evaluation of RayDINO.

P13 Table 3:

Model	Architecture	Num. Params	Pre-Train Datasets
Benchmarked Backbones			
UniChest[37]	ResNet50	55 M	CheXpert, MIMIC, NIH, VinDr
KAD[36]	ResNet50	55 M	MIMIC
ARK6[38]	Swin-B	91 M	CheXpert, MIMIC, NIH, VinDr, Pneumonia, Shenzhen
CheXzero[35]	ViT-B	86 M	MIMIC
Refers[34]	ViT-B	86 M	MIMIC
RayDINO (ours)	ViT-L	307 M	CheXpert, MIMIC, NIH, PadChest
Task-Specific Adapters			
Classification / Regression Adapter	2 layers MLP + ReLU	0.1-1 M	-
Segmentation Adapter	UperNet[52]	49 M	-
Text Adapter	LLM (Llama[53])	7 B	-

Table 3. **Parameter size** of the different backbones we use in this study along with the task-specific adapters.

REVIEWERS #1 and #2

Content: The paper evaluates the performance of a foundation model when tested on a large variety of radiologist tasks, aiming to generalize properly on new data, remove biases, and provide detailed information about the population tested. The results show that the approach can be effective and allow to build strong tools to help medical doctors. However, I have some concerns about the experimental setup, especially on the comparison with benchmark algorithms.

Response

Thank you for your valuable feedback and the recognition of our results that could help medical experts. In response to your concerns, we have significantly reworked parts of our manuscript, including the experimental setup and the comparisons of our models.

1. *The abstract can include the number of datasets (11) and new samples tested, helping to catch the attention of a reader and increase the impact.*

   Response

   Thank you very much for your recommendation to ameliorate the readability of our manuscript. We have updated the description of our model in the abstract, including the number of datasets and samples that were used for the training of our models. Moreover, we have updated the introduction section with more details about our benchmarks and the dataset used, while in the online methods Tables 1, 2 give an overview of all the datasets used in our study.

   P1 L12 Abstract: "We introduce RayDINO, a large-scale self-supervised encoder for chest X-rays. It was trained on 840,000 images and evaluated on 82,000 images sourced from 12 publicly available datasets."

   P2 L43 Introduction: "We evaluate its performance by using task-specific adapters on top of frozen RayDINO features (Fig. 1b,c) using eleven publicly available datasets from seven countries across four continents, both in internal and external settings. We report results from nine medical-imaging related tasks (Fig. 1d) divided into 21 benchmarks comprising more than 200 classes (Fig. 1e)."

   P10 Table 1

Name	Used For	Train	Val	Test	Country	Type	Labels	# Label	% Female	Avg. Age	% Healthy
Pretraining Datasets											
PadChest	Pretrain	160.845	-	-	Spain	-	-	-	49.6	58.6	31.4
CheXpert	Pre. & Int.	223.414	234	668	USA	Classif.	Findings	14	40.7	60.6	10.0
MIMIC	Pre. & Int.	368.960	2.991	5.159	USA	Classif.	Findings	14	47.5	61.6	38.3
NIH	Pre. & Int.	83.524	3.000	25.596	USA	Classif.	Findings	15	44.0	46.6	58.4
Internal Evaluation Datasets											
VinDr	Internal	14.000	1.000	3.000	Vietnam	Classif.	Findings	28	-	-	70.7
PAXRay++	Internal	11.802	1.475	1.476	Multiple	Segm.	Bones & Organs	159	-	-	-
Scoliosis	Internal	481	-	128	Canada	Reg.	Spine angles	3	-	-	-
Pneumothorax	Internal	10.675	1.372	3.205	USA	Segm.	Diseases	2	-	-	77.7
POLCOVID	Internal	2.985	509	1.315	Poland	Classif.	Diseases	3	47.4	60.2	65.8
Catheter	Internal	24.066	3.008	3.009	USA	Classif.	Catheter Pos.	9	-	-	-
MIMIC-LongTail	Internal	264.849	36.769	75.492	USA	Classif.	Findings	40	47.5	61.6	38.3
Report Generation	Internal	267.215	2.114	3.822	USA	Text Gen.	Radiological Reports	-	48.6	61.3	46.4
External Evaluation Datasets											
JSRT	Int. & Ext.	197	-	50	Japan	Segm.	Organs	5	52.8	58.2	-
VinDr Rib	Int. & Ext.	196	-	49	Vietnam	Segm.	Ribs	20	-	-	-
BRAX	External	-	-	40.967	Brasil	Classif.	Findings	14	50.6	46.0	70.8

Table 1. **Overview of all the datasets used in this study.** They are grouped in 3 categories: pre-training of RayDINO, internal evaluation set (downstream adapter trained on them), or external evaluation set (adapter not trained on them). One dataset can be used in multiple setups. The number of exams used for the training, validation and test splits for each dataset is presented together with information about the country of origin, the type of benchmark and labels as well as the % of female and healthy exams and the average age.

P11 Table 2

CheXpert	Atelectasis, Cardiomegaly, Consolidation, Edema, Enlarged Cardiomediastinum, Fracture, Lung Lesion, Lung Opacity, No Finding, Pleural Effusion, Pleural Other, Pneumonia, Pneumothorax, Support Devices
MIMIC	Atelectasis, Cardiomegaly, Consolidation, Edema, Enlarged Cardiomediastinum, Fracture, Lung Lesion, Lung Opacity, No Finding, Pleural Effusion, Pleural Other, Pneumonia, Pneumothorax, Support Devices
NIH	Atelectasis, Cardiomegaly, Consolidation, Edema, Effusion, Emphysema, Fibrosis, Hernia, Infiltration, Mass, No Finding, Nodule, Pleural Thickening, Pneumonia, Pneumothorax
VinDr	Aortic Enlargement, Atelectasis, Calcification, Cardiomegaly, Clavicle Fracture, Consolidation, Copd, Edema, Emphysema, Enlarged Pa, Ild, Infiltration, Lung Cavity, Lung Cyst, Lung Opacity, Lung Tumor, Mediastinal Shift, No Finding, Nodule Mass, Other Disease, Other Lesion, Pleural Effusion, Pleural Thickening, Pneumonia, Pneumothorax, Pulmonary Fibrosis, Rib Fracture, Tuberculosis
PaxRAY++	Spine, Cervical Spine, Thoracic Spine, Lumbar Spine, Vertebrae C1, C2, C3, C4, C5, C6, C7, Vertebrae T1, T2, T3, T4, T5, T6, T7, T8, T9, T10, T11, T12, Vertebrae L1, L2, L3, L4, L5, Rib Cartilage, Sternum, Clavicles, Clavicle Left, Clavicle Right, Scapulas, Scapula Left, Scapula Right, Posterior Rib R1, R2, R3, R4, R5, R6, R7, R8, R9, R10, R11, R12, Anterior Rib R1, R2, R3, R4, R5, R6, R7, R8, R9, R10, R11, Posterior Rib L1, L2, L3, L4, L5, L6, L7, L8, L9, L10, L11, L12, Anterior Rib L1, L2, L3, L4, L5, L6, L7, L8, L9, L10, L11, Diaphragm, Left Hemidiaphragm, Right Hemidiaphragm, Stomach, Small Bowel, Duodenum, Liver, Pancreas, Kidney Left, Kidney Right, Cardiomediastinum, Upper Mediastinum, Lower Mediastinum, Anterior Mediastinum, Middle Mediastinum, Posterior Mediastinum, Heart, Heart Atrium Left, Heart Atrium Right, Heart Myocardium, Heart Ventricle Left, Heart Ventricle Right, Aorta, Ascending Aorta, Descending Aorta, Aortic Arch, Pulmonary Artery, Inferior Vena Cava, Esophagus, Lung, Right Lung, Left Lung, Lung Base, Mid Zone Lung, Upper Zone Lung, Apical Zone Lung, Right Upper Zone Lung, Right Mid Zone Lung, Right Lung Base, Right Apical Zone Lung, Left Upper Zone Lung, Left Mid Zone Lung, Left Lung Base, Left Apical Zone Lung, Lung Lower Lobe Left, Lung Upper Lobe Left, Lung Lower Lobe Right, Lung Middle Lobe Right, Lung Upper Lobe Right, Trachea, Tracheal Bifurcation, Breast, Breast Left, Breast Right
Scoliosis	MT Angle, PT Angle, TL-L Angle
Pneumothorax	Normal, Pneumothorax
POLCOVID	Covid, Normal, Pneumonia
JSRT	Left Lung, Right Lung, Left Clavicle, Right Clavicle, Heart
VinDr Rib	Rib R1, R2, R3, R4, R5, R6, R7, R8, R9, R10, L1, L2, L3, L4, L5, L6, L7, L8, L9, L10
BRAX	Atelectasis, Cardiomegaly, Consolidation, Edema, Enlarged Cardiomediastinum, Fracture, Lung Lesion, Lung Opacity, No Finding, Pleural Effusion, Pleural Other, Pneumonia, Pneumothorax, Support Devices
Catheter	Ett Abnormal, Ett Borderline, Ett Normal, Ngt Abnormal, Ngt Borderline, Ngt Normal, Cvc Abnormal, Cvc Borderline, Cvc Normal
MIMIC-LT	Adenopathy, Atelectasis, Azygos Lobe, Calcification Of The Aorta, Cardiomegaly, Clavicle Fracture, Consolidation, Edema, Emphysema, Enlarged Cardiomediastinum, Fibrosis, Fissure, Fracture, Granuloma, Hernia, Hydropneumothorax, Infarction, Infiltration, Kyphosis, Lobar Atelectasis, Lung Lesion, Lung Opacity, Mass, Nodule, Normal, Pleural Effusion, Pleural Other, Pleural Thickening, Pneumomediastinum, Pneumonia, Pneumoperitoneum, Pneumothorax, Pulmonary Embolism, Pulmonary Hypertension, Rib Fracture, Rounded Atelectasis, Subcutaneous Emphysema, Support Devices, Tortuous Aorta, Tuberculosis

Table 2. Overview of the different datasets and their detailed classes used in our study. The table presents a clear list of all the benchmarked X-Ray anomalies and anatomies, showing the extensive evaluation of RayDINO.

2. *‘providing separate AI models for each organ and anomaly visible in chest X-rays is impractical and can be unfeasible’. I completely agree on the infeasibility, but usually medical doctors are specialized on a specific domain of medicine that can involve a single organ. I would maybe remove ‘impractical’, stressing the fact that it involves a lot of manual work that does not scale with the amount of new medical data (as partially mentioned in the following sentence).*

Response

Thank you for the remark, we updated this sentence in the introduction and hope it now conveys better the message about the need for scalability for machine learning models.

P1 L18 Introduction: “However, providing separate AI models for each organ and anomaly visible in chest X-rays can be time consuming or even unfeasible since it requires a lot more manual work in scenarios where access to annotated data is difficult.”

3. *The introduction can be smoother, better linking some sentences (such as ‘Recent studies have raised concerns about the quality of automatic text-based data labeling and the reliability of AI systems trained on reports from medical images’, ‘). Recommend revising to improve readability for the less informed reader.*

Response

Thank you for the remark. We have revised the introduction section considerably to make it smoother and clearer with respect to the proposed model and the aim of our work. Moreover, a native English speaker has reviewed our manuscript to ensure clarity and correctness.

P1 L29-30 Introduction: “This is highlighted in recent studies, which have raised concerns about the quality of automated text-based data labeling and the reliability of AI systems trained on reports from medical images.”

4. *The paragraph in the introduction where RayDINO is described should be restructured. As presented, first, the experimental setup is described, then how the model is analyzed, then again, the experimental setup (the datasets, the benchmarks).*

Response

Following your remark, we have revised the presentation of our method considerably in the introduction. We included more details and made it more clear. In particular, we reorganized the presentation as follows. First, we introduce our model and the training scheme. Then, we describe the datasets and the evaluation protocol. Finally, we present the protocol for comparing our model to the state of the art. We hope that this way the reader could have a better overview of our study.

P1 L34-L35 Introduction: “We introduce RayDINO, the largest vision transformer (ViT) model of 307M parameters trained on a large dataset of 840k images compiled from four datasets of public X-ray images”

P2 L43 Introduction: "We evaluate its performance by using task-specific adapters on top of frozen RayDINO features (Fig. 1b,c) using eleven publicly available datasets from seven countries across four continents, both in internal and external settings. We report results from nine medical-imaging related tasks (Fig. 1d) divided into 21 benchmarks comprising more than 200 classes (Fig. 1e)."

P3 L52-59 Introduction: "Out of our nine tasks (Fig. 1d), the first three are evaluating the holistic understanding of our model: classification of X-ray findings and diseases, dense segmentation of diseases and organs, and generation of radiology reports (Fig. 2). The next three quantify the ability of RayDINO to generalize to new or rare labels: rare diseases and catheter position classification, unseen diseases classification and regression of scoliosis angle on unseen exam (Fig. 3). The final tasks focus on the clinical usability and potential bias of our foundation model: interpretability analysis, robustness between population in classification and segmentation, and sex and bias analysis (Fig. 4). We emphasize that, while the initial three tasks have previously been utilized in the evaluation of foundation models in radiology, our study is the first to develop and implement the six additional tasks."

P3 L64-70 Introduction: "Our findings indicate that RayDINO's representation provides state-of-the-art performance on all 21 benchmarks by surpassing the best available chest X-ray foundation models in the litterature on most of them (Fig. 1d), without relying on human annotations during its pre-training phase. In addition, RayDINO handles better rare categories, worst minority groups, and external demographics. Finally, as an additional source of information, our model also provides stronger interpretability, qualitatively validated by expert radiologists and quantitatively using attention pinpointing. Details about RayDINO along with baselines, data, metrics and experimental protocols are provided in the Online Methods and the Supplementary Materials sections."

5. *Results section – 1st paragraph - Elaborate further on how RayDINO was compared with benchmarks.*

Response

Initially, we included all these details in the Online Methods section of our manuscript. Following your comment, we have included more details in the first paragraph of the results section, elaborating on how RayDINO was adapted to the different benchmarks and tasks after the pretraining. Moreover, we included Table 3 providing an overview of the different compared benchmarks and task-specific adapters.

P3 L74-79 Results: "For each task, we trained and validated multiple task-specific adapters on top of the frozen-weights models using the exact same data and exhaustive hyperparameter grid search (as explained in the Experimental Protocols section of Methods) and then took the best performing model on the validation set. We replicated this five times for all models using different seeds and reported the average and 95% CI of the performance of the test set. To compare RayDINO with previous work, we reported

p-values of a t-test against the best performing baseline. . More details about our metrics are available in the Evaluation Metrics section of Online Methods.”

P12 305-318 Online Methods: “In all our evaluations, we compare our model to several publicly available X-ray foundation models. We select five recent methods, representative of the current state of the art, as comparison baselines. REFERS34 is a model trained using cross-supervision between radiology images and text from radiology reports. CheXzero35 employs contrastive language-image pretraining (akin to CLIP9) to produce a vision model able to tackle Chest X-ray images. KAD36 is similarly trained using paired radiology images and reports, with the addition of a knowledge-base component to improve performance. Inference is then performed following the CLIP zero-shot protocol, effectively performing classification over the list of extracted disease names. UNIChest37 consists of an improved model built on top of the KAD method. Finally, ARK638 is a foundation model trained on a concatenation of multiple existing datasets used in a classification training pipeline, effectively using a large amount of categorical supervision. Regarding training data, the models against which we show comparisons are trained on a relatively similar set of datasets as our model. REFERS, CheXzero, and KAD are trained on the MIMIC dataset16. UniChest additionally leverages NIH18, CheXpert17, and Vindr24. ARK6 uses all these datasets along with the data from the RSNA Pneumonia Detection Challenge 2 and the Shenzen dataset51. An overview of the different models used for the comparison of RayDINO together with information about the task-specific adapters used for each of the benchmarks is presented in Table 3.”

P13 Table 3

Model	Architecture	Num. Params	Pre-Train Datasets
Benchmarked Backbones			
UniChest[37]	ResNet50	55 M	CheXpert, MIMIC, NIH, VinDr
KAD[36]	ResNet50	55 M	MIMIC
ARK6[38]	Swin-B	91 M	CheXpert, MIMIC, NIH, VinDr, Pneumonia, Shenzhen
CheXzero[35]	ViT-B	86 M	MIMIC
Refers[34]	ViT-B	86 M	MIMIC
RayDINO (ours)	ViT-L	307 M	CheXpert, MIMIC, NIH, PadChest
Task-Specific Adapters			
Classification / Regression Adapter	2 layers MLP + ReLU	0.1-1 M	-
Segmentation Adapter	UperNet[52]	49 M	-
Text Adapter	LLM (Llama[53])	7 B	-

Table 3. **Parameter size** of the different backbones we use in this study along with the task-specific adapters.

6. <u>*‘Furthermore, despite using 100x less supervised parameters’ 100x less supervised parameters compared to what?*</u>

Response

Thank you for the remark. Thanks to the unsupervised pretraining, RayDINO generates holistic and powerful representations of chest X-Rays that are easily adaptable for various tasks and problems.

Since we keep the representations frozen (no full finetuning of the large network), only very light task-specific adapters need to be trained for each problem. This is opposed to fully supervised methods where task-specific representations need to be obtained (fully finetuned for a specific task, making them less general). We agree that the sentence was not very clear, so we rephrased, with we added also Table 3 to the online methods presenting all the parameters for the benchmarked models and the details of the task-specific adapters

P3 L84 Results: "Furthermore, despite using 100 x less supervised parameters than other compared models (Table 3), RayDINO remains competitive on CheXpert"

P13 Table 3

7. *How can RayDINO be multimodal? In Figure 1 it looks like it has been trained just with images.*

The report generation is not clear to me. The text states that only images are used during the training, therefore it sounds strange that the feature space of images is perfectly aligned with the one of reports, so that image features can feed a LLM without any other modification. Can you please better describe the report generation?

Response

We are sorry for the confusion. RayDINO is a vision transformer trained on a dataset of X-ray images only. As mentioned in your question and indicated in Figure 1, it has been trained only with images, following the DINOv2 pretraining strategy. However, we can evaluate image-only models on the task of report generation by using their representations as inputs to an adapter to a pretrained Large Language Model (LLM).

LLMs use text tokens (chunks of words) as input. Raw text is first "tokenized" and then tokens are converted into embeddings (vectors) using a lookup table. Those vectors go through a transformer, and at the last layer, a probability distribution for the next token is computed. In our setup, we mix regular tokens with vision tokens from RayDINO as embeddings. RayDINO analyzes the image, and the vectors produced are used as "prompts" for the LLM to generate the report. This is a standard procedure for Vision Language Models (VLMs) like LLaVa (*Visual Instruction Tuning*, Haotian Liu et al. 2023), BLIP (Junnan Li et al. 2022), etc.

As you noticed, RayDINO embeddings are not directly aligned with the LLM, so we have to finetune the LLM, exactly like we did with all other task-specific adapters. In that specific evaluation, the adapter is much larger. We used reports from the MIMIC dataset and split them into train/val/test sets following MAIRA-1 (Hyland et al., 2023). By doing so, we showcase the fact that despite being an image-only model, RayDINO can be easily adapted inside bigger architectures to exploit multi-modal datasets and solve multi-modal tasks.

P1 L34-L35 Introduction: "We introduce RayDINO, the largest vision transformer (ViT) model of 307M parameters trained on a large dataset of 840k images compiled from four datasets of public X-ray images"

P13 L359-364 Online Methods: "We finetune a publicly available large language model, namely the LLama2-7B model to perform report generation. We first convert image features,

using a small two-layer Transformer adapter, into tokens of the dimensionality expected by the language model. Then, this language model can be trained to produce reports directly from the image tokens. To this end, we employ an image captioning loss to generate the findings section of the MIMIC reports, using the indication section as prompt when available. We train for 10.000 iterations with a batch size of 8 sequences of 2048 tokens. For a fair comparison, we align on the split and the text preprocessing used in MAIRA-1, and refer to their manuscript for more details."

P19L541-547 Supplementary materials: "We present in Table S1 the results of the evaluation on the generation of reports for all the competing models considered, including the strong recent state-of-the-art MAIRA-1 model, specifically designed for this task; we provide 95% confidence intervals on performance metrics for better comparison. We observe that RayDINO performs on par or signifiantly better than all competitor models on all metrics. In particular, RayDINO significantly outperforms MAIRA-1 on radiologist-aligned metrics (RadGraph F1, CheXbert score, RadCliQ). We note that, interestingly, RayDINO performs on par with the strongly-engineered GPT4-augmented version MAIRA-1 in all metrics, while relying only on the MIMIC-CXR reports."

8. *The comparison is sometimes hard to understand. Can you please report your result, the benchmark result and the difference in performance, instead of your result and the difference in performance only?*

   The results notation is sometimes fuzzy, especially when the improvement is limited (e.g. +0.9 over UniChest on rare diseases). Could you report the performance metrics between 0 and 1?

   Response

   We are sorry for the bad notation. We updated the Results section and the figures to put every metric between 0 and 1 and to facilitate the comparison with other models. Moreover, as you suggested, we added the benchmark result together with the performance of our model and the comparison for better understanding. In particular, for each comparison, we present the average and 95% CI of the performance of RayDINO in the test set, followed by the the difference and absolute value of the best-performing benchmarked model on the same test set and the p-values of a t-test between these two methods.

   P3-7 Results section, and Figure 1, 2, 3 and 4.

   Example: P3 L82-84 Results: " Fig. 2a indicates that RayDINO significantly outperforms the best alternatives on the MIMIC dataset with .817 AUROC (95%CI [.816 -.818], +.010 above .807 of UniChest, p ≤ 1e−5) and the NIH dataset with .838 AUROC (95%CI [.837 -.838], +.019 above .819 of ARK6, p ≤ 1e−5)."

9. *The division between age groups (Section Examining Performance Across Sex and Age Groups) can be misleading: a kid shows a completely different chest morphology compared with a person of 20 years old, while instead a 35-year-old person is more similar to 50 years old. Would it be possible to repeat the experiment, using 0-12, 12-35, 35-60, 60-100 as groups?*

   Response

Thank you for your insightful suggestion regarding the age group divisions. After further analysis, we would like to clarify that the MIMIC dataset does not contain images from children under 15 years old. To address your question we updated the corresponding sentences to emphasize the age range for the young group. However, exploring whether the model can generalize to younger children is a valuable follow-up for future work.

P7 L185 Results: " We divided the MIMIC dataset into three age groups: Young/Y (15-35 years), Middle/M (35-60 years), and Elder/E (60-100 years)"

10. *'Explainability. I would change the title to 'Interpretability' since Explainability is a slightly different domain (focuses on how a model produces results to justify decisions) and what you are proposing exploits interpretability (investigates the algorithm logic, helping to make algorithm components comprehensible to humans). Furthermore, interpretability and explainability are both used in the text. I would just use interpretability.*

Response

Thank you for the comment, we agree that our analysis focuses more on the interpretability of the model rather than its explainability. We updated all related sentences and figures with interpretability instead of explainability to make it more clear to the reader.

All occurrences of "explainability" (including in Figure 1,2,3 and 4) have been modified to "interpretability"

11. *Data section within methods is a bit light. I think this section should be restructured, maybe adding a table showing data distribution and better describing their characteristics (machine used to acquired data, origin) to highlight the heterogeneity and make your paper stronger. Also on the downstream task, it would be nice to see how data are used and to better highlight how the classifier is finetuned (I guess just training the layers after the image encoder).*

Response

We thank you for pointing out this issue. We improved the data description section in the Online Methods section to explain our data processing. In particular, we have added Figure 5 showcasing the different distributions for each dataset with respect to their intensity values to highlight the differences between the used datasets in our study. Indeed, one can observe that the distribution of the intensity values for the pretraining (MIMIC, CheXpert, NIH, and PadChest) and evaluated datasets (VinDr, PAXRay++, Sclerosis, Pneumothorax, PolCovid, JSRT, VinDr Rib, and BRAX) are very diverse highlighting the power of RayDino with respect to holistic and generalizable representations. To highlight and clarify the information about the datasets used, we included also two additional tables. More specifically, Table 1 summarises the different statistics of the datasets used, including the training, validation, and test splits, the type of problem and the labels that they are dealing with, the countries of origin, as well as the sex and age information. Once more, this table highlights the diversity and complementarity of the datasets used in this study. Finally, Table 2 highlights the class labels for each dataset and used as endpoints for our model.

With respect to your question about the downstream tasks, your intuition is correct. Small, light weighted adapters are used after our pre-trained image encoder to adapt to the different types of

problems as well as labels available to each dataset. To make this point more clear for the reader, we have updated Figure 1 on the main paper separating the pre-training (Fig. 1A), evaluation (Fig. 1B), and metric computation (Fig. 1C) protocols, and we added Table 3, including the number of parameters for each task-specific adapter. Moreover, we have re-organized the introduction section, including more information about the adaptation of RayDINO in the different tasks used in this manuscript.

P10 L296-301 Online Methods: "For all the dataset, we applied the same preprocessing including:

• In case of raw dicom files, we use a Look-Up Table (LUT) to convert pixels to the [0 − 255] range using a window width of (max − min) and a window level of (max + min)/2.

• All exams were resized to 512x512 pixels and transformed to three channels by replicating the channel provided.

• All exams were normalized using classical ImageNet parameters of mean = (0.485, 0.456, 0.406) and std = (0.229, 0.224, 0.225)"

P10 Table 1

P11 Table 2

P12 Figure 5

P13 Table 3

12. *<u>How are the data used to pre-train the model? How are they split in training, validation, and testing later?</u>*

Response

Since we used publicly available datasets for this study, we maintained the splits provided for the training, validation, and testing of the models to maintain the proper comparison with other methods and make our results reproducible. When no such official splits are available, we used respectively 80%, 10% and 10% of the data for the train, validation and test sets. More specifically, we pre-trained RayDINO using the training sets of CheXpert, MIMIC, NIH-14 and PadChest. We also ensured that each patient was in one set only, as they can be associated with multiple X-rays on different datasets. To make this point more clear in the manuscript, we have added Table 1, indicating the way that we used each dataset.

P10 L293-296 "All datasets, together with their repartition to pretraining of RayDINO, their distributions, and their available annotations, are summarized in Table 1 and 2. We use train, validation, and test splits for all evaluations. To make our results reproducible we used all official test splits when available. When no such official splits are available, we used respectively 80%, 10%, and 10% of the data for the train, validation, and test sets."

P10 Table 1

Name	Used For	Train	Val	Test	Country	Type	Labels	# Label	% Female	Avg. Age	% Healthy
Pretraining Datasets											
PadChest	Pretrain	160.845	-	-	Spain	-	-	-	49.6	58.6	31.4
CheXpert	Pre. & Int.	223.414	234	668	USA	Classif.	Findings	14	40.7	60.6	10.0
MIMIC	Pre. & Int.	368.960	2.991	5.159	USA	Classif.	Findings	14	47.5	61.6	38.3
NIH	Pre. & Int.	83.524	3.000	25.596	USA	Classif.	Findings	15	44.0	46.6	58.4
Internal Evaluation Datasets											
VinDr	Internal	14.000	1.000	3.000	Vietnam	Classif.	Findings	28	-	-	70.7
PAXRay++	Internal	11.802	1.475	1.476	Multiple	Segm.	Bones & Organs	159	-	-	-
Scoliosis	Internal	481	-	128	Canada	Reg.	Spine angles	3	-	-	-
Pneumothorax	Internal	10.675	1.372	3.205	USA	Segm.	Diseases	2	-	-	77.7
POLCOVID	Internal	2.985	509	1.315	Poland	Classif.	Diseases	3	47.4	60.2	65.8
Catheter	Internal	24.066	3.008	3.009	USA	Classif.	Catheter Pos.	9	-	-	-
MIMIC-LongTail	Internal	264.849	36.769	75.492	USA	Classif.	Findings	40	47.5	61.6	38.3
Report Generation	Internal	267.215	2.114	3.822	USA	Text Gen.	Radiological Reports	-	48.6	61.3	46.4
External Evaluation Datasets											
JSRT	Int. & Ext.	197	-	50	Japan	Segm.	Organs	5	52.8	58.2	-
VinDr Rib	Int. & Ext.	196	-	49	Vietnam	Segm.	Ribs	20	-	-	-
BRAX	External	-	-	40.967	Brasil	Classif.	Findings	14	50.6	46.0	70.8

Table 1. Overview of all the datasets used in this study. They are grouped in 3 categories: pre-training of RayDINO, internal evaluation set (downstream adapter trained on them), or external evaluation set (adapter not trained on them). One dataset can be used in multiple setups. The number of exams used for the training, validation and test splits for each dataset is presented together with information about the country of origin, the type of benchmark and labels as well as the % of female and healthy exams and the average age.

P11 Table 2

CheXpert	Atelectasis, Cardiomegaly, Consolidation, Edema, Enlarged Cardiomediastinum, Fracture, Lung Lesion, Lung Opacity, No Finding, Pleural Effusion, Pleural Other, Pneumonia, Pneumothorax, Support Devices
MIMIC	Atelectasis, Cardiomegaly, Consolidation, Edema, Enlarged Cardiomediastinum, Fracture, Lung Lesion, Lung Opacity, No Finding, Pleural Effusion, Pleural Other, Pneumonia, Pneumothorax, Support Devices
NIH	Atelectasis, Cardiomegaly, Consolidation, Edema, Effusion, Emphysema, Fibrosis, Hernia, Infiltration, Mass, No Finding, Nodule, Pleural Thickening, Pneumonia, Pneumothorax
VinDr	Aortic Enlargement, Atelectasis, Calcification, Cardiomegaly, Clavicle Fracture, Consolidation, Copd, Edema, Emphysema, Enlarged Pa, Ild, Infiltration, Lung Cavity, Lung Cyst, Lung Opacity, Lung Tumor, Mediastinal Shift, No Finding, Nodule Mass, Other Disease, Other Lesion, Pleural Effusion, Pleural Thickening, Pneumonia, Pneumothorax, Pulmonary Fibrosis, Rib Fracture, Tuberculosis
PaxRAY++	Spine, Cervical Spine, Thoracic Spine, Lumbar Spine, Vertebrae C1, C2, C3, C4, C5, C6, C7, Vertebrae T1, T2, T3, T4, T5, T6, T7, T8, T9, T10, T11, T12, Vertebrae L1, L2, L3, L4, L5, Rib Cartilage, Sternum, Clavicles, Clavicle Left, Clavicle Right, Scapulas, Scapula Left, Scapula Right, Posterior Rib R1, R2, R3, R4, R5, R6, R7, R8, R9, R10, R11, R12, Anterior Rib R1, R2, R3, R4, R5, R6, R7, R8, R9, R10, R11, Posterior Rib L1, L2, L3, L4, L5, L6, L7, L8, L9, L10, L11, L12, Anterior Rib L1, L2, L3, L4, L5, L6, L7, L8, L9, L10, L11, Diaphragm, Left Hemidiaphragm, Right Hemidiaphragm, Stomach, Small Bowel, Duodenum, Liver, Pancreas, Kidney Left, Kidney Right, Cardiomediastinum, Upper Mediastinum, Lower Mediastinum, Anterior Mediastinum, Middle Mediastinum, Posterior Mediastinum, Heart, Heart Atrium Left, Heart Atrium Right, Heart Myocardium, Heart Ventricle Left, Heart Ventricle Right, Aorta, Ascending Aorta, Descending Aorta, Aortic Arch, Pulmonary Artery, Inferior Vena Cava, Esophagus, Lung, Right Lung, Left Lung, Lung Base, Mid Zone Lung, Upper Zone Lung, Apical Zone Lung, Right Upper Zone Lung, Right Mid Zone Lung, Right Lung Base, Right Apical Zone Lung, Left Upper Zone Lung, Left Mid Zone Lung, Left Lung Base, Left Apical Zone Lung, Lung Lower Lobe Left, Lung Upper Lobe Left, Lung Lower Lobe Right, Lung Middle Lobe Right, Lung Upper Lobe Right, Trachea, Tracheal Bifurcation, Breast, Breast Left, Breast Right
Scoliosis	MT Angle, PT Angle, TL-L Angle
Pneumothorax	Normal, Pneumothorax
POLCOVID	Covid, Normal, Pneumonia
JSRT	Left Lung, Right Lung, Left Clavicle, Right Clavicle, Heart
VinDr Rib	Rib R1, R2, R3, R4, R5, R6, R7, R8, R9, R10, L1, L2, L3, L4, L5, L6, L7, L8, L9, L10
BRAX	Atelectasis, Cardiomegaly, Consolidation, Edema, Enlarged Cardiomediastinum, Fracture, Lung Lesion, Lung Opacity, No Finding, Pleural Effusion, Pleural Other, Pneumonia, Pneumothorax, Support Devices
Catheter	Ett Abnormal, Ett Borderline, Ett Normal, Ngt Abnormal, Ngt Borderline, Ngt Normal, Cvc Abnormal, Cvc Borderline, Cvc Normal
MIMIC-LT	Adenopathy, Atelectasis, Azygos Lobe, Calcification Of The Aorta, Cardiomegaly, Clavicle Fracture, Consolidation, Edema, Emphysema, Enlarged Cardiomediastinum, Fibrosis, Fissure, Fracture, Granuloma, Hernia, Hydropneumothorax, Infarction, Infiltration, Kyphosis, Lobar Atelectasis, Lung Lesion, Lung Opacity, Mass, Nodule, Normal, Pleural Effusion, Pleural Other, Pleural Thickening, Pneumomediastinum, Pneumonia, Pneumoperitoneum, Pneumothorax, Pulmonary Embolism, Pulmonary Hypertension, Rib Fracture, Rounded Atelectasis, Subcutaneous Emphysema, Support Devices, Tortuous Aorta, Tuberculosis

Table 2. Overview of the different datasets and their detailed classes used in our study. The table presents a clear list of all the benchmarked X-Ray anomalies and anatomies, showing the extensive evaluation of RayDINO.

13. *I have a doubt about the result: a single value is reported for every task analyze. Did you train a single model? Are you reporting the best model among multiple models? This detail must be clarified. I can imagine you trained just a single backbone with SSL, due to the massive amount of data. What about the downstream tasks?*

    I have some doubts about the comparisons: how did you compare the benchmark algorithms? Did you use the same data to pre-train and fine-tune? Can you please better clarify this detail, since the paper is based on this comparison, but it is not clear to me.

    Response

    Thank you very much for the remark, and sorry for the confusion. Indeed, our pre-trained RayDINO model has been trained only once using the complete set of pretraining data and the DINOv2 self-supervised method. As indicated in our manuscript on the unsupervised pretraining section, the training of RayDINO is performed on 128 A100-80GB Nvidia GPUs and lasts 36 hours. Given the model's size and the number of images in the training dataset, training multiple models would require a lot of computational resources and time, while it will not affect the self-supervised way of pretraining the model.
    For the benchmarked algorithms, we used the state of the art models for each X-Ray task we were comparing. All the details about the benchmarked models are included in the Methods and the section Comparisons & Baselines, indicating the dataset and method that each of these models had been trained. Indeed, we did not retrain the benchmark algorithms and instead used the provided open-sourced models by the authors. The main bottleneck of those algorithms is that they require labels and thus can't be pre-trained on datasets with mismatched annotations. On the other hand, thanks to self-supervised learning, RayDINO can be pretrained on every concatenation of datasets, even with different or no annotations. This way, RayDINO and the benchmarked models are used as-is by freezing their weights.
    For the different downstream tasks, we trained and validated multiple task-specific adapters, using annotations, on top of the frozen-weights models using the exact same data and exhaustive hyperparameter grid search (as explained in the Experimental Protocols section of Methods). We selected the best-performing model on the validation set for each benchmarked model. We then replicated this setup five times for all models using different seeds and reported the average and 95% CI of the performance of the test set. To make this point clear we have updated Figure 1 with Fig.1C, indicating the way that the metrics had been computed. Moreover, we have updated the Results section, adding more explanations about the adapters. Please note that we kept the same protocol for all the benchmarked models as well. This means that we train tens or hundreds of adapters for each benchmarked model, take the best of them on the validation sets, and then report their performances on the test splits. While compute-intensive, this setup is fairer for the other algorithms, as we optimized hyperparameters for all models and not just for RayDINO. This is, however, not the standard practice for other scientific articles, where a single adapter without tuning hyperparameters, especially for past methods used as a baseline (Zhou et al. 2022, Zhang et al. 2023, Ma et al. 2023, Azizi et al. 2023, Seibold et al. 2023).

    P2 L43-44 Introduction: "We evaluate RayDINO's performance by using task-specific adapters on top of the obtained frozen features (Fig. 1b,c) using eleven publicly available datasets from seven countries across four continents, both in internal and external settings"

P2 Figure 1c

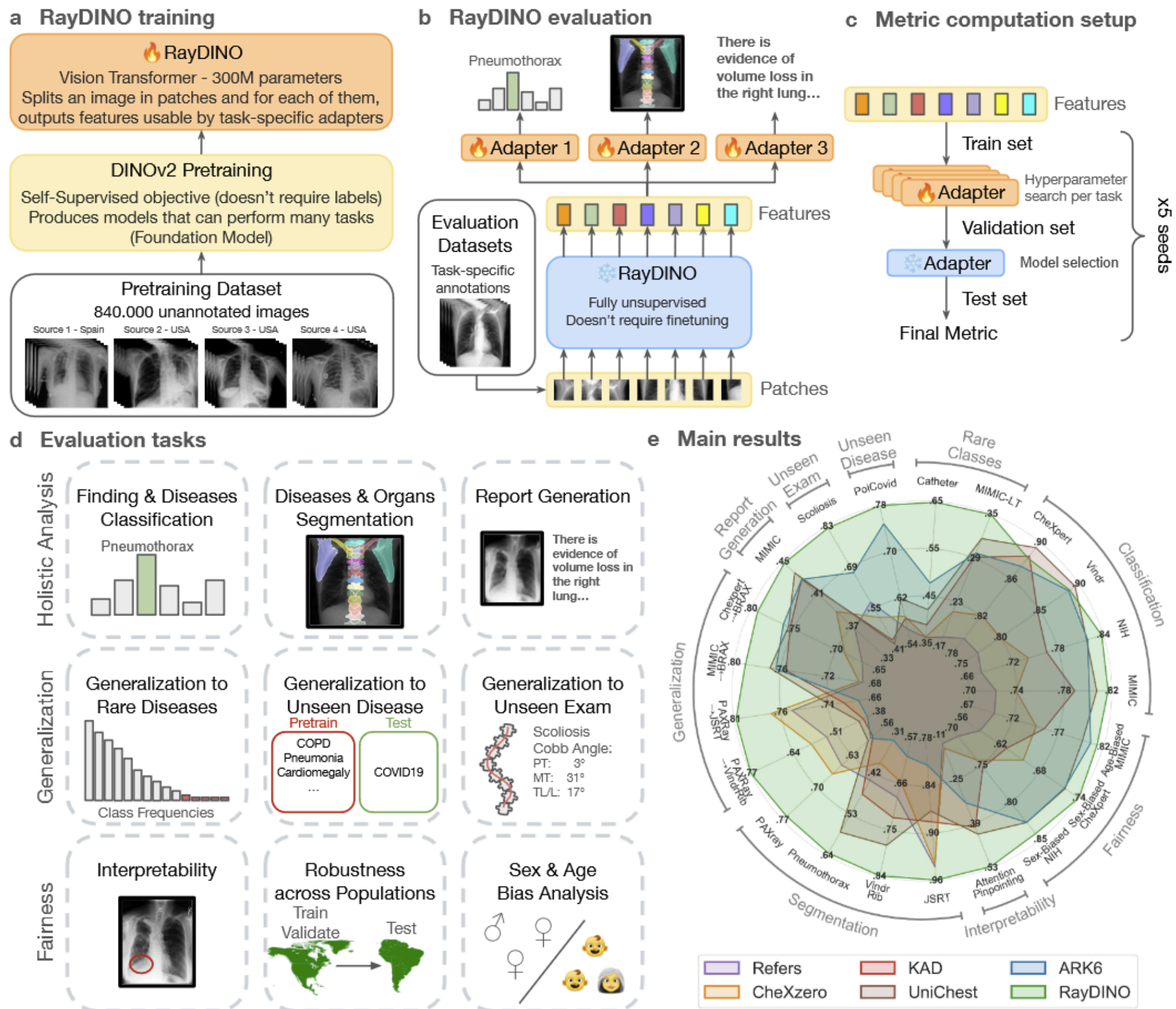

Figure 1. We introduce RayDINO, a foundation model for holistic and fairer analysis of chest X-rays. a) RayDINO is a vision transformer trained using DINOv2 objectives on four different datasets from the USA and Europe. **b)** We evaluate RayDINO and other models as-is on all tasks without any modification or specialization of their parameters by using very small task-specific adapters. **c)** Our supervised adapters are trained and validated for each task using a hyperparameters search. We repeated this setup 5 times to produce confidence intervals and compare models. **d)** RayDINO is evaluated on tasks divided into three categories. **e)** RayDINO consistently delivers excellent performance on 21 benchmarks (AUROC for Classification and Fairness, macro Accuracy for interpretability, mDice for Segmentation, AUROC for BRAX Generalization, and mDice for PAXRay Generalization, CheXbert vector similarity for Report Generation, Pearson's correlation for Unseen Exam, macro Accuracy for Unseen Disease, AUPRC/Area Under Precision Recall Curve for Rare Classes).

P3 L74-79 Results: " For each task, we trained and validated multiple task-specific adapters on top of the frozen-weights models using the exact same data and exhaustive hyperparameter grid search (as explained in the Experimental Protocols section of Online Methods) and then took the best-performing model on the validation set. We replicated this five times for all models using different seeds and reported the average and 95% confidence interval (CI) of the performance of the test set. To compare RayDINO with previous work, we reported p-values of a t-test against the best-performing baseline. More details about our metrics are available in the Evaluation Metrics section of Online Methods."

REVIEWERS #3 and #4

1. *#1) Thanks for this impressive and fascinating manuscript. I will break comments in to larger issues and smaller issues. Just to be clear, I am a clinician scientist who continues to practice medicine, yet have a reasonable understanding of AI methods and techniques and have used them for my own research.*

   The first issue is that the manuscript is overflowing with methods, technics and results, but actually far too many to be able to sufficiently explain what was done and how to interpret them. This looks more like a compressed thesis than a research manuscript. In the effort to squeeze this all into a manuscript with a limited number of words, much of the detail needed for the readers to trust and understand the tool are missing. More explicit examples of the are as follows;

   Response

   We are sorry for the complexity of our original submission, and thank you for highlighting the range and extent of our study. In this manuscript, we present RayDINO, the state-of-the-art foundation model for X-Ray data, evaluated on a large diversity of tasks and problems for radiological analysis. This extensive evaluation highlights the holistic and generalizable power of our model and opens up very important perspectives for research and clinical use. We believe that foundation models, such as RayDINO, need to be evaluated on diverse and challenging tasks to indicate their impact and importance, challenging their limits. To make the manuscript easier to follow, we have made substantial changes, following your and Reviewer 1 and 2 comments, to the different sections, and we have included more information about the different details of the methods and results. We hope these changes will address your comments and highlight the impact of our work on the community.

2. *Unclear Radiology Tasks: The authors mention nine radiology tasks, but they fail to clarify what these tasks entail. They categorize them into three broad categories: classification, segmentation, and report generation, but specific details about each of the nine tasks are missing. For example, in the classification tasks, it is not clear what exactly is being classified.*

   Response

   Thank you for your insightful comment, and we are sorry for not being more specific about the tasks we were using. The datasets used in our study are publicly available and commonly used by the community for benchmarking models on different radiology tasks. In the manuscript, we clearly cite the original manuscripts of the datasets that provide all the information needed for their composition. For simplicity and better presentation of our results, we indeed grouped all the datasets with respect to the different tasks they tackle to: classification, segmentation, and report generation as the main tasks that foundation models are evaluated, and we also provided analysis for rare and under representative classes, out of domain performance and robustness of the model with respect to demographic, sex and age characteristics. However, we agree with your comment that a better overview of the datasets used would provide a more complete understanding of our evaluation setting. For this reason, we added a better description of the datasets in the introduction section. Moreover, in the data section of the online methods, we provided two additional tables (Table 1 and 2) summarizing the statistics and tasks for each dataset. We hope that this new version highlights

the scale of the experiments better and explains the different classification and segmentation tasks per dataset in a more complete way.

P3 L52-59 Introduction: "Out of our nine tasks (Fig. 1d), the first three are evaluating the holistic understanding of our model: classification of X-ray findings and diseases, dense segmentation of diseases and organs, and generation of radiology reports (Fig. 2). The next three quantify the ability of RayDINO to generalize to new or rare labels: rare diseases and catheter position classification, unseen diseases classification and regression of scoliosis angle on unseen exam (Fig. 3). The final tasks focus on the clinical usability and potential bias of our foundation model: interpretability analysis, robustness between population in classification and segmentation, and sex and bias analysis (Fig. 4). We emphasize that, while the initial three tasks have previously been utilized in the evaluation of foundation models in radiology, our study is the first to develop and implement the six additional tasks."

P10 Table 1

P11 Table 2

3. *Missing Data Processing Explanation: There's no mention of how the authors process their data, which is a crucial part of any machine learning pipeline. Data preprocessing can heavily influence the results, and without this information, it's hard to replicate or trust their findings.*

Response

We thank you for the comment. We improved the data section in the Online Methods to explain our data processing. In particular, we mention the different preprocessing steps for the harmonizing of the XRay imaging for all the datasets. We also added Figure 5, showcasing the different distribution of the intensity values for each dataset to highlight the heterogeneity of these datasets. Moreover, in Table 1 all the information about the training, validation, and testing splits are provided. Since we used publicly available datasets for this study, we maintained the splits provided for the training, validation, and test of the models to maintain the proper comparison with other methods and make our results reproducible. When no such official splits were available, we used respectively 80%, 10%, and 10% of the data for the train, validation, and test sets. We use the train set as a validation set for JSRT and CinDr Rib given their official splits. We hope that these additions, together with the open availability of our model and code, will make our study reproducible.

P10 L296-301 Online Methods: " For all the dataset, we applied the same preprocessing including:

• In case of raw dicom files, we use a Look-Up Table (LUT) to convert pixels to the [0 − 255] range using a window width of (max − min) and a window level of (max + min)/2.299

• All exams were resized to 512x512 pixels and transformed to three channels by replicating the channel provided.

• All exams were normalized using classical ImageNet parameters of mean = (0.485, 0.456, 0.406) and std = (0.229, 0.224, 0.225)."

P10 Table 1

P12 Figure 5

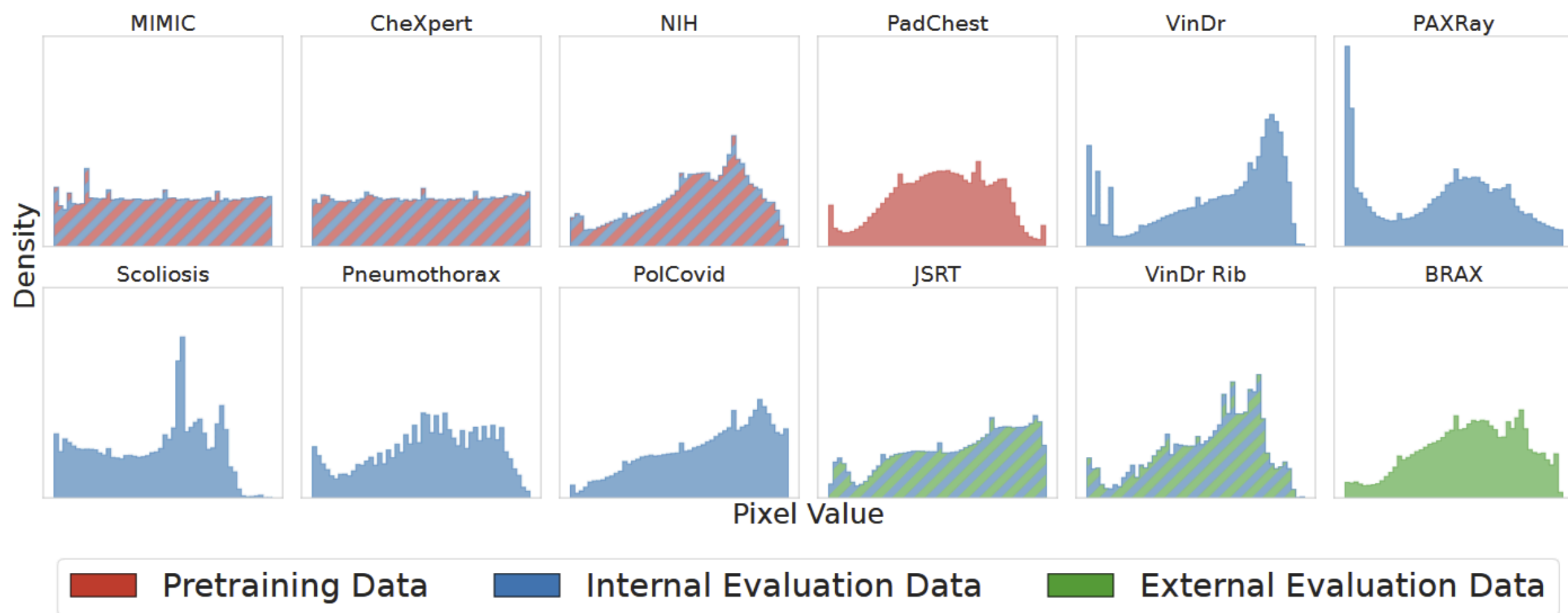

Figure 5. **Non-zero pixel values** histogram for each dataset in this study. For each histogram we indicate with different colors their use to pretraining, internal and external evaluation datasets.

4. *Lack of Clear Presentation Despite Substantial Effort: While the paper reflects considerable work, the presentation is insufficient to convey the depth of their efforts. The authors introduce various techniques and tasks but don't explain them in detail, making it hard for readers to follow along. If the authors broke down their methodology and results more thoroughly, it would significantly improve the clarity and perceived quality of their work.*

Response

We are sorry for the lack of a clear presentation of our original manuscript. Following your comments and the comments of reviewers 1 & 2 we made substantial revisions to make the presentation of our manuscript clearer. In particular, the introduction section has been reorganized to give a better overview of the motivation, methodology, and evaluation of our RayDINO model. Moreover, we enhanced the results section and Figure 1 to give more details about the downstream tasks we were benchmarking. In addition, more details have been added to the dataset section of the online methods and supplementary material to enhance the implementation details and make our work reproducible. We hope that with these changes, the impact and scale of our work are highlighted while making it easy to follow.

All the introduction, and beginning of the Results sections.

Examples:

P2 L43-L46 Introduction: "We evaluate RayDINO's performance by using task-specific adapters on top of the obtained frozen features (Fig. 1b,c) using eleven publicly available datasets from seven countries across four continents, both in internal and external settings. We report an extensive evaluation on nine medical-imaging related tasks (Fig. 1d) divided into 21 benchmarks comprising more than 200 classes (Fig. 1e)"

P2 L52-59 Introduction: "Out of our nine tasks (Fig. 1d), the first three are evaluating the holistic understanding of our model: classification of X-ray findings and diseases, dense segmentation of diseases and organs, and generation of radiology reports (Fig. 2). The next three quantify the ability of RayDINO to generalize to new or rare labels: rare diseases and catheter position classification, unseen diseases classification and regression of scoliosis angle on unseen exam (Fig. 3). The final tasks focus on the clinical usability and potential bias of our foundation model: interpretability analysis, robustness between population in classification and segmentation, and sex and bias analysis (Fig. 4). We emphasize that, while the initial three tasks have previously been utilized in the evaluation of foundation models in radiology, our study is the first to develop and implement the six additional tasks."

P3 L74-79 Results: "For each task, we trained and validated multiple task-specific adapters on top of the frozen-weights models using the exact same data and exhaustive hyperparameter grid search (as explained in the Experimental Protocols section of Methods) and then took the best performing model on the validation set. We replicated this five times for all models using different seeds and reported the average and 95% CI of the performance of the test set. To compare RayDINO with previous work, we reported p-values of a t-test against the best performing baseline. . More details about our metrics are available in the Evaluation Metrics section of Online Methods."

P2 Figure 1

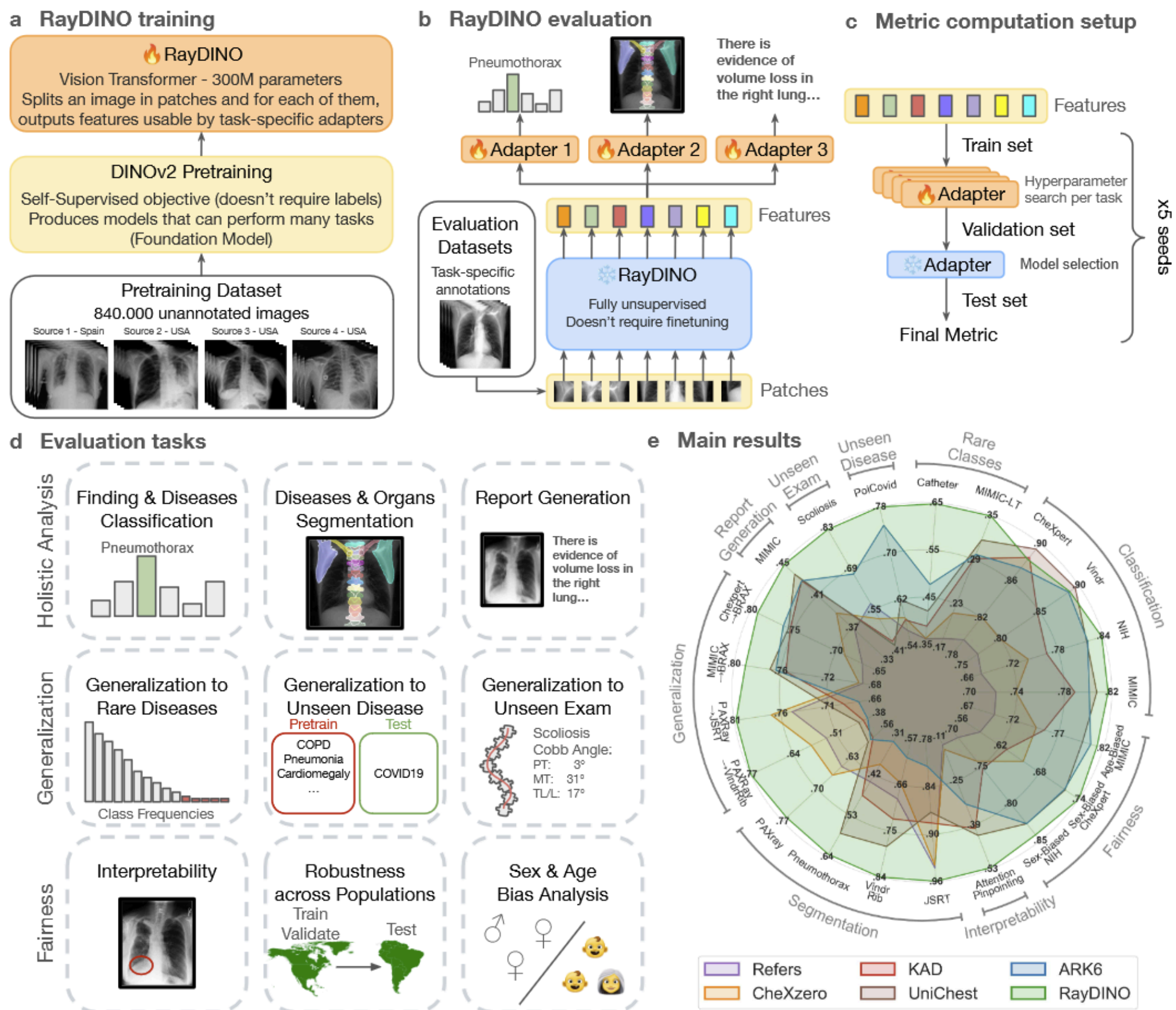

Figure 1. We introduce RayDINO, a foundation model for holistic and fairer analysis of chest X-rays. a) RayDINO is a vision transformer trained using DINOv2 objectives on four different datasets from the USA and Europe. **b)** We evaluate RayDINO and other models as-is on all tasks without any modification or specialization of their parameters by using very small task-specific adapters. **c)** Our supervised adapters are trained and validated for each task using a hyperparameters search. We repeated this setup 5 times to produce confidence intervals and compare models. **d)** RayDINO is evaluated on tasks divided into three categories. **e)** RayDINO consistently delivers excellent performance on 21 benchmarks (AUROC for Classification and Fairness, macro Accuracy for interpretability, mDice for Segmentation, AUROC for BRAX Generalization, and mDice for PAXRay Generalization, CheXbert vector similarity for Report Generation, Pearson's correlation for Unseen Exam, macro Accuracy for Unseen Disease, AUPRC/Area Under Precision Recall Curve for Rare Classes).

5. <u>The authors frequently use terms like "foundation models" and "self-supervised learning" but fail to provide in-depth explanations or examples of how these concepts are applied in their specific work. It would be a very small group of readers that really fully understand all the terms used. One of the reasons for the minimal explanation is the massive amount of work that is being introduced, so it is clear that better explanations would require a significant amount of words/space.</u>

Response

We are sorry for the lack of details about these technical terms. We agree that in our previous version, the self-supervised learning part of our RayDINO foundation model was not clearly highlighted. We reorganized the introduction section to introduce the foundation model and the self-supervised pretraining more clearly, while we updated also Figure 1 to illustrate these terms. We

tried this new version to give a better overview of the different downstream tasks and datasets to make our contributions easier to follow.

P1 L21-25 "Foundation models offer a solution to these challenges. They are large-scale, pre-trained models that produce generalizable and powerful representations of data, useful for a wide range of more specific downstream tasks. In contrast to supervised learning, they are trained in a more general manner using large amounts of data, thus capturing task-agnostic characteristics, and leading to more holistic, patient-centered, and unbiased representations."

P2 Figure 1a

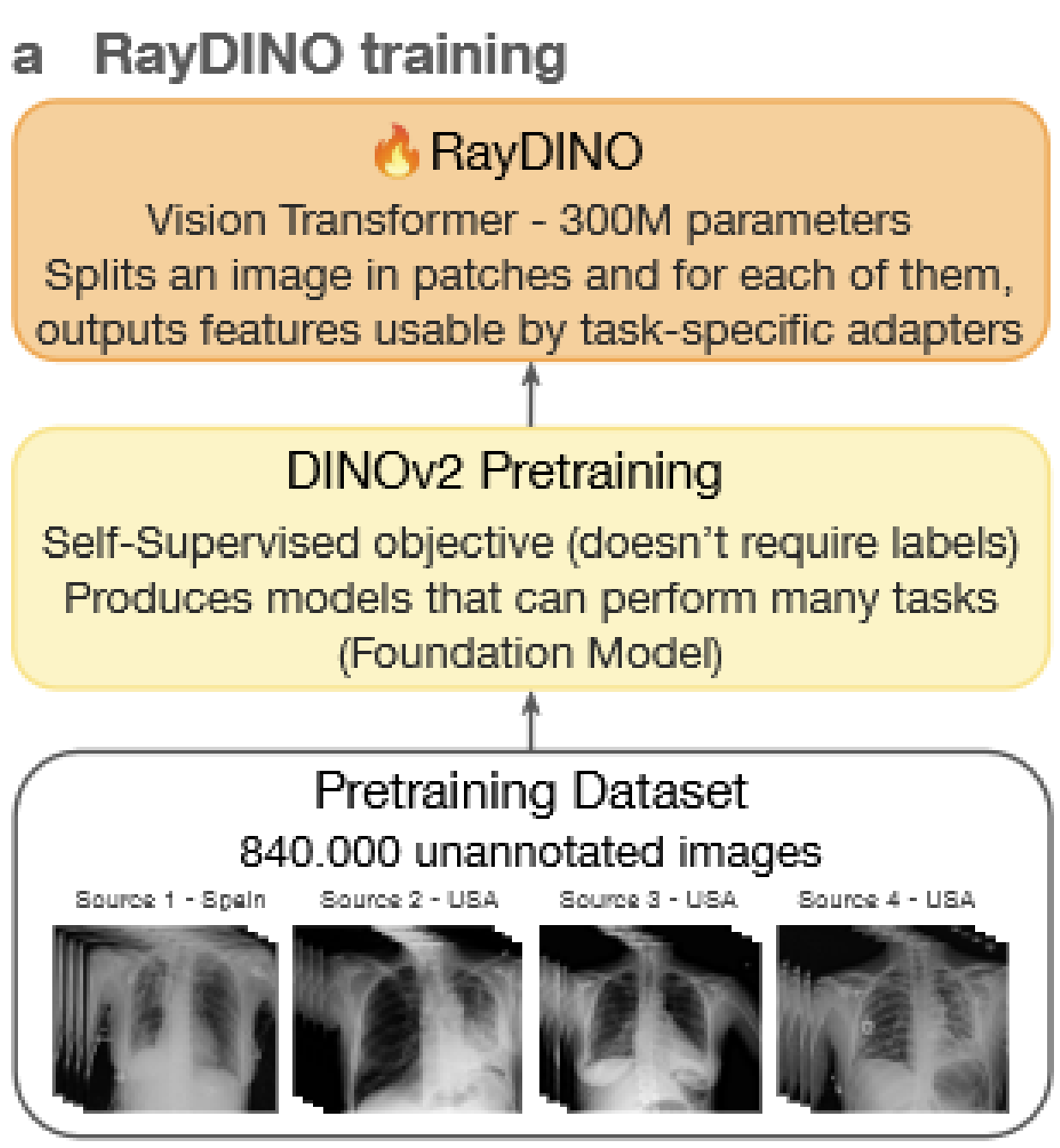

6. #2) Another significant issue is the tone of the manuscript. This is ultimately an editorial issue, but the tone struck me as inappropriate for a scientific journal. It is very possible that all the superlatives used may turn out to be fair for such a potentially useful tool, but one it not used to this tone. Also, while the text seems to heavily promote the tool, the paper lacks a clear description of how why and how it is better. Even if it is really as good as advertised, the proof is in the details, which are not given enough explanation.. Some examples;

   ABSTRACT
   "state-of-the-art results"
   -no specific meaning of state of the art

   INTRODUCTION
   "RayDINO surpasses other task-specific supervised models"

-This comment is made in the introduction without any references, additionally, surpasses is non-specific. If it has previously been shown to have a better performance in a task, name the task and the reference.
"As part of our extensive quantitative evaluation,"
-can leave out "extensive""

DISCUSSION
"Impressive performances with respect to other foundation methods and the added value of RayDINO."
-Can leave out "impressive"

Response

We are sorry if you found the tone inappropriate, we did not intend to present a paper with editorial issue but a true scientific contribution. RayDINO is the largest and current state of the art foundation model for XRay analysis, according to our knowledge. We tried our best in this updated version of our manuscript to give better definitions and explain in more detail the different parts of our method. Our extensive empirical evaluation of our foundation model for a variety of tasks and problems compared to the other state-of-the-art methods highlights numerically its superiority with respect to the other models that exist in the literature. We also want to emphasize that no other article tests their model on more than a few benchmarks (usually less than 10) and way less than 9 tasks like we do (Zhou et al. 2022, Zhang et al. 2023, Tiu et al. 2023, Ma et al. 2023, Azizi et al. 2023, Seibold et al. 2023, Dai et al. 2024). We hope that the added details on the different parts of the manuscript, including introduction, results and and dataset section, highlight this point and the impact of our work.

P7 L206-218 Discussion: "By using recent techniques for scaling self-supervision based on DINOv2, we developed RayDINO, a state-of-the-art model that can analyze chest X-rays in a holistic manner, trained in a patient-centric way using only the imaging data rather than annotations that follow country-specific guidelines. Recently, several models presented in the literature proposed general representations of X-ray imaging using either imaging, or coupling imaging with text, evaluated on different tasks. We have proven RayDINO's superiority over these models on several radiology tasks. In addition, it has been assessed on more holistic and clinically relevant issues compared to other methods for X-ray image processing (REMEDIS, Rad-Dino), showing better adaptation to unseen tasks, with few labels and good localization, staying more robust in new populations and better mitigating bias inherent to the data. To the best of our knowledge, our study is the first to train the largest self-supervised method on 840.000 X-rays and test it across 9 tasks on 21 different benchmarks comprising more than 200 categories in both internal and external settings, from patients of seven countries across four continents, and reaching good interpretability with state-of-the-art results in the process. Previous work focused on populations from one country, few benchmarks, or without completely external validation. Our extensive evaluation, audit RayDINO's ability to handle multiple tasks across diverse demographics, providing more insights about performance and limitations of current foundation models."

7. #3) Some evaluation on Small Datasets: In Figure 1B, the authors mention evaluating their model using various datasets such as JSRT (154 images), Scoliosis (609 images), and VinDr Rib (245 images). These datasets are relatively small for evaluating deep learning models, which typically

require larger datasets to demonstrate robustness and generalization. The small sample sizes call into question the validity of their evaluation and whether their model can generalize effectively to broader clinical settings.

Response

We respectfully disagree with your comment about the datasets used for evaluation of our model. Our study provides the most complete and diverse evaluation with respect to publicly available datasets and tasks for X-Ray analysis. While we do agree that the three datasets you mentioned are relatively small, our manuscript includes another 10 datasets with a much larger dataset size. Small datasets, including JSRT and VinDr Rib, have been used in recent work to evaluate foundation models as well (Table 3 from Dai et al. 2024 or Table 1 from Ma et al. 2023).

RayDINO consistently outperforms the other benchmarked models in all small and bigger datasets. We insist on including the JSRT, Scoliosis, and VinDr Rib datasets due to the challenging tasks they handle (segmentation masks and scoliosis angles) to highlight the generalizability of our holistic representations. Please note that even if these results were not included in the paper, the main claims and impact of RayDINO, as the current state-of-the-art model, still hold.

One point that we would like to highlight, and we hope that it is more clear in the current version of our manuscript, is that the datasets used are split into two different training schemes. The datasets that are used for the pretraining of our foundation model (MIMIC, CheXpert, NIH and PadChest) compose a pool of 849k chest X-Ray images. These datasets were selected due to their very large size, which allows the training of the self-supervised learning scheme without the need for any annotation. For the evaluation of our representations, we used different downstream tasks that indeed required training with labels. We hope that the use of each dataset is better highlighted in the additional Table 1 in the Online Methods. For each of these evaluations however, the RayDINO representations are frozen, and this facilitates the training of very small and light with respect to parameter adapters that do not require so much data for their convergence. In particular, each of these adapters requires a fraction of the parameters in comparison to RayDINO. This is, indeed, one of the main contributions of our model: the ability to provide holistic representations that are easily adaptable to various tasks without the need for many annotations, compared to fully supervised settings. We believe that this is demonstrated by our experiment on the Scoliosis dataset, on which a very small adaptor (<1% of RayDINO's and other models' parameters) was enough to perform better than other supervised pipelines such as the ARK6 model. This showcases that you don't need full finetuning of the model anymore, reducing the need for expensive training and loads of data. All the details about the adaptors are included in Table 3.

Finally, we would like to mention that two of these small datasets JSRT and VinDr Rib as well as the BRAX were used as completely external test sets for the evaluation of the different benchmarks of RayDINO. In particular, JSRT and VindrRib were used to evaluate the segmentation adapters trained on RAXRay dataset (Figure 4b), while BRAX was used for the same evaluation for the classification adapters trained on (Figure 4a). These evaluations prove the ability of small adapters trained using the RayDINO representations to generalize and work very well on these completely external datasets. We are one of the first in the literature on foundation models that challenge the capabilities of such models in completely independent datasets that do not even need the use of adaptors.

P10 Table 1

P13 Table 3

Model	Architecture	Num. Params	Pre-Train Datasets
Benchmarked Backbones			
UniChest[37]	ResNet50	55 M	CheXpert, MIMIC, NIH, VinDr
KAD[36]	ResNet50	55 M	MIMIC
ARK6[38]	Swin-B	91 M	CheXpert, MIMIC, NIH, VinDr, Pneumonia, Shenzhen
CheXzero[35]	ViT-B	86 M	MIMIC
Refers[34]	ViT-B	86 M	MIMIC
RayDINO (ours)	ViT-L	307 M	CheXpert, MIMIC, NIH, PadChest
Task-Specific Adapters			
Classification / Regression Adapter	2 layers MLP + ReLU	0.1-1 M	-
Segmentation Adapter	UperNet[52]	49 M	-
Text Adapter	LLM (Llama[53])	7 B	-

Table 3. **Parameter size** of the different backbones we use in this study along with the task-specific adapters.

P8 Figure 4 a, b

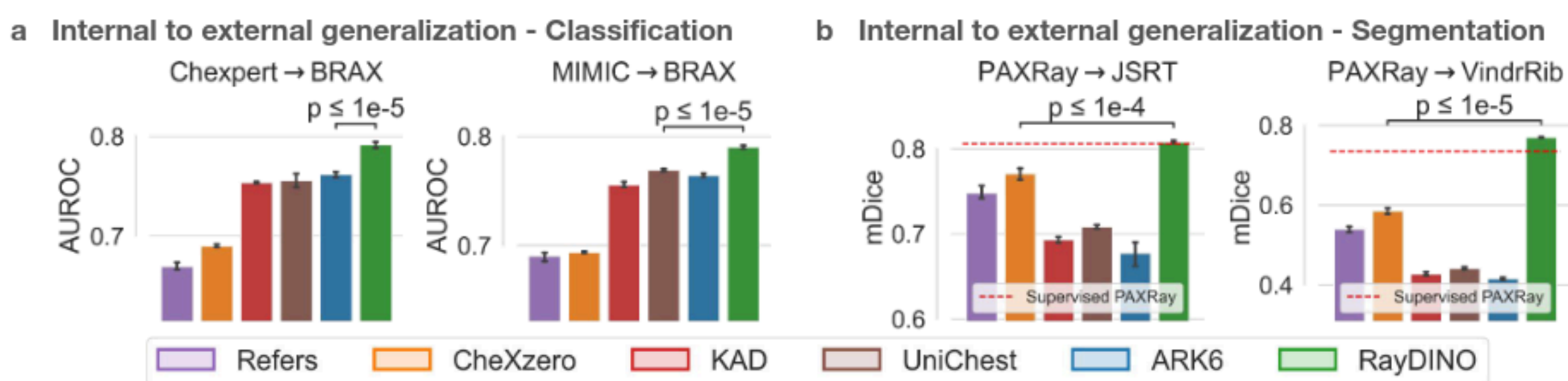

8. <u>#4) The role of Meta needs to be clarified. Meta is not listed as a source of funding for the study which is not possible as it's employees worked on the study. The following needs to be clarified;</u>

   <u>-What did Meta contribute to the study?</u>
   <u>-Is RayDino a Meta product?</u>
   <u>-What was the role of Meta in writing the manuscript?</u>

   Response

   Sorry for the missing information; we added more details in the article. The manuscript has been written in part by researchers from the Meta FAIR research laboratory, and it is not an official Meta product. Only authors stated in the authors' list have contributed to the manuscript. We also emphasize that the pre-trained RayDINO model will be made available with a download link for research purposes and to help the creation of better AI for X-rays analysis.

   P17 L515-516 "Meta provided computing resources and RayDINO is not an official Meta product."

Nature NCOMMS-24-52109A - Response to Reviewers

Thank you for your valuable feedback and for guiding the review process. We appreciate the constructive comments from the reviewers and have thoroughly addressed the remaining concerns. In addition, we have now released our model code https://github.com/facebookresearch/dinov2 and weights https://ai.meta.com/resources/models-and-libraries/raydino-downloads/ to ensure reproducibility and transparency. Moreover, we have performed extra statistical tests between the different methods, highlighting the power and robustness of our models across tasks and datasets. All changes are highlighted in the answers of the reviewers, as well as in red in the main manuscript.

Reviewer #1 (Remarks to the Author):

The authors have adequately addressed the concerns raised in the previous review cycle. I have no further comments to make.

We thank the reviewer for his previous insightful comments, which have considerably improved the clarity and presentation of our work.

Reviewer #2 (Remarks to the Author):

1. I think the paper improved a lot from the last revision, here is some minor comments.

Response: Thank you for your insightful feedback regarding our paper and your additional comments that help the clarity and presentation of our manuscript. We have carefully revised the manuscript addressing your minor comments. We believe these revisions enhance the clarity and rigor of our work and look forward to your further evaluation.

2. L36: "Our model generates holistic representations of a compact size of 1024 for each…" it is clear from the context, but somebody outside of computer science domain may not understand what 1024 refers to.

Response: Thank you for bringing that to our attention. We changed the original text to make it clearer.

L37 to "Our model generates holistic representations of a compact size of 1024 embedding dimensions for each chest X-rays"

3. L84: I would better specify that other compared models are fully-supervised and so their parameters are optimized on that specific task.

Response: Thank you for the suggestion. We agree that this change will clarify and enhance the presentation of our comparisons. We changed L84 to now P3 L61-62 to the following

Furthermore, despite other compared models being supervised on this specific task and RayDINO having 100x less supervised parameters (Table 3), …

4. <u>I would maybe better stress the role of the adapter between images and text. As far as it is written, text and images are not aligned, which makes the result even more impressive.</u>

Response: We apologize if this part is unclear. Indeed, RayDINO has been pretrained using only images, and its very good performance on report generation makes its representation power even more impressive. To stress this, as you suggested, we added the following clarification.

P3, L79 *We want to emphasize that RayDINO is an image-based foundation model, contrary to KAD, UniChest, and CheXzero compared methods.*

5. <u>Did you verify the presence of hallucinations in the report generation?</u>

Response: Thank you for the very interesting comment. Indeed, hallucinations of LLMs are a crucial problem, especially for medical report generation. In this study, we utilize this task as an additional benchmark to highlight the capabilities of our RayDINO model. To evaluate performance, we utilized standard and widely used Natural Language Processing metrics, and we did not further investigate or evaluate the presence of hallucinations. However, we appreciate your suggestion and will consider it for future work, in order to boost the use of foundation models on this very clinically important task.

<u>Reviewer #3 (Remarks to the Author):</u>

1. <u>Regarding the manuscript in general, some has improved, but a lot has been added, some of which needs to get cleaned up.</u>

Response: We would like to thank you for your valuable feedback and constructive comments that helped us clarify points with respect to the used datasets, evaluations, and overall enhance the clarity of our method and work. We have carefully revised the manuscript and provided comprehensive answers to address your additional concerns. We believe these revisions enhance the clarity and rigor of our work and look forward to your further evaluation.

2. <u>The introduction is full of methods and results. Figure 1 and its explanation are in the Introduction and includes both methods and results. The strategy of the loss functions is also in the introduction.</u>

Response: Thank you for helping us refactor our paper to enhance its structure and clarity. We revisit the Introduction section of all superfluous methods and results (P1, L34), making the paper much easier to follow. We have removed the loss function description from the Introduction, so it is now only available in the Methods section (P9, L252-258). We have moved details about the datasets used in the Methods section as well (P9-10, L275-280). We added a new paragraph (P2-3, L30-47) to the results section to present the baselines, evaluation settings, and main results of Figure 1. Finally, we also moved Fig. 1 to the results section to clarify the presentation.

P2, L40-43: We evaluate RayDINO's performance (Fig. 1a) by using task-specific adapters on top of the obtained frozen features (Fig. 1b,c) using eleven publicly available datasets from seven countries across four continents, both in internal and external settings. We report an extensive evaluation on nine medical-imaging related tasks (Fig. 1d) divided into 21 benchmarks comprising more than 200 classes (Fig. 1e). We compare our model with five state-of-the-art foundation models trained using supervised annotations: Refers, CheXzero, KAD-512, UniChest, and ARK6. We emphasize that these models are fully fine-tuned end-to-end using in-domain labels or reports, while our approach uses a frozen backbone pre-trained without any human supervision. This establishes a challenging benchmark for RayDINO, which has significantly fewer supervised parameters than the baselines (Table 3)."

3. The "conclusion" is now in the introduction, "Our findings indicate that RayDINO's representation provides state-of-the-art performance on all 21 benchmarks by surpassing the best available chest X-ray foundation models in the literature on most of them (Fig. 1d), without relying on human annotations during its pre-training phase." This is a conclusion based on the results. It should not be in the introduction. Especially the comment that it surpasses the other foundation models, without any references to the other models.

Response: We are sorry for the confusion. We moved the concerned section from the Introduction to the Discussion (P8, L18).

P08, L188: Our findings indicate that RayDINO's representation provides state-of-the-art performance on all 21 benchmarks by surpassing the best available chest X-ray foundation models in the litterature on most of them (Fig. 1d), without relying on human annotations during its pre-training phase. In addition, RayDINO handles better rare categories, worst minority groups, and external demographics. Finally, as an additional source of information, our model also provides stronger interpretability, qualitatively validated by expert radiologists and quantitatively using attention pinpointing.

4. It looks like comparative hypothesis testing was done in a very unusual way. First it looks like a t-test was used to compare summary statistics. The t-test is only designed for specific uses, not to compare all summary statistics. The t-test was not designed to compare AUROCs for instance, not Pearson's correlation coefficients. Additionally, it looks like some type of bootstrapping was done by repeating a measurement only done 5 times. This needs to be clarified with the exact process used and why it was only repeated 5 times, other than a typical number of at least 1000 for the larger datasets.

Response: We appreciate the reviewer's attention to this important point. Indeed, in this study, we performed a double t-test on AUROC scores across different random seeds during training of the adapters. We agree that the t-test is not theoretically ideal for all types of summary statistics, including AUROC. However, in the machine learning literature, it is common practice to apply the t-test (or similar statistical tests) over metrics like AUROC when models are trained multiple times with different random seeds, in order to assess the consistency and robustness of observed performance differences. Given the sensitivity of representation learning to initialization and data sampling, we believe it is meaningful to assess consistency over multiple

seeds and report statistical comparisons across them. Our use of the t-test follows precedent in related high-impact work [1,2,3], where t-tests were applied to AUROC values across repeated runs for comparisons of different models. We also want to add that no bootstrapping has been done other than for the report generation metrics, and that we used a number of 500 (figure S1). While we acknowledge the theoretical limitations of the t-test when comparing individual AUROC, we used it primarily as a pragmatic measure of robustness of our model's representations given multiple seeds rather than strict inference, and will clarify this in the manuscript.

However, following the reviewer's comment and in order to make things more clear, we have re-performed all statistical tests using DeLong's test [4] for AUROC comparisons and a paired permutation test for the remaining performance metrics (1000 bootstraps and 2.5-97.5 percentiles). All updated statistical results have been incorporated into the manuscript and figures, and the revisions are highlighted in red. We would also like to note that, although the statistical tests have been updated, the conclusions remain unchanged: our proposed RayDINO method still significantly outperforms the compared approaches. An overview of all the new statistical tests is summerized in Table 4, while all our previous double t-test on AUROC scores across different random seeds are moved to the supplementary Figures 32-37.

Task	Dataset	Num. Class.	Num. classes with better performances: RayDINO ($p < 0.05$)	NS ($p \geq 0.05$)	Other Best Model ($p < 0.05$)	Other Best Model
Classification	MIMIC	14	5	9	0	UniChest
	NIH	15	8	7	0	ARK6
	VinDr	27	3	18	6	UniChest
	CheXpert	14	1	12	1	UniChest
	Total	70	17	46	7	-
Rare Class Prediction	MIMIC Long-Tail	26	22	4	0	UniChest
	Catheter Malposition	9	8	1	0	ARK6
	Total	35	30	5	0	-
Segmentation	JSRT	5	5	0	0	CheXzero
	PAXRay	159	152	7	0	CheXzero
	Pneumothorax	1	1	0	0	UniChest
	VinDr Rib	20	20	0	0	Refers
	Total	185	178	7	0	-
Generalization Classification	CheXpert → BRAX	14	8	5	1	ARK6
	MIMIC → BRAX	14	8	6	0	UniChest
	Total	28	16	11	1	-
Generalization Segmentation	PAXRAY → JSRT	5	3	2	0	CheXzero
	PAXRAY → VinDr Rib	20	20	0	0	CheXzero
	Total	25	23	2	0	-
Generalization Task & Disease	Scoliosis	3	3	0	0	ARK6
	PolCovid	3	2	1	0	ARK6
	Total	6	5	1	0	-
Attention Pinpointing	VinDr	20	5	14	1	UniChest
	Total	20	5	14	1	-
Total		369	274	86	9	-

Table 4. Statistical comparison of RayDINO and the best other model for each task, dataset and class. We use a DeLong test for AUROC comparisons and a paired permutation test for all other metrics.

More specifically, we can see that RayDINO demonstrates strong and consistent performance across various tasks and datasets. When comparing RayDINO to the best baseline model for each class (considering all classes and datasets), RayDINO is superior for 274 classes, inferior for only 9 classes, and not significantly different (NS) from the best baseline for 86 classes. We also want to emphasize that we always compare RayDINO to the best other model, but this changes depending on the task/dataset and such models are most of the time fully supervised. While UniChest and CheXzero are strong models, RayDINO is consistently strong across all classes from all datasets and tasks, as shown in the supplementary figures S8-31. Consistency is the strength of RayDINO (as strong as supervised models on in-domain data, better for generalization, bias, and other tasks), and RayDINO doesn't overfit like supervised models.

[1] Zhou, Y., Chia, M.A., Wagner, S.K. et al., A foundation model for generalizable disease detection from retinal images. **Nature** 622, 156–163 (2023)

[2] Zhou, HY., Chen, X., Zhang, Y. et al. Generalized radiograph representation learning via cross-supervision between images and free-text radiology reports. **Nature Machine Intelligence** 4, 32–40 (2022). https://doi.org/10.1038/s42256-021-00425-9

[3] Azizi S, Culp L, Freyberg J, Mustafa B, Baur S, Kornblith S, Chen T, Tomasev N, Mitrović J, Strachan P, Mahdavi SS, Wulczyn E, Babenko B, Walker M, Loh A, Chen PC, Liu Y, Bavishi P, McKinney SM, Winkens J, Roy AG, Beaver Z, Ryan F, Krogue J, Etemadi M, Telang U, Liu Y, Peng L, Corrado GS, Webster DR, Fleet D, Hinton G, Houlsby N, Karthikesalingam A, Norouzi M, Natarajan V. Robust and data-efficient generalization of self-supervised machine learning for diagnostic imaging. **Nature Biomedical Engineering**. 2023 Jun;7(6):756-779. doi: 10.1038/s41551-023-01049-7. Epub 2023 Jun 8. PMID: 37291435.

[4] DeLong, E. R., DeLong, D. M. & Clarke-Pearson, D. L. Comparing the areas under two or more correlated receiver operating characteristic curves: A nonparametric approach. Biometrics 44, 837–845 (1988)

Summary of changes:

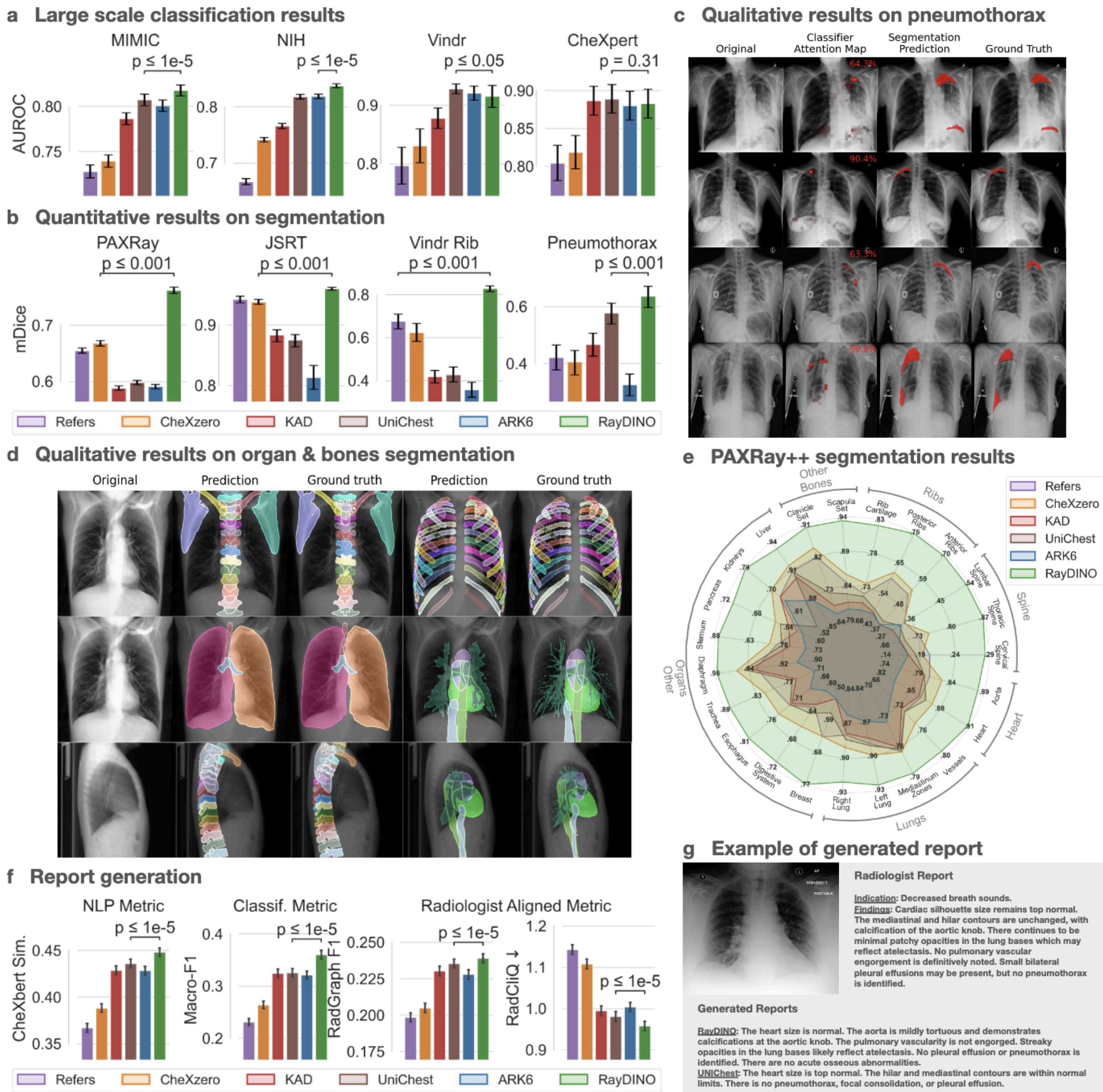

Figure 2. **RayDINO's holistic analysis of chest X-rays.** **a)** AUROC comparisons on 4 classification datasets from the USA and Vietnam including 38 different findings. **b)** mDice comparisons on 4 segmentation datasets from Japan, Vietnam, the USA, China and multiple other countries including 157 categories. **c)** Qualitative visualisation of RayDINO's prediction on four pneumothorax cases. **1st column**: original image. **2nd column**: interpretable attention maps showing where the classifier trained without pixel supervision is looking at. **3rd column** segmentation prediction trained using pixel supervision. **4th column**: radiologist ground-truth. **d)** Qualitative results for organ and bone segmentation on the PAXRay++ dataset. **e)** mDice segmentation comparisons macro-averaged per organs and bones. **f)** Report generation comparison including natural language processing metric, classification-based metric and radiologist-aligned metrics. **g)** A chest X-ray along with a radiologist report and two generated reports by RayDINO and UNIChest.

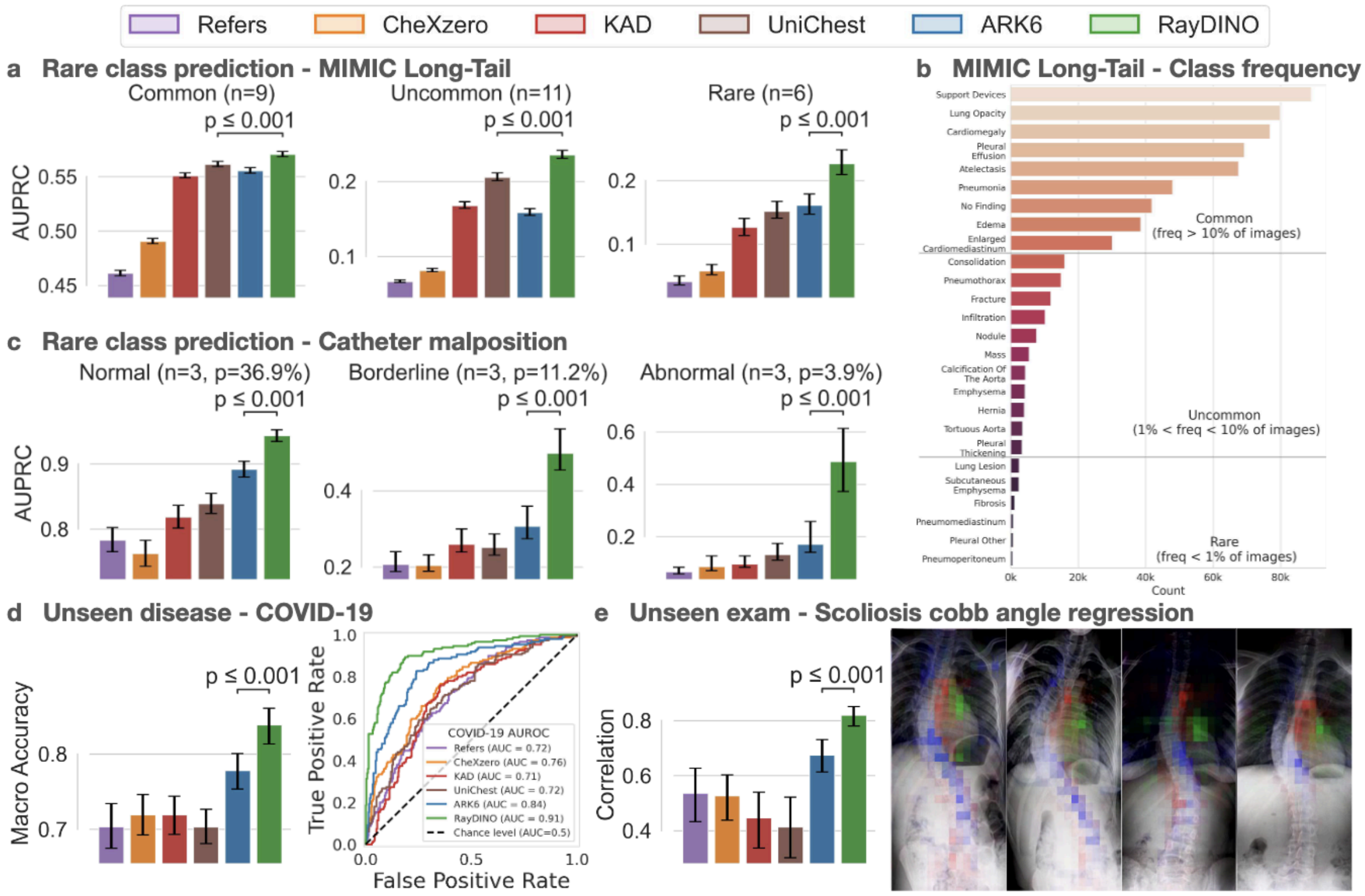

Figure 3. Generalization evaluation on rare or unseen diseases and on unseen exam. **a)** AUPRC comparisons of RayDINO against other models on long-tailed findings grouped by frequency: Common ($freq > 10\%$), Uncommon ($1\% < freq < 10\%$) and Rare ($freq < 1\%$). **b)** Class distribution on the long-tail evaluations sorted by frequency. **c)** AUPRC comparisons on catheter malposition prediction. **d)** Macro Accuracy comparison on Normal vs Pneumothorax vs COVID-19 classification and ROC curves for the COVID-19 disease. **e)** Pearson's correlation coefficient comparison on Cobb angle regression using spinal exams on patients with scoliosis and the regression model's multi-head attention maps for interpretability.

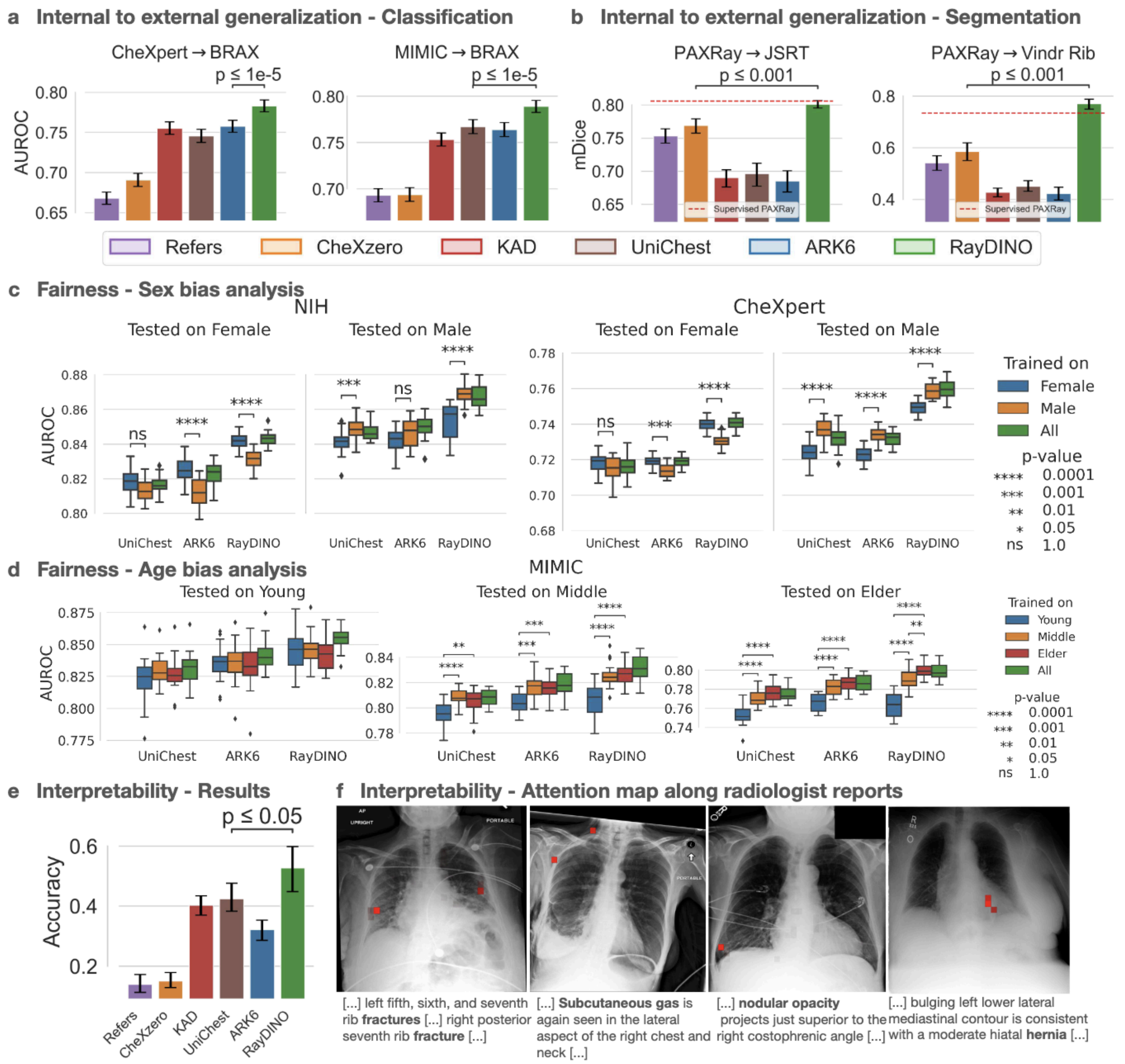

Figure 4. Interpretability and Fairness evaluation of RayDINO between different demographics. a) AUROC comparisons by training and validating a classifier on MIMIC or CheXpert (USA) and testing it on BRAX (Brazil). **b)** mDice comparisons by training and validating a segmentation head on PAXRay++, a synthetic dataset generated from 2D CT-scan projections (mainly the USA and China), and testing it on real world datasets JSRT (Japan) and Vindr Rib (Vietnam). **c)** AUROC fairness comparisons by training a classifier on one sex-specific split and testing it on the other split of NIH and CheXpert datasets. p-values obtained with a Mann-Whitney test to assess performance disparities when training with the same sex versus a different sex than the test set. **d)** AUROC fairness comparisons by training a classifier on one age-specific split and testing it on the two other splits of the MIMIC datasets. **e)** Macro Accuracy interpretability comparisons by training one classifier for each class of Vindr and by looking if the position where the attention is looking the most match the radiologist bounding box on the test set. **f)** Attention maps comparison with radiologist reports to demonstrate the accurate localization of RayDINO's explainable classifiers (specific finding highlighted in bold in the report).

Fig 2, 3 and 4 included above

New Table 4

P3, L54-56: To compare RayDINO against the best performing baseline, we reported p-values of a DeLong test for AUROC and a paired permutation test for other metrics. More details about our metrics are available in the Evaluation Metrics section of Online Methods.

P3, L59-65 RayDINO significantly outperforms the best alternatives on the MIMIC dataset with .818 AUROC (95%CI [.812 -.824], +.011 above .807 of UniChest, $p \leq 1e{-4}$) and the NIH dataset with .837 AUROC (95%CI [.834 -.841], +.018 above .819 of ARK6, $p \leq 1e{-4}$). Furthermore, despite other compared models being supervised on this specific task and RayDINO having 100 x less supervised parameters (Table 3), it remains comparable on CheXpert with .883 AUROC (95%CI [.864 -.902], −.006 below .889 of UniChest, $p = .312$) and on Vindr which is out-of-domain for RayDINO, contrary to ARK6 and UniChest, with .915 AUROC (95%CI [.896 -.934], −.013 below .928 of UniChest, $p \leq 0.05$).

P3, L70-72: with .959 mDice on JSRT (95%CI [.957 -.962], +.021 above .938 of CheXzero, $p \leq 1e{-3}$), .828 mDice on Vindr Rib (95%CI [.814 -.840], +.152 above .676 of Refers, $p \leq 1e{-3}$), and .762 mDice on the more challenging PAXRay++ dataset (95%CI [.756 -.767], +.094 above .668 of CheXzero, $p \leq 1e{-3}$).

P3, L74: segmenting pneumothorax with .637 mDice (95%CI [.596 -.671], +.060 above .577 of UniChest, $p \leq 1e{-3}$).

P3, L96-99: RayDINO improves over the other state-of-the-art methods for common classes (.570 AUPRC, 95%CI [.568 -.573], +.009 above .561 of UNIChest, $p \leq 1e{-3}$), but more interestingly our model significantly outperforms all competitors on uncommon diseases (.235 AUPRC, 95%CI [.231 -.241], +.030 above .205 of UNIChest, $p \leq 1e{-3}$) and is way ahead on rare classes (.227 AUPRC, 95%CI [.210 -.248], +.066 above .161 of ARK6, $p \leq 1e{-3}$)

P4, L106-108: Normal (.944 AUPRC, 95%CI [.934-.952], +.052 above .892 of ARK6, $p \leq 1e{-3}$), Borderline (.498 AUPRC, 95%CI [.453 -.561], +.191 above .307 of ARK6, $p \leq 1e{-3}$) and Abnormal catheters (.486 AUPRC, 95%CI [.372 -.612], +.315 above .171 of ARK6, $p \leq 1e{-3}$).

P4, L114-116: .839 macro accuracy (95%CI [.813 -.861]). Our model significantly outperforms all other models (+.061 above .778 of ARK6, $p \leq 1e{-3}$) and is even better for the COVID-19 disease reaching .910 AUROC (95%CI [.900-.920], +.070 above .840 of ARK6, $p \leq 1e{-5}$)

P4, L120-121: RayDINO achieves a macro Pearson's correlation coefficient of .820 (95%CI [.780 -.851]) across the 3 Cobb angles, clearly outperforming alternatives with +.145 points over .675 of the best competitor ARK6 ($p \leq 1e{-3}$).

P4, L129-131 on the BRAX dataset when trained on CheXpert (.783 AUROC 95%CI [.776 -.790], +.025 above .758 of ARK6, $p \leq 1e{-4}$) and on MIMIC (.789 AUROC 95%CI [.783 -.796], +.022 above .767 of UniChest, $p \leq 1e{-4}$)

P4, L139-141: .801 and .770 mDice in Japanese and Vietnamese patients (95%CI [.795 -.807] and [.749 -.788], +.032 and +.185 above .769 and .585 of CheXzero, $p \leq 1e{-3}$ and $p \leq 1e{-3}$).

P8, L182-183: RayDINO outperforms all other model in term of localization, achieving a macro accuracy of .527 % (95%CI [.448 -.598], +.102 % above .425 % of UniChest, $p \leq 0.05$)

P14, L386-389: When comparing the performance of two models, to verify that the difference between their results is significative, we use either the DeLong test when computing AUROC, or a bootstrapped paired permutation test for other metrics (1000 bootstraps and $2.5 - 97.5$ percentiles). We report p-values for all our statistical tests, with low p-value meaning that the difference in performance is statistically significant.

P23, L609-614: Additional statistical tests between RayDINO and other models. In figures $S32 - 37$, We show the comparison of RayDINO and other models with different random seeds, in order to assess the consistency and robustness of observed performance differences. Given the sensitivity of representation learning to initialization and data sampling, we believe it is meaningful to assess consistency over multiple seeds and report statistical comparisons across them. We use a t-test across the 5 seeds of each models to compare them. We can see that all models show very low variability, and that RayDINO's performances are statistically significant across the range of all evaluations.

5. Regarding my prior issues, most have been addressed at least partially.

Response: We would like to thank you for your reviews, which really improved the presentation and clarity of our study. We have made our best effort to address your additional comments on this revised version of the paper.

6. In relation to my issues with the small data sets, I was not implying that they cannot be used for testing, but a comparison between models would be difficult. For example, the JSRT has 154 images, while the precision of the mDice for RayDino is .960 [0.959-.960]. This suggests an error of less than 0.001 in a sample of 154. This seems highly unlikely and is much smaller than errors that I have seen in other studies using mDice. This may be related to the confidence interval method noted above which I question.

Response: We apologize if our previous answer misinterpreted your question, and we appreciate your insightful comment. We would like to highlight that our study provides the most comprehensive and diverse evaluation with respect to publicly available datasets and tasks for X-ray analysis to date. We report metrics over models trained with 5 seeds and a hyperparameter grid search, whereas other works typically train only one model for each reported number.

Other articles also reported very small confidence intervals on JSRT. For example, the authors of the previous state-of-the-art model Ark introduced in [5] reported confidence intervals for mDice of "0.9748±0.0008; 0.9462±0.0016; 0.9005±0.0015" for the different classes of JSRT in Table 2 of their work. To clarify, our reported confidence intervals are derived from multiple independent training runs with different random seeds, rather than from bootstrapping the test set. Changing the seed has an impact on the model initialization and, more importantly, on the training set sampling. Since the dataset is relatively small, the variation captured by these intervals reflects uncertainty due to model initialization and training stochasticity, not variability in

the test data itself. This can sometimes result in narrower intervals than those based solely on test set sampling variability. We acknowledge that in small datasets, such intervals may underestimate the full uncertainty in performance metrics, however, they highlight the reproducibility and robustness of our representations even in very small test sets. To verify our claims, and as said in the answer to question 4, we have re-performed all statistical tests. We computed the 95% confidence interval on the two smallest datasets JSRT (154 images) and VinDr Rib (245 images) using 1000 bootstraps and 2.5 - 97.5 percentiles:

JSRT:
Seed based t-test (Previously reported): 0.960 [0.959 - 0.960] or [-0.001 ,+0.000]
New permutation test: 0.959 [0.957 - 0.962] or [-0.002, +0.003]

VinDr Rib:
Seed based t-test (Previously reported): 0.839 [0.823 - 0.855] or [-0.016, +0.016]
New permutation test: 0.828 [0.814 - 0.840] or [-0.014, +0.012]

We can see that the confidence intervals are of the same order of magnitude. We have clarified this distinction in the manuscript and discuss its implications for interpreting confidence intervals in such settings.

[5] DongAo Ma, Jiaxuan Pang, Michael B Gotway and Jianming Liang, Foundation Ark: Accruing and Reusing Knowledge, International Conference on Medical Image Computing and Computer Assisted Intervention 2023

P3, L54-56: To compare RayDINO against the best performing baseline, we reported p-values of a DeLong test for AUROC and a paired permutation test for other metrics. More details about our metrics are available in the Evaluation Metrics section of Online Methods.

P3, L70-72: with .959 mDice on JSRT (95%CI [.957 -.962], +.021 above .938 of CheXzero, p ≤ 1e−3), .828 mDice on Vindr Rib (95%CI [.814 -.840], +.152 above .676 of Refers, p ≤ 1e−3)

P14, L386-389: When comparing the performance of two models, to verify that the difference between their results is significant, we use the DeLong~\cite{DeLong1988ow} test when computing AUROC. Moreover we also report a bootstrapped paired permutation test for other metrics (1000 bootstraps and $2.5-97.5$ percentiles). We report p-values for all our statistical tests, with low p-value meaning that the difference in performance is statistically significant.

P23, L609-614: Additional statistical tests between RayDINO and other models. In figures S32 − 37 , We show the comparison of RayDINO and other models with different random seeds, in order to assess the consistency and robustness of observed performance differences. Given the sensitivity of representation learning to initialization and data sampling, we believe it is meaningful to assess consistency over multiple seeds and report statistical comparisons across them. We use a t-test across the 5 seeds of each models to compare them. We can see that all models show very low variability, and that RayDINO's performances are statistically significant across the range of all evaluations.

7. Lastly I am happy to hear that Meta was listed as providing computer resources. I do not understand why Meta is allowing its employees to work on this project in their computing environment. I have no reason to think that this is necessarily a problem, but I think that it would be best to just list this as a competing interest as the code also sits in the Meta domain

   Response: We appreciate your guidance and are grateful for your support in enhancing the clarity and transparency of our research. We are committed to maintaining transparency and integrity in our work. This work was a very nice and fruitful collaboration between Meta, University Paris Saclay and APHP public hospitals of France. In this interdisciplinary work, we are pleased to have provided open-source code and models (now publicly available: https://github.com/facebookresearch/dinov2) that can help the community advance in this important and challenging task. The contribution of Meta's resources, made it possible to conduct this very extensive study that includes most of the publicly available X-Ray datasets and the most diverse tasks. We hope this effort helps pave the way for future collaborations that bridge academia and industry.

   We will ensure that Meta's provision of computing resources and the involvement of its employees are clearly disclosed as a competing interest in our manuscript.

   P17, L507: "Some of the researchers involved in this study are part of Meta."
